# Supplementary material for: System-wide Profiling of RNA-Binding Proteins Uncovers Key Regulators of Virus Infection
Source: Mol Cell. 2019 Apr 4;74(1):196–211.e11. doi: 10.1016/j.molcel.2019.01.017 (PMC6458987; doi:10.1016/j.molcel.2019.01.017)
Supplement: Document S1. Figures S1–S7 [file mmc1.pdf]

Molecular Cell, Volume *74*

Supplemental Information

System-wide Profiling of RNA-Binding Proteins Uncovers Key Regulators of Virus Infection

Manuel Garcia-Moreno, Marko Noerenberg, Shuai Ni, Aino I. Järvelin, Esther González-Almela, Caroline E. Lenz, Marcel Bach-Pages, Victoria Cox, Rosario Avolio, Thomas Davis, Svenja Hester, Thibault J.M. Sohier, Bingnan Li, Gregory Heikel, Gracjan Michlewski, Miguel A. Sanz, Luis Carrasco, Emiliano P. Ricci, Vicent Pelechano, Ilan Davis, Bernd Fischer, Shabaz Mohammed, and Alfredo Castello

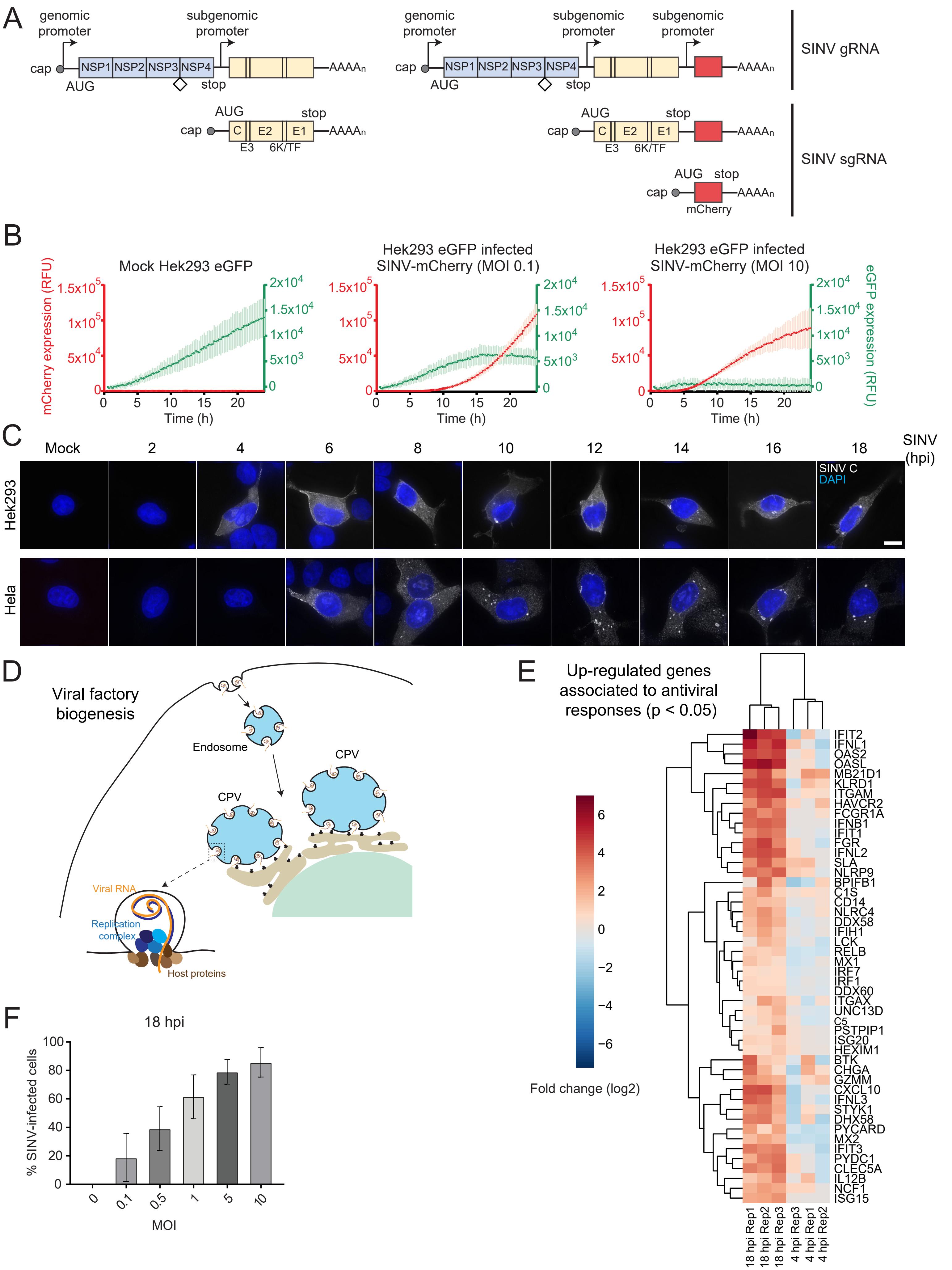

Figure S1 (Garcia-Moreno et al)

Figure S1. Analysis of SINV-induced molecular signatures in different cellular models; related to Figure 1. A) Schematic representation of SINV genomic (g) and subgenomic (sg) RNAs. The diamond represents the opal termination codon between NSP3 and NSP4. B) Production of eGFP and mCherry in stable Tet-on HEK293-eGFP cells infected with SINV-mCherry at 0.1 or 10 multiplicity of infection (MOI). Green and red fluorescence were measured every 15 min in a plate reader with atmospheric control (5% CO_2 and 37°C). eGFP synthesis serves as a proxy of host cell gene expression. Virus-induced shut off is reflected by the blockage of eGFP accumulation, which occurs at 5 hpi with 10 MOI (in agreement with the [^{35}S]-Met/cys labelling analysis shown in Figure 1C) and at 15 hpi with 0.1 MOI. RFU, relative fluorescence units. C) Localisation by immunofluorescence of SINV C in SINV-infected HEK293 and Hela cells. SINV C accumulates in cytoplasmic foci known as the viral replication factories, which are detected in both HEK293 and HeLa at similar times post infection. D) Schematic representation of viral factory biogenesis. CPV, cytopathic vacuoles containing spherules where viral RNA is replicated. E) Heatmap representing the log2 fold expression change determined by RNAseq analysis of RNAs enriched with $p<0.05$ in SINV-infected (4 or 18 hpi) versus uninfected HEK293 cells and annotated by 'antiviral response' and 'innate immunity' gene ontology (GO) terms. These GO terms were statistically enriched in infected over uninfected cells. Note the presence of interferons (IFNs), interferon stimulated genes (ISG), interferon induced proteins (IFI), and interferon regulatory factors (IRF). F) Percentage of HEK293 cells expressing SINV C after infection with SINV-mCherry at different MOI. Cells were processed for immunofluorescence staining with α-SINV C antibody and DAPI. 10 fields were counted for each independent experiment (n=3). Bars represent the mean and error bars represent the standard deviation (SD).

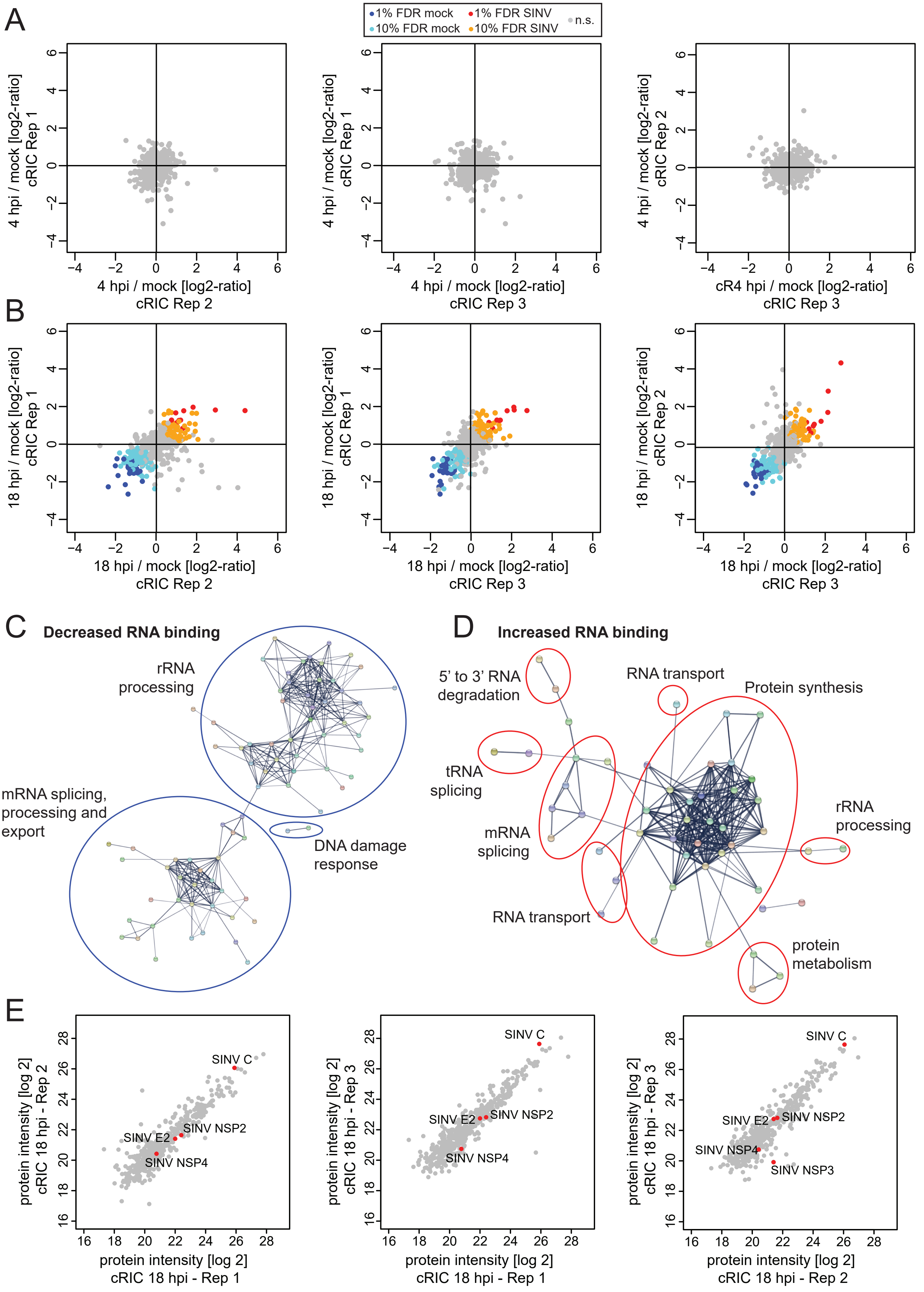

Figure S2 (Garcia-Moreno et al)

Figure S2. Analysis of the RNA-bound proteome in SINV-infected HEK293 cells by cRIC; related to Figure 2 and Table S1. A) Scatter plots displaying the intensity ratio for each protein (dots) in the cRIC eluates from cells infected with SINV for 4 h over uninfected cells, analysed by quantitative proteomics. Plots show the experimental reproducibility as each axis indicate the ratios obtained in one biological replicate. The three scatter plots represent the pair-wise comparison of the three replicates. B) As in (A) but comparing 18 hpi versus uninfected cells. Proteins enriched in infected cells with 1% or 10% FDR are shown in red and orange, respectively. Proteins enriched in uninfected cells with 1% or 10% FDR are shown in blue or cyan, respectively. Grey dots represent non-enriched proteins. FDR, false discovery rate; n.s. non-significant. C) STRING network showing the protein-protein interaction map between SINV-inhibited RBPs (both 1% and 10% FDR). D) As in (C) but with SINV-stimulated RBPs. E) Scatter plots comparing the raw intensity of each protein in the eluates of cRIC from cells infected with SINV for 18 h. Viral proteins are highlighted in red. The three scatter plots display the pair-wise comparison between all three replicates.

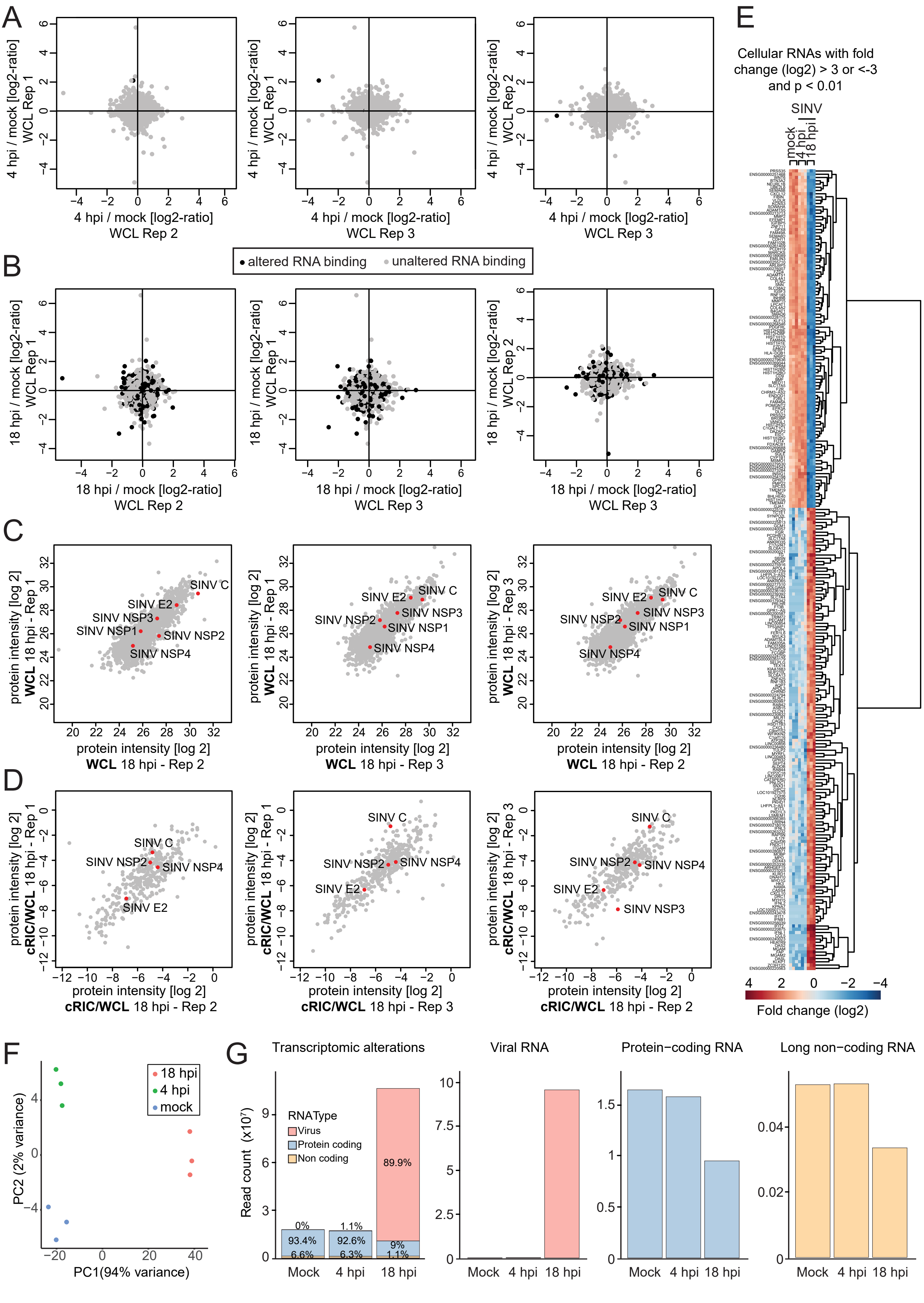

Figure S3 (Garcia-Moreno et al)

Figure S3. Proteomic and transcriptomic analyses of whole SINV-infected cell lysates; related to Figure 3 and Tables S3-4. A) Scatter plots displaying the intensity ratio for each protein (dots) in the cRIC inputs from cells infected with SINV for 4 h over uninfected cells, analysed by quantitative proteomics. Plots show the experimental reproducibility as each axis indicate the ratios obtained in one biological replicate. The three scatter plots represent the pair-wise comparison of the three replicates. Black dots represent proteins significantly enriched in the eluates of cRIC experiments either at 4 hpi or in uninfected conditions (Figure 2A and S2A). WCL, whole cell lysate. B) As in (A) but between 18 hpi and uninfected conditions. Black dots represent proteins significantly enriched in the eluates of cRIC experiments either at 18 hpi or in uninfected conditions (Figure 2C and S2B). C) Scatter plots comparing the raw intensity of each protein in the inputs of two cRIC replicates from cells infected with SINV for 18 h. The three scatter plots display the pair-wise comparison between all three replicates. Viral proteins are highlighted in red. D) Scatter plots comparing the intensity of each protein in the cRIC eluates normalised to the intensity in the inputs of two biological replicates from cells infected with SINV for 18 h. The three scatter plots display the pair-wise comparison between all three replicates. E) Heatmap representing the differential expression of cellular RNAs detected by RNAseq in SINV-infected (4 or 18 hpi) and uninfected HEK293 cells. Only RNAs with log2 fold change >3 or <-3 and $p<0.01$ in mock to infected comparisons are displayed. F) Principal component analysis of gene expression profiled by RNAseq in uninfected and SINV-infected cells at 4 or 18 hpi. The three replicates of each condition are considered separately. Data shows that, first, replicates from the same condition cluster together (i.e. they are more similar to each other than to other conditions) and, second, that the transcriptome at 18 hpi strongly differs from the uninfected control and 4 hpi. G) Read counts for viral RNA, cellular protein-coding RNA and cellular non-coding RNA quantified by RNAseq in mock and SINV-infected cells. These plots show the changes in the RNA composition of the cells as infection progresses.

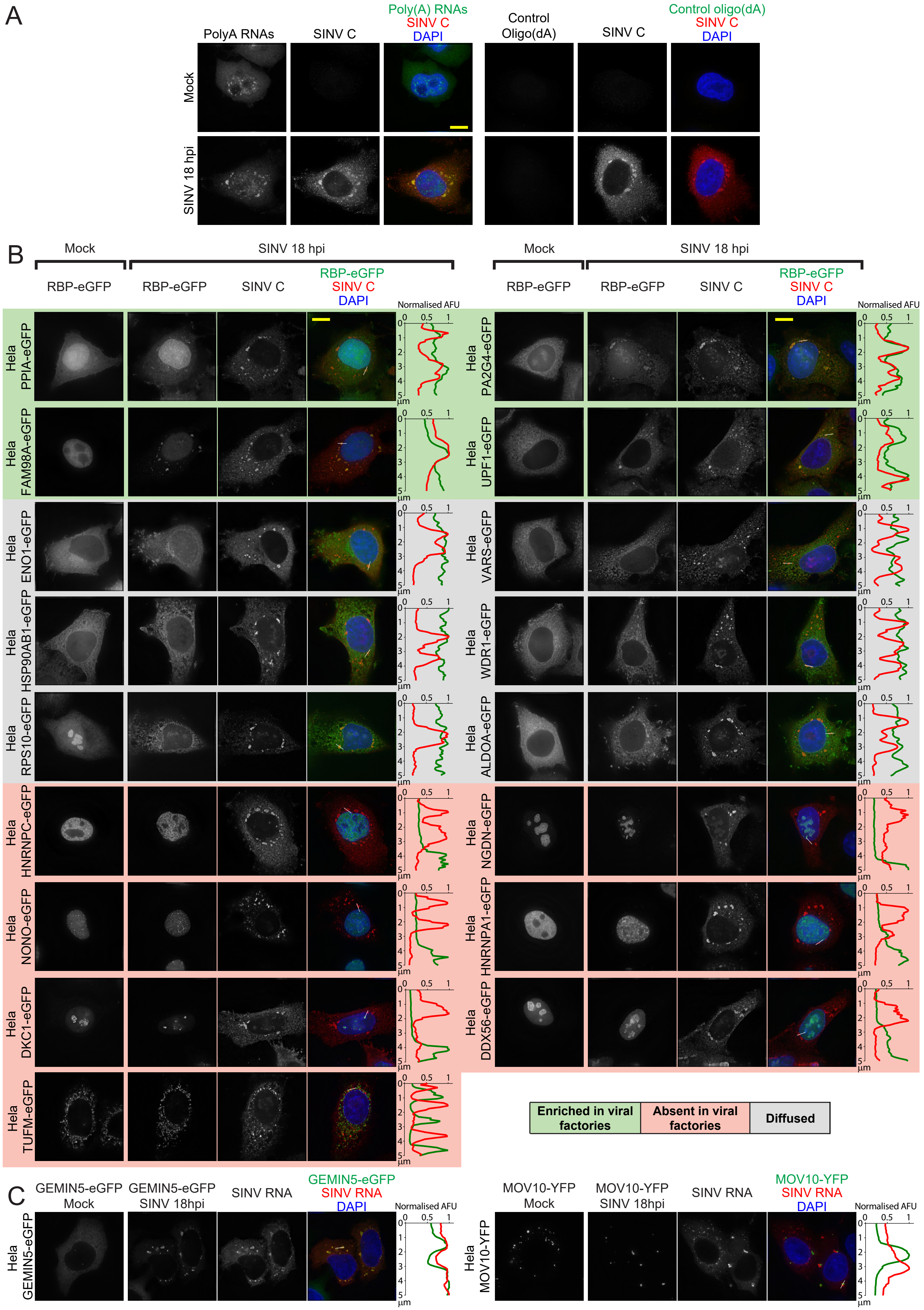

Figure S4 (Garcia-Moreno et al)

Figure S4. Localisation of host RBPs in SINV-infected HeLa cells; related to Figure 4. A) Localisation analysis of poly(A) RNAs and SINV C protein in uninfected and infected cells (18 hpi) by combined fluorescence *in situ* hybridisation with an oligo(dT) probe coupled to Alexa594 and immunofluorescence with an antibody against SINV C. DAPI was used to indicate the position of the nuclei; oligo(dA) probe coupled to Alexa 594 was used as a negative control. At 18 hpi, cytoplasmic poly(A) RNA concentrates in cytoplasmic foci that co-localise with SINV C. This poly(A) RNA is thus likely to be viral. B) Localisation by immunofluorescence of the eGFP-fused RBPs in uninfected or SINV-infected cells (18 hpi). Immunofluorescence with an antibody against SINV C and DAPI staining were used to localise the viral replication factories and the nuclei, respectively. At least 10 fields from at least two biological replicates were inspected for each protein to determine whether the proteins accumulate in the viral factories, are diffused in the cytoplasm or are excluded from the viral factories (indicated with a green, grey or red box, respectively). Plots of green and red fluorescence intensity profiles in a representative 5 µm section (white line) are shown for each protein. AFU, arbitrary fluorescence units. C) Localisation analysis of SINV RNA and GEMIN5-GFP (left panel) or MOV10-YFP (right panel) in uninfected or SINV-infected cells (18 hpi) by combined *in situ* hybridisation and immunofluorescence. Yellow scale-bar represents 10 µm.

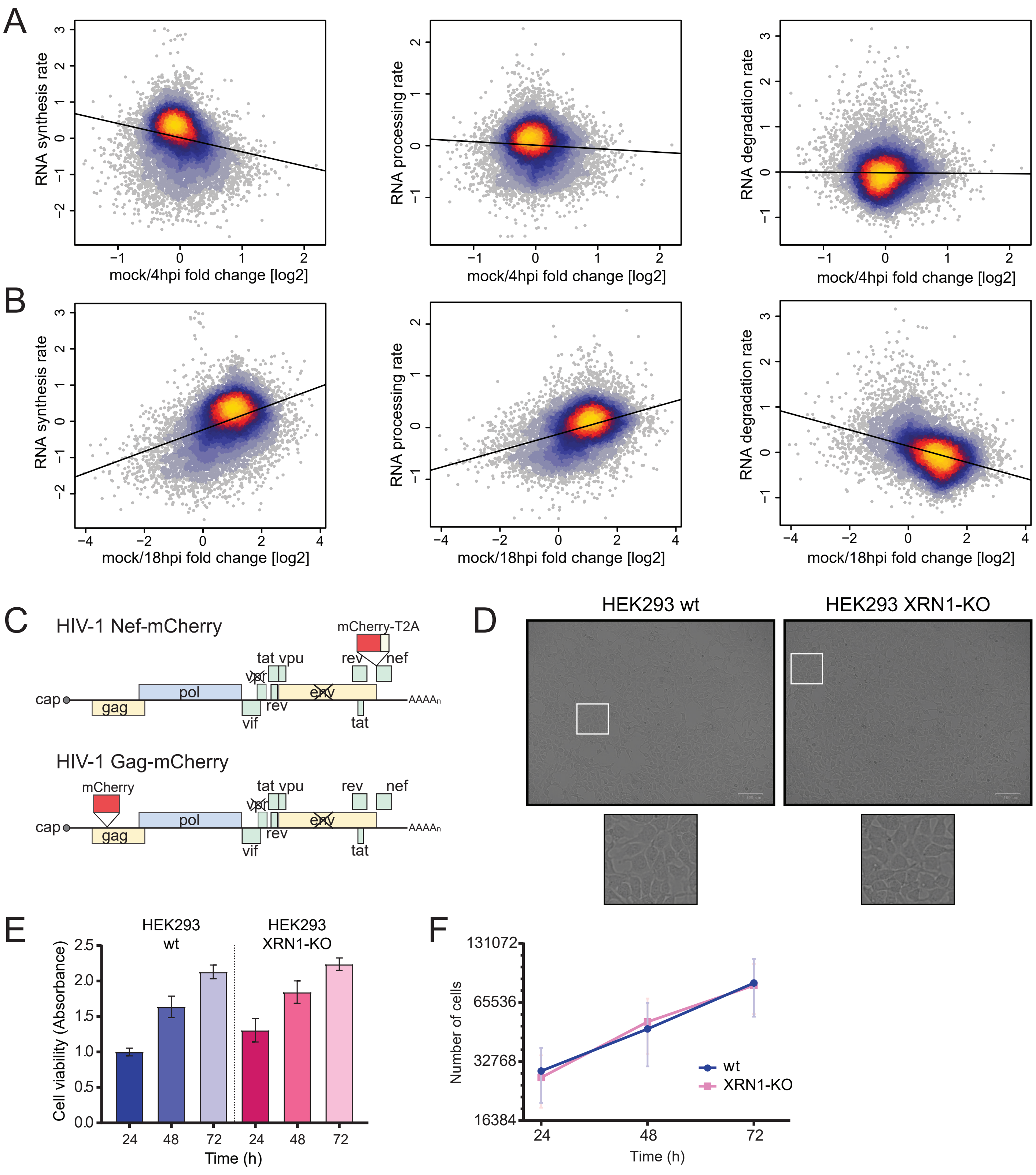

Figure S5 (Garcia-Moreno et al)

Figure S5. Analysis of the contribution of transcription, processing and degradation to the transcriptome of SINV-infected cells; related to Figure 5. A) Plots representing the log2 fold change of cellular RNAs detected by RNAseq between uninfected and SINV-infected (4 hpi) cells, compared to rates of RNA synthesis (left), processing (middle) and degradation (right) determined in (Mukherjee et al., 2017). B) As in (A) but comparing uninfected cells and 18 hpi. C) Schematic representation of HIV-1 single-round replication virus tagged with mCherry in Nef or Gag. D) Bright-field microscopy images of HEK293 wt and XRN1 knock out cells. E-F) 25,000 HEK293 wt or XRN1 knock out cells were seeded per well of a 96-well plate. Cell viability was estimated 24, 48 and 72 h later by adding CellTiter 96 Aqueous One Solution reagent and measuring absorbance at 490 nm on a plate reader (panel E). The number of cells was counted 24, 48 and 72 h after seeding using an automated cell counter (panel F). Values are represented as the mean ± standard deviation of three independent experiments.

A

Target	Compound	Concentration	% viability	SD	p-value
	DMSO	0.1%	100.00	±4.46	
IRE1α	4μ8C	20 μM	78.69	±11.72	0.00056
		30 μM	83.72	±9.11	0.00062
		40 μM	79.29	±9.25	0.00012
HSP90AB1	Ganetespib	100 nM	98.53	±3.10	0.24259
HSP90AB1	Geldanamycin	100 nM	81.49	±8.50	0.00014
		250 nM	81.43	±10.51	0.00068
PPIA	CysA	5 μM	92.87	±6.07	0.00205
PA2G4	WS6	1 μM	100.01	±7.91	0.99804

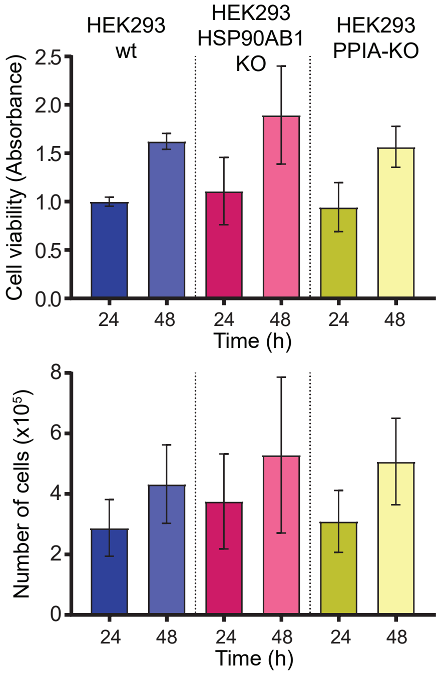

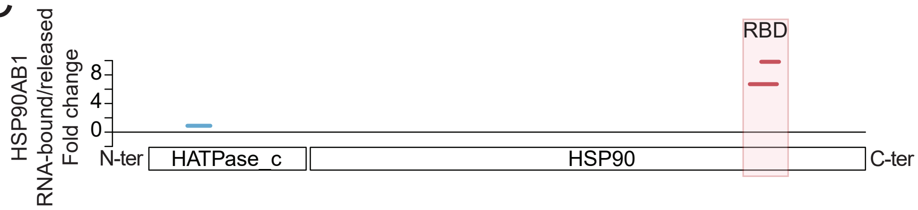

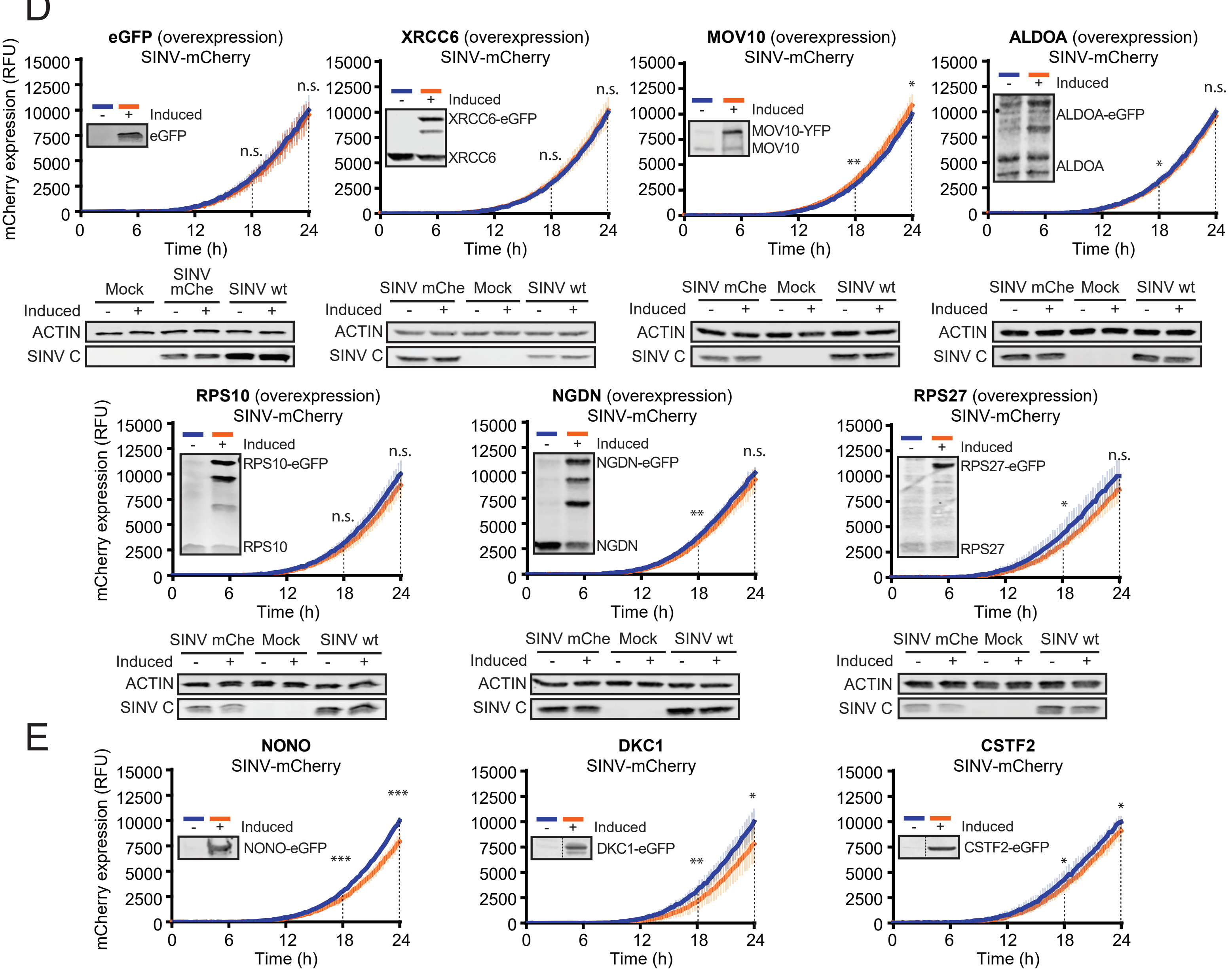

Figure S6 (Garcia-Moreno et al)

Figure S6. Effects of host RBP perturbation on SINV infection; related to Figure 6. A) HEK293 cells were treated with different inhibitors targeting host RBPs and viability was monitored using CellTiter 96 Aqueous One Solution kit. Absorbance at 490 nm was measured on a plate reader. Data is represented as the mean ± standard deviation (SD) of at least three independent experiments performed in triplicate. T test was employed to determine the significance of the changes in viability of cells treated with the inhibitors or dimethyl sulfoxide (DMSO) as a control. Any dose that caused a reduction in cell viability greater that 20% was considered 'toxic'. B) Cell viability (upper panel) and proliferation (lower panel) was analysed as in Figure S5E and S5F, respectively, in HEK293 HSP90AB1 or PPIA knock out cells. C) RNA-binding sites of HSP90AB1 identified by RBDmap (Castello et al., 2016). RBDmap employs UV crosslinking, denaturing lysis, oligo(dT) capture and partial proteolysis to determine in a system-wide manner the protein regions engaged in RNA binding. Y-axis indicates the enrichment of each identified peptide in the RNA-bound fraction and X-axis represents the protein from N- to C-terminus. Boxes below the X-axis indicate the position of annotated protein domains (Pfam). Red lines represent peptides engaged in RNA binding identified with 1% FDR (RBDpeps), while cyan lines indicate peptides that do not bind to RNA. D) Upper panels show the mCherry expression in HEK293 cells overexpressing the candidate RBPs fused to eGFP and infected with SINV-mCherry. Red fluorescence was measured as in Figure S1B. Overexpression was confirmed by western blot analyses with specific antibodies. RFU, relative fluorescence units. Bottom panels show Western blotting analysis of SINV C accumulation in cells overexpressing the candidate RBP-eGFP and infected with SINV or SINV-mCherry for 18 h. E) As in (D) but using eGFP antibody to detect the expression of RBP-eGFP fusion proteins. ***$p<0.001$; **$p<0.01$; *$p<0.05$; n.s. non-significant.

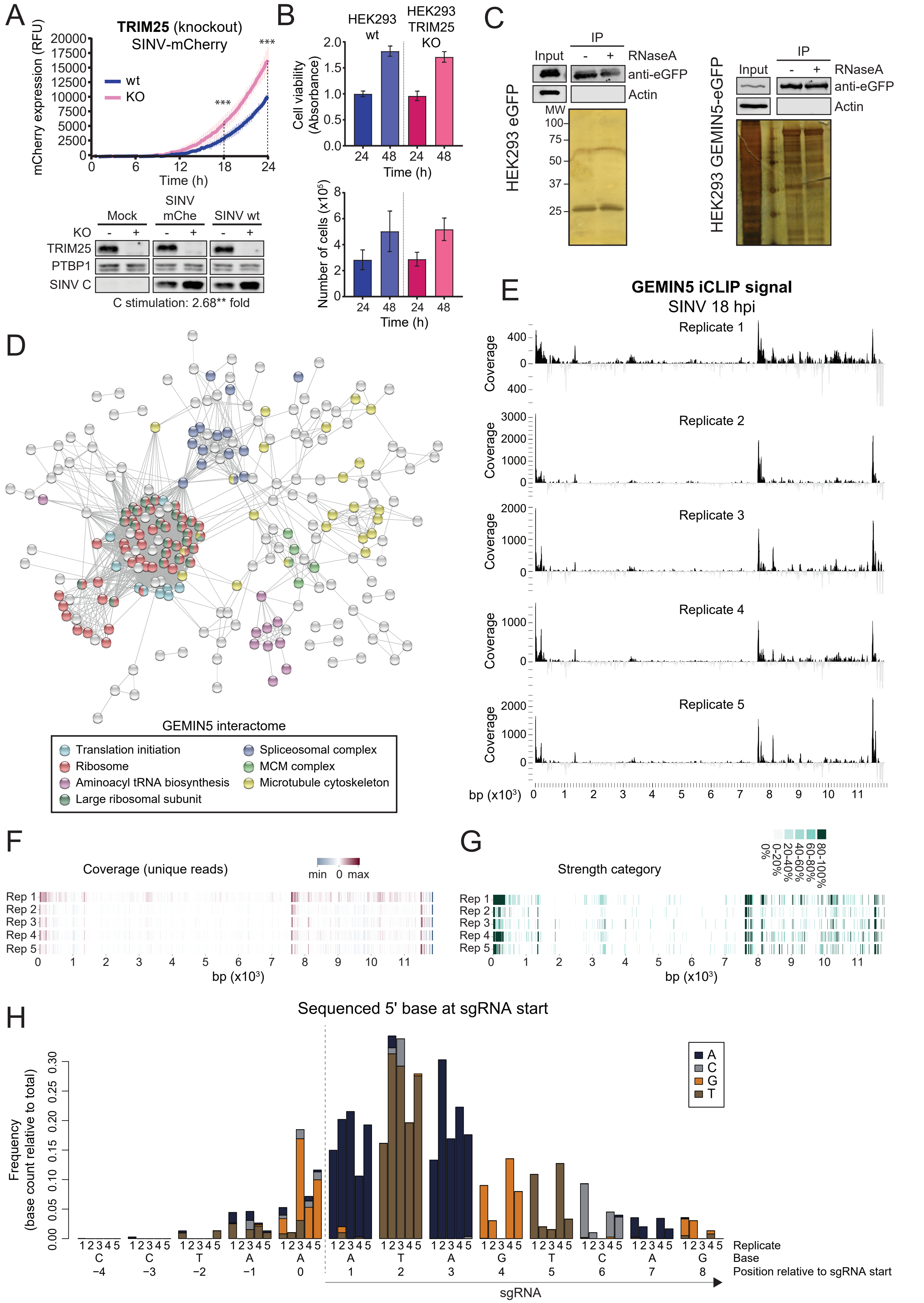

Figure S7 (Garcia-Moreno et al)

Figure S7. Analysis of GEMIN5 molecular partners in SINV-infected cells; related to Figure 7 and Table S5. A) mCherry expression in HEK293 wt and TRIM25 knock out cells infected with SINV-mCherry (upper panel). Red fluorescence was measured as in Figure S1B. RFU, relative fluorescence units. Bottom panels show Western blotting analysis of TRIM25 knock out cells infected with SINV-mCherry or SINV wt for 18 h. Fold change of SINV C (TRIM25 KO vs wt cells) is shown below. ***$p<0.001$; **$p<0.01$. B) TRIM25 knock out cells viability (upper panel) and proliferation (lower panel) was analysed as in Figure S5E and S5F, respectively. C) eGFP (left panels) and GEMIN5-eGFP (right panels) expressed in Tet-on HEK293 cells were immunoprecipitated with GFP-Trap_A. Eluates were analysed by Western blotting with an antibody against eGFP and silver staining. Immunoprecipitation was performed in the presence or absence of RNase A. D) STRING protein-protein interaction map of the proteins enriched in GEMIN5 IPs with $p<0.01$. E) Coverage of 5'-first base of unique sequenced fragments from GEMIN5 iCLIP shown along SINV RNA genome. The data is shown as 20 nt sliding mean of library-size normalised reads (RPM) after subtraction of background observed in GFP control. The five biological replicates are shown individually. F) As in E but in a heatmap representation. G) Binding sites predicted individually for the five replicates, grouped into five categories based on strength of binding (coverage normalised to peak width). H) Count of sequenced 5' base of unique molecules mapping to the region around SINV sgRNA start site relative to total count in the region, shown for the five replicates. Position indicated as ‘1’ is the first base of the SINV sgRNA. The number of counts (binding events) is highest at the region corresponding to the beginning of the sgRNA. Base ‘0’ just before the start of the sgRNA is often sequenced as ‘G’ rather than the genomic ‘A’. These observations suggest that GEMIN5 binding this region is likely to interact with the sgRNA, exclusively or in addition to the overlapping gRNA, and that that additional ‘G’ correspond to the cap structure.
