# Supplementary material for: System-wide Profiling of RNA-Binding Proteins Uncovers Key Regulators of Virus Infection
Source: Mol Cell. 2019 Apr 4;74(1):196–211.e11. doi: 10.1016/j.molcel.2019.01.017 (PMC6458987; doi:10.1016/j.molcel.2019.01.017)
Supplement: Document S2. Article plus Supplemental Information [file mmc8.pdf]

Resource

Molecular Cell

System-wide Profiling of RNA-Binding Proteins Uncovers Key Regulators of Virus Infection

Graphical Abstract

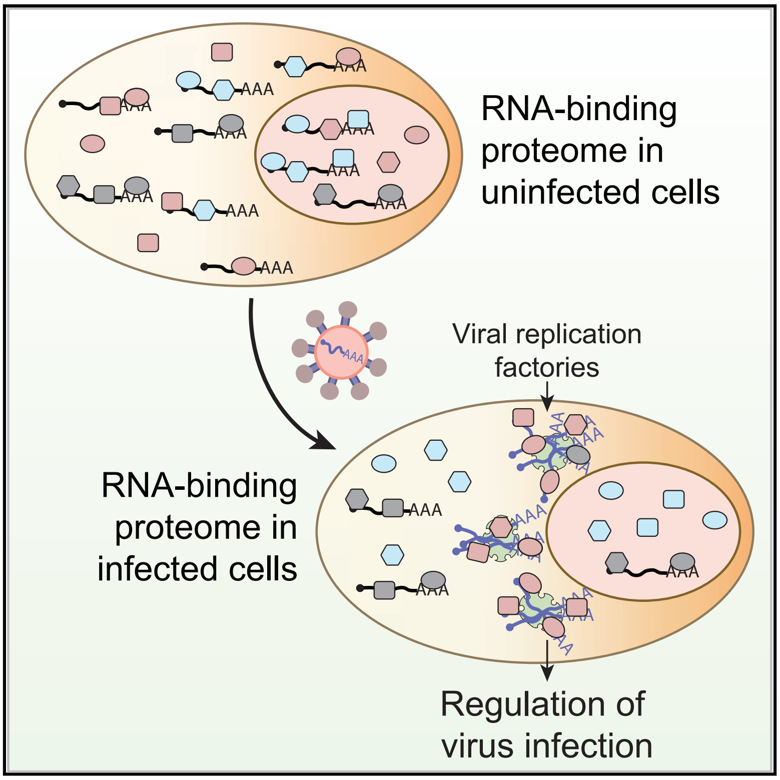

Authors

Manuel Garcia-Moreno, Marko Noerenberg, Shuai Ni, ..., Bernd Fischer, Shabaz Mohammed, Alfredo Castello

Correspondence

alfredo.castellopalomares@bioch.ox.ac.uk

In Brief

Garcia-Moreno, Noerenberg, Ni, and colleagues developed "comparative RNA-interactome capture" to analyze the RNA-bound proteome during virus infection. More than 200 cellular RNA-binding proteins change their binding activity in response to this challenge, mainly driven by transcript availability. Many of these RNA-binding proteins regulate viral replication and can be targeted to influence infection outcome.

Highlights

- A quarter of the RBPome changes upon SINV infection
- Alterations in RBP activity are largely explained by changes in RNA availability
- Altered RBPs are crucial for viral infection efficacy
- GEMIN5 binds to the 5′ end of SINV RNAs and regulates viral gene expression

Garcia-Moreno et al., 2019, Molecular Cell *74*, 196–211
April 4, 2019 © 2019 The Authors. Published by Elsevier Inc.
https://doi.org/10.1016/j.molcel.2019.01.017

Molecular Cell
Resource

System-wide Profiling of RNA-Binding Proteins Uncovers Key Regulators of Virus Infection

Manuel Garcia-Moreno,[1,12] Marko Noerenberg,[1,12] Shuai Ni,[3,4,12] Aino I. Järvelin,[1] Esther González-Almela,[5] Caroline E. Lenz,[1] Marcel Bach-Pages,[1] Victoria Cox,[1] Rosario Avolio,[1,6] Thomas Davis,[1] Svenja Hester,[1] Thibault J.M. Sohier,[7] Bingnan Li,[8] Gregory Heikel,[9,10] Gracjan Michlewski,[9,10,11] Miguel A. Sanz,[5] Luis Carrasco,[5] Emiliano P. Ricci,[7] Vicent Pelechano,[8] Ilan Davis,[1] Bernd Fischer,[3,13] Shabaz Mohammed,[1,2] and Alfredo Castello[1,14,*]
[1]Department of Biochemistry, University of Oxford, OX1 3QU Oxford, UK
[2]Department of Chemistry, Chemistry Research Laboratory, University of Oxford, Mansfield Road, Oxford OX1 3TA, UK
[3]German Cancer Research Center (DKFZ), 69120 Heidelberg, Germany
[4]Faculty of Biosciences, Heidelberg University, Heidelberg, Germany
[5]Centro de Biologia Molecular "Severo Ochoa," Universidad Autonoma de Madrid, 28049 Madrid, Spain
[6]Department of Molecular Medicine and Medical Biotechnology, University of Naples Federico II, Naples, Italy
[7]Université de Lyon, ENSL, UCBL, CNRS, INSERM, LBMC, 46 Allée d'Italie, 69007 Lyon, France
[8]SciLifeLab, Department of Microbiology, Tumor, and Cell Biology, Karolinska Institutet, 17165 Solna, Sweden
[9]Wellcome Centre for Cell Biology, University of Edinburgh, Michael Swann Building, Edinburgh EH9 3BF, UK
[10]Division of Infection and Pathway Medicine, University of Edinburgh, The Chancellor's Building, 49 Little France Crescent, Edinburgh EH16 4SB, UK
[11]Zhejiang University-University of Edinburgh Institute, Zhejiang University, 718 East Haizhou Road, Haining, Zhejiang 314400, People's Republic of China
[12]These authors contributed equally
[13]Deceased
[14]Lead Contact
*Correspondence: alfredo.castellopalomares@bioch.ox.ac.uk
https://doi.org/10.1016/j.molcel.2019.01.017

SUMMARY

The compendium of RNA-binding proteins (RBPs) has been greatly expanded by the development of RNA-interactome capture (RIC). However, it remained unknown if the complement of RBPs changes in response to environmental perturbations and whether these rearrangements are important. To answer these questions, we developed "comparative RIC" and applied it to cells challenged with an RNA virus called sindbis (SINV). Over 200 RBPs display differential interaction with RNA upon SINV infection. These alterations are mainly driven by the loss of cellular mRNAs and the emergence of viral RNA. RBPs stimulated by the infection redistribute to viral replication factories and regulate the capacity of the virus to infect. For example, ablation of XRN1 causes cells to be refractory to SINV, while GEMIN5 moonlights as a regulator of SINV gene expression. In summary, RNA availability controls RBP localization and function in SINV-infected cells.

INTRODUCTION

RNA-binding proteins (RBPs) assemble with RNA forming ribonucleoproteins (RNPs) that dictate RNA fate (Glisovic et al., 2008). Historically, most of the known RBPs were characterized by the presence of well-established RNA-binding domains (RBDs), which include the RNA recognition motif, K-homology domain, and others (Lunde et al., 2007). However, stepwise identification of unconventional RBPs evoked the existence of a broader universe of protein-RNA interactions than previously anticipated (Castello et al., 2015). Recently, a system-wide approach termed RNA-interactome capture (RIC) has greatly expanded the compendium of RBPs (RBPome) (Hentze et al., 2018). RIC employs UV crosslinking, oligo(dT) capture under denaturing conditions, and quantitative proteomics to identify the complement of proteins interacting with polyadenylated (poly(A)) RNA in living cells (Baltz et al., 2012; Castello et al., 2012). RIC uncovered hundreds of unconventional RBPs, several of which are now known to play crucial roles in cell biology (Hentze et al., 2018). Recent work has suggested that cells can adapt to physiological cues through discrete alterations in the RBPome (Perez-Perri et al., 2018; Sysoev et al., 2016). However, it remains unknown to what extent the RBPome can be remodeled, how RBP responses are triggered, and what are the biological consequences of this plasticity. For example, RIC reported changes in the composition of the RBPome during fruit fly embryo development that could be explained by matching alterations in protein abundance (Sysoev et al., 2016). However, several RBPs did not follow this trend, displaying protein-level independent changes in RNA binding and raising the question of whether physiological perturbations can induce such responsive behavior more widely. To address this possibility, we developed a "comparative RIC" (cRIC) approach to profile with high accuracy RBP dynamics in cells infected with sindbis virus (SINV) (Figures 1A and 1B).

Viruses have been fundamental for the discovery and characterization of important steps of cellular RNA metabolism such as

 © 2019 The Authors. Published by Elsevier Inc.
This is an open access article under the CC BY license (http://creativecommons.org/licenses/by/4.0/).

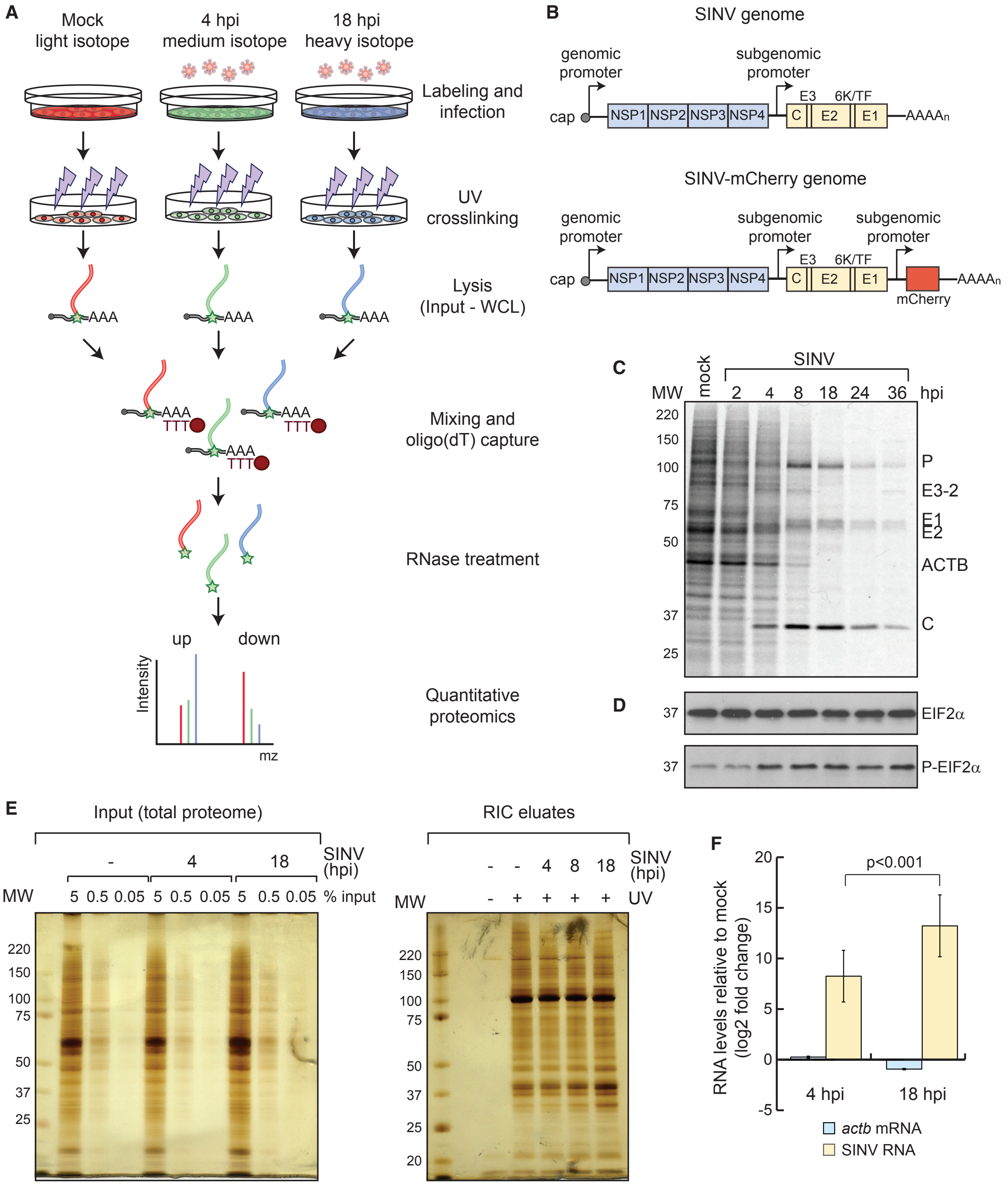

Figure 1. Application of RIC to HEK293 Cells Infected with SINV

(A) Schematic representation of cRIC.

(B) Schematic representation of SINV and chimeric SINV-mCherry genomes.

(C) Analysis of the proteins synthesized in uninfected and SINV-infected HEK293 cells by [^{35}S]-Met/Cys incorporation for 1 h followed by autoradiography.

(D) Analysis of total and phosphorylated eIF2α by western blotting.

(legend continued on next page)

RNA splicing, nuclear export, and translation initiation. This is due to their ability to hijack key cellular pathways by interfering with the activity of master regulatory proteins (Akusjarvi, 2008; Carrasco et al., 2018; Castelló et al., 2011; Garcia-Moreno et al., 2018; Lloyd, 2015). Furthermore, specialized RBPs are at the frontline of cellular antiviral defenses, detecting pathogen-associated molecular patterns (PAMPs) such as double-stranded RNA (dsRNA) or RNAs with 5′ triphosphate ends (Barbalat et al., 2011; Vladimer et al., 2014). Hence, virus infected cells represent an optimal scenario to assess the RBPome rearrangements.

Our data show that the complement of active cellular RBPs strongly changes in response to SINV infection, mainly due to deep variations in RNA availability. Importantly, "altered" RBPs are critical, as their perturbation affects viral fitness or/and the ability of the cell to counteract the infection. We envision that these RBPs represent novel targets for host-based antiviral therapies.

RESULTS AND DISCUSSION

Applying RIC to Cells Infected with SINV

To study the dynamics of cellular RBPs in response to physiological cues, we challenged cells with a cytoplasmic RNA virus and applied RIC. We chose SINV and HEK293 cells as viral and cellular models, respectively. SINV is a highly tractable virus that is transmitted from mosquito to vertebrates, causing high fever, arthralgia, malaise, and rash in humans. SINV replicates in the cytoplasm of the infected cell and produces three viral RNAs (Figures 1B and S1A): genomic RNA (gRNA), subgenomic RNA (sgRNA), and negative-stranded RNA. gRNA is packaged into the viral capsid and is translated to produce the nonstructural proteins (NSPs) that form the replication complex. The sgRNA is synthesized from an internal promoter and encodes the structural proteins (SPs), which are required to generate the viral particles. The negative strand serves as a template for replication. Both gRNA and sgRNA have cap and poly(A) tail.

HEK293 cells are an excellent cellular model to study SINV, as its infection exhibits all the expected molecular signatures, including (1) active viral replication (Figures 1C, S1B, and S1C), (2) host protein synthesis shutoff while viral proteins are massively produced (Figures 1C and S1B), (3) phosphorylation of the eukaryotic initiation factor 2 subunit alpha (EIF2α) (Figure 1D), and (4) formation of cytoplasmic foci enriched in viral RNA and proteins, commonly known as viral replication factories (Figures S1C and S1D). SINV infection causes a strong induction of the antiviral program, including β-interferon (β-IFN), which reflects the existence of active antiviral sensors and effectors (Figure S1E). Importantly, SINV achieves infection in a high proportion of cells (~85%) with relatively low number of viral particles (MOI) (Figure S1F), reducing cell-to-cell variability and biological noise.

Pilot RIC experiments in uninfected and SINV-infected cells revealed the isolation of a protein pool matching that previously observed for human RBPs (Castello et al., 2012), which strongly differed from the total proteome (Figure 1E). No proteins were detected in nonirradiated samples, demonstrating the UV dependency of RIC. Infection did not induce major alterations in the protein pattern observed by silver staining, which correspond to the most abundant housekeeping RBPs (Figure 1E). However, other less predominant bands displayed substantial differences, calling for in-depth proteomic analysis. Oligo(dT) capture led to the isolation of both host and SINV RNAs in infected cells (Figure 1F), which is expected as gRNA and sgRNA are polyadenylated.

SINV Infection Alters the Activity of Hundreds of RBPs

To allow accurate quantification of RBPs associated with poly(A) RNA under different physiological conditions, we developed a cRIC approach by combining the original protocol (Castello et al., 2013) with stable isotope labeling by amino acids in cell culture (SILAC) (Figure 1A). In brief, cells were grown in presence of light, medium, or heavy amino acids with incorporation efficiency >98%. Labeled cells were infected with SINV and irradiated with UV light at 4 and 18 h post-infection (hpi), using uninfected cells as a control (Figure 1A). These times correlate with key states in the SINV biological cycle; i.e., at 4 hpi, viral gene expression coexists with host protein synthesis, while the proteins synthesized at 18 hpi are almost exclusively viral (Figure 1C). SILAC labels were permutated among uninfected, 4 hpi, and 18 hpi in the three biological replicates to correct for possible isotope-dependent effects. After lysis, aliquots were stored for parallel transcriptomic and whole-proteome analyses. We combined equal amounts of the lysates from the three conditions prior to the oligo(dT) capture, and eluates were analyzed by quantitative proteomics (Figure 1A). Protein intensity ratios between condition pairs were computed, and the significance of each protein intensity change was estimated using a moderated t test (Figures 2A–2D, S2A, and S2B). We used a semiquantitative method for the cases in which an intensity value was missing ("zero") in one of the two conditions leading to "infinite" or zero ratios (Sysoev et al., 2016).

We identified a total of 794 proteins, 91% of which were already annotated by the Gene Ontology term "RNA-binding" or/and previously reported to be RBPs in eukaryotic cells by RIC (Hentze et al., 2018). Hence, the protein composition of our dataset largely resembles that of previously established RBPomes. Only 17 proteins displayed differential interaction with RNA at 4 hpi (Figures 2A, 2B, and S2A; Table S1). Fifteen of these were detected exclusively by the semiquantitative method due to the lack of intensity value in one condition, reflecting possible "on-off" and "off-on" states (Table S1). By contrast, 236 RBPs displayed altered RNA-binding activities at 18 hpi (Figures 2C, 2D, and S2B; Table S1). A total of 247 RBPs displayed

(E) Silver staining analysis of the "inputs" (i.e., total proteome, left) and eluates (i.e., RBPome, right) of a representative RIC experiment in SINV-infected cells.
(F) qRT-PCR analysis of the eluates of a representative RIC experiment using specific primers against SINV RNAs, *actb* and *gapdh* (for normalization) mRNAs. Error bars represent SE.
hpi, hours post-infection; MW, molecular weight.
See also Figure S1.

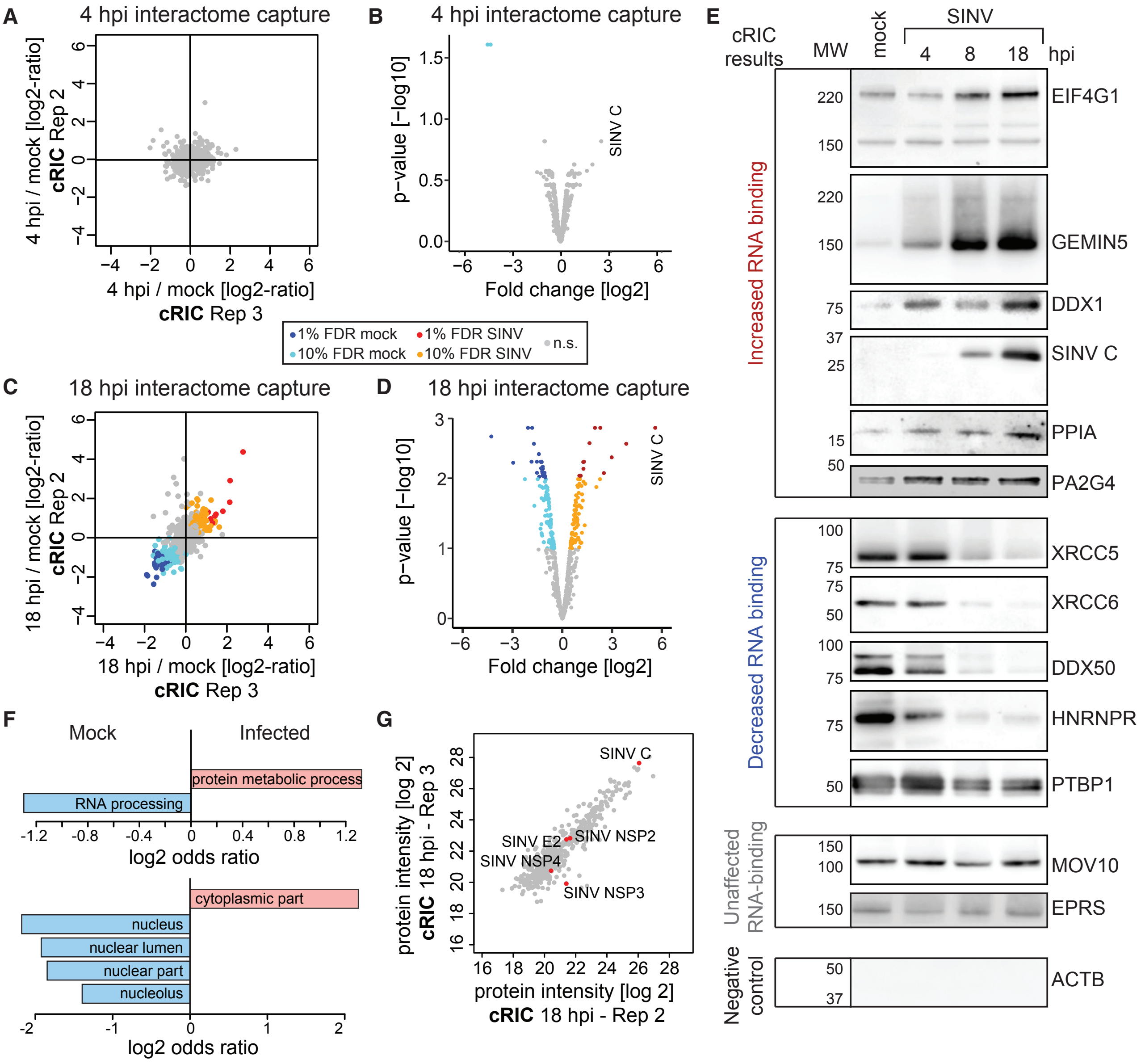

Figure 2. Analysis of the RNA-Bound Proteome in SINV-Infected HEK293 Cells by cRIC

(A) Scatterplot showing the intensity ratio between 4 hpi and uninfected conditions of each protein (dots) in the eluates of two biological replicates of cRIC.

(B) Volcano plot showing the log2 fold change and the significance (p value) of each protein between 4 hpi and uninfected conditions using data from three biological replicates.

(C) As in (A) but for 18 hpi.

(D) As in (B) but for 18 hpi.

(E) Western blotting analysis with specific antibodies of the eluates of a representative RIC experiment in SINV-infected HEK293 cells.

(F) Molecular function (top) and cellular component (bottom) Gene Ontology (GO) term enrichment analysis of the stimulated (salmon) against inhibited (blue) RBPs (18 hpi).

(G) Representative scatterplot comparing the raw intensity of each protein in the eluates of two cRIC replicates at 18 hpi.

FDR, false discovery rate; n.s., non-significant.

See also Figure S2 and Table S1.

differential binding in infected cells (4 and 18 hpi) and are referred to here as "altered RBPs." Interestingly, 181 of these lack classical RBDs, highlighting the importance of unconventional RBPs in virus infection.

To validate these results, we applied RIC to cells infected with SINV but, in this case, the eluates were analyzed by western blotting. We selected nine altered RBPs falling into three statistical categories; i.e., four with 1% false discovery rate (FDR), four

with 10% FDR, and one with nonsignificant changes. We included a positive control (the viral RBP SINV capsid [C]), two "non-altered" RBPs (MOV10 and EPRS), and a negative control (β-actin [ACTB]). Strikingly, the RNA-binding behavior of each protein fully matched the proteomic outcome, including those classified with 10% FDR (Figure 2E). Changes in RNA binding increased progressively throughout the infection. The proteomic data assigned a nonsignificant downregulation to HNRNPR (Table S1); however, the reduced activity of this protein was apparent by western blotting (Figure 2E), suggesting that our dataset may contain false negatives. Nonetheless, the excellent agreement between the proteomic and western blotting data supports the high quality of our results.

Determination of the RBP Networks Altered by SINV Infection

Among the 247 altered RBPs, 133 presented reduced and 114 increased association with RNA, and they are here referred to as "inhibited" and "stimulated" RBPs, respectively. Most of the inhibited RBPs were linked to nuclear processes such as RNA processing and export (Figures 2F and S2C). While cytoplasmic viruses are known to hamper nuclear RNA metabolism, the mechanisms by which this occurs remain poorly understood (Castelló et al., 2011; Gorchakov et al., 2005; Lloyd, 2015). Whether the inhibition of nuclear RBPs contributes to this phenomenon should be further investigated. Conversely, a large proportion of the stimulated RBPs are cytoplasmic and are linked to protein synthesis, 5′ to 3′ RNA degradation, RNA transport, protein metabolism, and antiviral response (Figures 2F and S2D).

Interestingly, several RBPs involved in translation were stimulated at 18 hpi despite the shutoff of host protein synthesis (Figure 1C), including 9 eukaryotic initiation factors, 3 elongation factors, and 12 ribosomal proteins. This enhancement is likely due to the high translational activity of SINV RNAs (Figure 1C) (Frolov and Schlesinger, 1996). The core components of the cap-binding complex EIF4A1 and EIF4E were not stimulated by the infection despite the activation of their protein partner, EIF4G1 (Table S1). In agreement, EIF4A1 and EIF4E do not participate in SINV sgRNA translation (Carrasco et al., 2018). A recent report showed that EIF3D is a cap-binding protein that controls the translation of specific mRNA pools (Lee et al., 2016). EIF3D is stimulated by SINV, and thus its potential contribution to SINV RNA translation deserves further consideration. Importantly, 88 altered RBPs associate with ribosomes in mouse cells (Table S2) (Simsek et al., 2017). The existence of "specialized ribosomes" has been proposed; however, experimental evidence is sparse (Au and Jan, 2014). Our results indicate that the composition of ribosomes and the scope of proteins associated with them may strongly differ between infected and uninfected cells, possibly resulting in differential translational properties.

cRIC uncovered 16 altered RNA helicases (Table S2), 13 of which were inhibited upon infection. RNA helicases are fundamental at virtually every stage of RNA metabolism (Chen and Shyu, 2014), and their inhibition is expected to have important consequences in RNA metabolism. Only 3 helicases were stimulated by SINV (DDX1, DHX57, and DHX29) (Figure 2E; Table S2). DHX29 enhances 48S complex formation on SINV sgRNA in reconstituted *in vitro* systems (Skabkin et al., 2010), and its stimulation supports its regulatory role in infected cells.

Notably, a defined subset of antiviral RBPs is stimulated upon SINV infection, including IFI16, IFIT5, TRIM25, TRIM56, and ZC3HAV1 (ZAP) (Table S1). IFI16 was previously described to bind dsDNA in cells infected with DNA viruses (Ni et al., 2016). Our data reveal that IFI16 also binds RNA, and it is activated early after SINV infection (4 hpi). This agrees with the recently described ability of IFI16 to restrict RNA virus infection (Thompson et al., 2014). These findings highlight the capacity of cRIC to identify antiviral factors responding virus infection.

Interestingly, cRIC also identified viral RBPs associated with poly(A) RNA, including the known viral RBPs (i.e., RNA helicase NSP2, the RNA polymerase NSP4, and capsid) and, unexpectedly, also NSP3 and E2 (Figures 2G and S2E). NSP3 was only quantified in two replicates (Figure S2E), and thus its interaction with RNA requires experimental confirmation. The identification of E2 in cRIC eluates was unexpected. In the viral particle of the related VEEV, E2 interacts with the capsid protein nearby cavities that communicate with the inner part of the virion where the gRNA density resides (Zhang et al., 2011), potentially enabling transitory or stochastic interactions with viral RNA.

RBP Responses to SINV Are Not Caused by Changes in Protein Abundance

Changes detected by cRIC can be a consequence of matching alterations in protein abundance (Sysoev et al., 2016). To assess this possibility globally, we analyzed the total proteome by quantitative proteomics (cRIC inputs; Figure 1A). Importantly, SINV infection did not cause noticeable changes in host RBP levels, including 129 RBPs with altered RNA-binding activity (Figures 3A–3C and S3A–S3C; Table S3). In agreement, silver and Coomassie staining did not show noticeable protein fluctuations except for the viral capsid (Figure 1E and 3D). The lack of changes in protein levels, even for altered RBPs, was confirmed by western blotting (Figure 3E; Table S3). It is not wholly unexpected that RBPs are unaffected in spite of the shutoff of cellular protein synthesis. Analogous to siRNA experiments, detectable decreases in protein abundance may require hours or even days after translational suppression, especially for relatively stable proteins.

The Transcriptome Undergoes Pervasive Changes in SINV-Infected Cells

Mechanistically, the activity of host RBPs can also be dictated by changes in the availability of their target RNAs. To test this possibility, we analyzed by RNA sequencing (RNA-seq) the total RNA isolated from cRIC input samples (Figure 1A). 4 h of SINV infection had a relatively minor impact on the host transcriptome (Figure 3F). By contrast, deep changes were observed at 18 hpi, with 12,372 differentially expressed RNAs ($p < 0.1$; Figures 3G and S3E–S3G). Only 1,448 RNAs were upregulated, and these were enriched in the Gene Ontology (GO) term "antiviral response." By contrast, 10,924 RNAs were downregulated, including many housekeeping genes (Table S4).

To validate these results by an orthogonal approach, we used qRT-PCR focusing on 20 mRNAs randomly chosen across the whole variation range. Importantly, data obtained

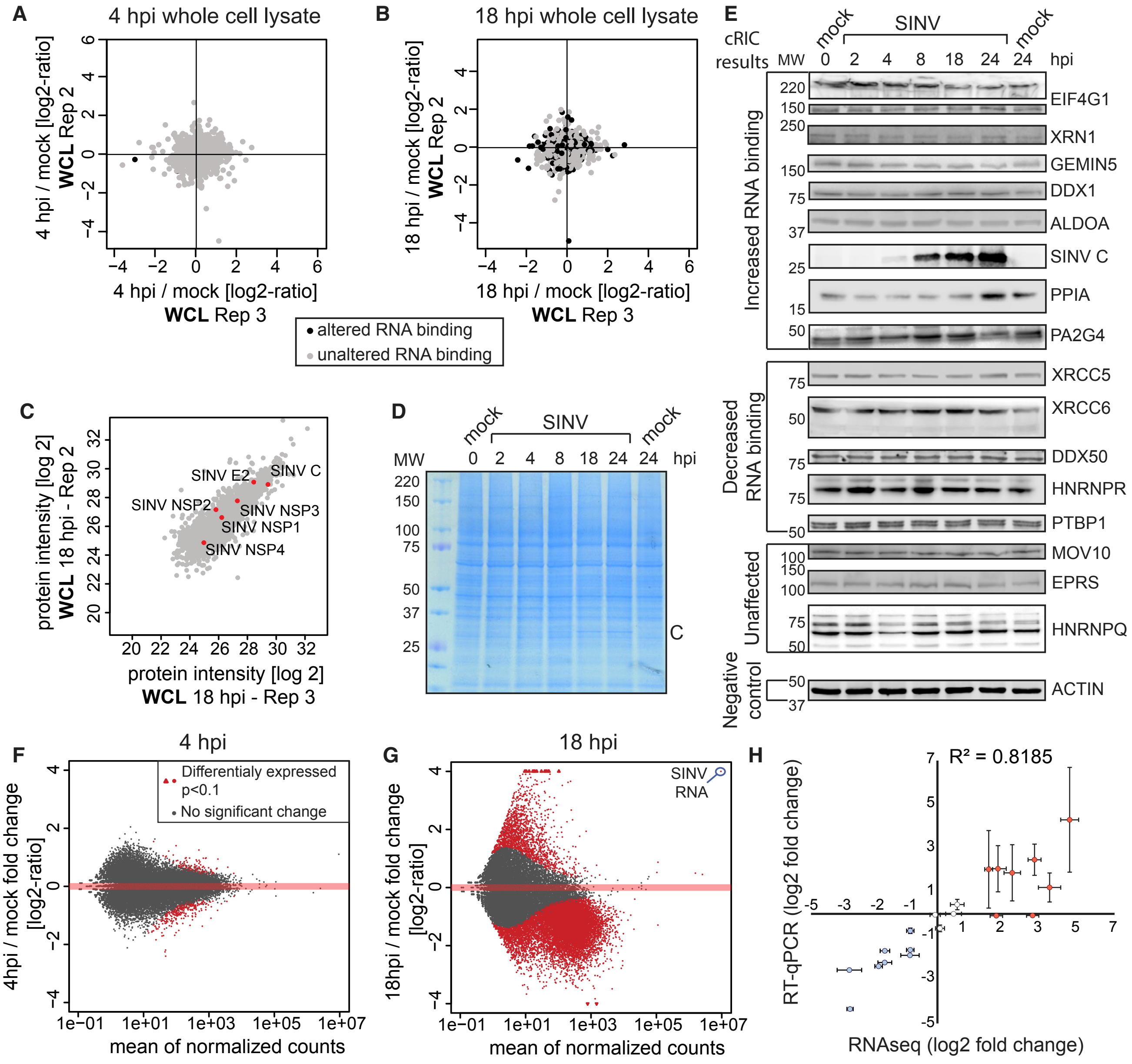

Figure 3. Proteomic and Transcriptomic Analyses of Whole SINV-Infected Cell Lysates

(A) Scatterplot comparing the intensity ratio between 4 hpi and uninfected conditions of each protein (dots) in the inputs (total proteome) of two biological replicates of cRIC. Black dots represent proteins significantly enriched in either 4 hpi or uninfected conditions in Figure 2A.

(B) As in (A) but for 18 hpi.

(C) Scatterplot comparing the intensity of each protein in the inputs of two cRIC replicates at 18 hpi.

(D) Representative Coomassie blue staining of cells infected with SINV.

(E) Western blotting analysis of lysates of cells infected with SINV (see Table S3 for quantification).

(F) MA plot comparing the read coverage and the log2 fold change between 4 hpi and uninfected cells of each gene detected in the RNA sequencing (RNA-seq) experiment. Red dots represent RNAs enriched with $p < 0.1$.

(G) As in (F) but for 18 hpi.

(H) Correlation of the RNA-seq and RT-qPCR data by plotting the log2 fold change for randomly selected transcripts by the two methods. Error bars represent SE of three independent experiments.

See also Figure S3 and Tables S3 and S4.

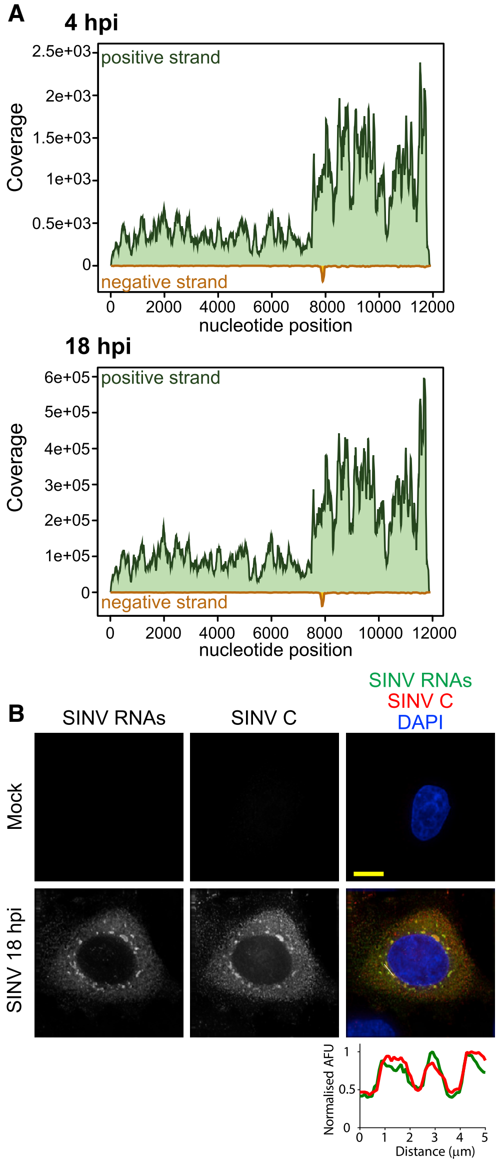

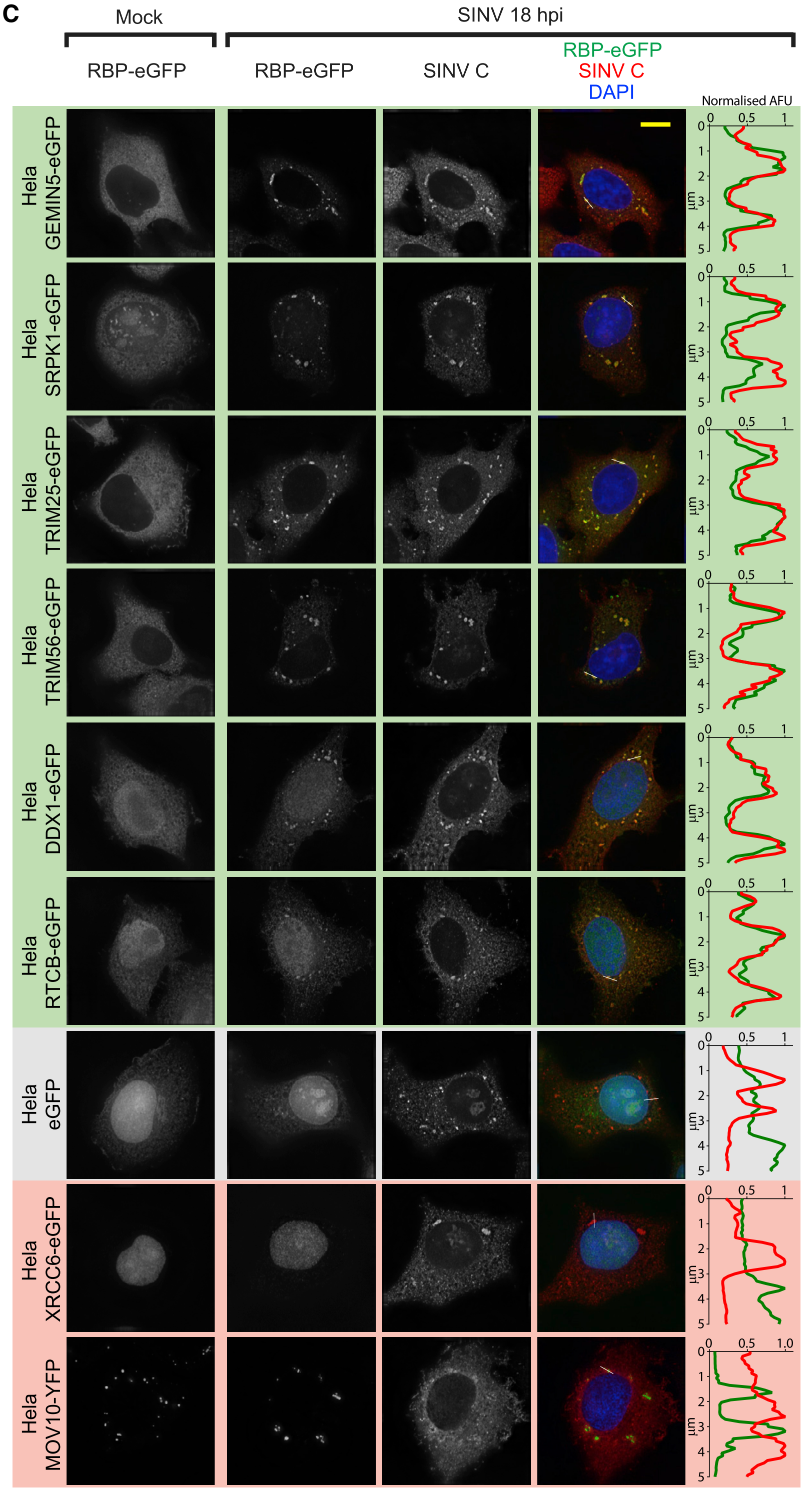

D

RBP localisation in SINV-infected cells

Enriched in viral factories	Absent in viral factories	Diffused
GEMIN5	XRCC6	GFP
SRPK1	MOV10	ENO1
TRIM25	HNRNPC	VARS
TRIM56	NGDN	HSP90AB1
DDX1	NONO	WDR1
RTCB	HNRNPA1	RPS10
UPF1	DKC1	ALDOA
PPIA	DDX56	
PA2G4	TUFM	
FAM98A		

Figure 4. Host RBP Localization in SINV-Infected Cells

(A) RNA-seq read coverage of the positive and negative RNA strand of SINV. Note that the y axes in both plots have different scales.

(B) Localization analysis of SINV RNA and capsid protein in infected HeLa cells at 18 hpi by combined *in situ* hybridization and immunofluorescence.

(legend continued on next page)

with both techniques strongly correlated (R^2 = 0.82) (Figure 3H), confirming the RNA-seq results. The decreased availability of cellular RNA could explain why 133 RBPs display reduced association with poly(A) RNA in infected cells (Table S1). In addition, inhibited RBPs could exchange poly(A) mRNA for non-poly(A) RNAs, which are not captured by the oligo(dT) beads.

Stimulated RBPs Are Relocated to the Viral Replication Factories

SINV produces two overlapping mRNAs, gRNA and sgRNA (Figures 1B and S1A), and, consequently, the read coverage was substantially higher in the last third of the gRNA, where both transcripts overlap (Figure 4A). Both sgRNA and gRNA have poly(A) and thus should contribute to the cRIC results (Figures 4A and S4A). Conversely, the negative strand has low abundance and lacks a poly(A) tail. Importantly, SINV RNAs become the most abundant RNA species, after rRNA, at 18 hpi (Figures 3G and S3G). The emergence of such abundant RNA substrates likely induces cellular RBPs to exchange the "declining" cellular mRNAs for "emerging" viral RNAs, driving the remodeling of the RBPome. Alternatively, "dormant" RBPs could be "awakened" by the recognition of signatures within the viral RNA, analogous to known antiviral RBPs (Vladimer et al., 2014). We thus hypothesized that RBPs displaying enhanced binding should colocalize with viral RNA.

SINV RNA and capsid accumulate in cytoplasmic foci that correspond to the viral factories (Figures S1D, 4B, and S4A). To test whether stimulated RBPs relocate to these foci, we generated 26 tetracycline-inducible cell lines expressing host RBPs fused to EGFP. These included 16 lines expressing stimulated RBPs and 8 expressing inhibited RBPs. The non-altered RBP, MOV10, and unfused EGFP were used as controls. Strikingly, 9 out of the 16 stimulated RBPs (56%) accumulated at viral factories demarcated by SINV C (Figures 4C, 4D, and S4B). Five additional stimulated RBPs (29%) showed diffuse localization in cytoplasm but were also present at the capsid-containing foci (Figure S4B). *In situ* hybridization analysis confirmed that SINV RNA co-localized with a representative stimulated RBP, GEMIN5, supporting the potential interplay between stimulated RBPs and viral RNA (Figure S4C). Among the stimulated RBPs, only NGDN, HNRNPA1 and the mitochondrial translation elongation factor TUFM (3 out of 16; 17%) were absent in the viral factories, which suggests that their function is restricted to host RNAs. HNRNPA1 was shown to bind SINV RNA (LaPointe et al., 2018; Lin et al., 2009), while in our analysis, it strictly displays nuclear localization (Figure S4B). We cannot rule out that a small pool of HNRNPA1 is present in the viral factories at undetectable levels or, alternatively, that the EGFP tag is affecting HNRNPA1 localization.

In contrast to stimulated RBPs, only one (out of 8; 12.5%) inhibited RBP was enriched in the viral factories (Figures 4D and S4B). This protein, called UPF1, is a helicase involved in the nonsense-mediated decay pathway and is known to inhibit infection of alphaviruses (Balistreri et al., 2014). Conversely, 5 out of 8 (62.5%) virus-inhibited RBPs are nuclear and remained nuclear after infection (Figures 4C, 4D, and S4B). These results indicate that, with exceptions, inhibited RBPs do not redistribute to the viral factories.

The Exonuclease XRN1 Is Essential for SINV Infection

The loss of cellular mRNAs is likely contributing to the remodeling of the RBPome by diminishing substrate availability. However, it is unclear how this phenomenon is triggered and whether it benefits or hampers viral infection. Changes in RNA levels can globally be a consequence of reduced transcription and/or increased RNA degradation. To explore which of these pathways contribute the most to RNA loss in SINV-infected cells, we compared the fold change of each mRNA in our dataset to the rate of synthesis, processing, and degradation of each individual transcript (Mukherjee et al., 2017). Transcription could explain most of the differences at 4 hpi, whereas RNA degradation accounted for more than 50% of the explained variance at 18 hpi (Figures 5A and S5A). We reasoned that this phenomenon can be a combined effect of the activation of the 5′ to 3′ RNA degradation machinery, as the exonuclease XRN1 and its interactor, PATL1, are stimulated at 18 hpi (Table S1), and a reduced transcriptional activity (Gorchakov et al., 2005).

XRN1 is broadly considered as an antiviral factor that erases viral RNA (Molleston and Cherry, 2017). RNA pseudoknots present in several viral RNAs are able to stall XRN1, leading to the production of sgRNAs (Chapman et al., 2014; Pijlman et al., 2008). In dengue virus (DENV), XRN1-derived sgRNAs can benefit infection by interfering with the antiviral response (Manokaran et al., 2015).

In SINV-infected cells, XRN1 and MOV10 foci (corresponding to P-bodies) are juxtaposed to the viral replication factories, suggesting that the exonuclease could attack viral RNA (Figures 4C, S4C, and 5B). To our surprise, XRN1 knockout (KO) cells were refractory to SINV infection, while partial KO led to an intermediate phenotype (Figure 5C). These results suggest that XRN1 activity is instead essential for SINV infection. XRN1 KO cells did not exhibit any defect in cell morphology, proliferation rate, or viability, and they supported efficiently the replication of HIV-1 (Figures 5D and S5C–S5F). These results indicate that XRN1 KO lines are not metabolically deficient or subjected to a heavy stress incompatible with virus infection.

To determine if XRN1 activity involves the generation of RNA degradation products, we analyzed our RNA-seq data. However, we did not found any increase in read coverage compatible with XRN1-derived degradation products, suggesting that XRN1 role in SINV infection differs from that described for DENV.

(C) Localization by immunofluorescence of the EGFP-fused RBPs and SINV C. Green and red fluorescence intensity profiles in a representative 5-μm section (white line) are plotted in (B) and (C).

(D) Summary of the observed localization of the 26 proteins tested in (C) and Figure S4B.

Scale bars represent 10 μm. AFU, arbitrary fluorescence units.

See also Figure S4.

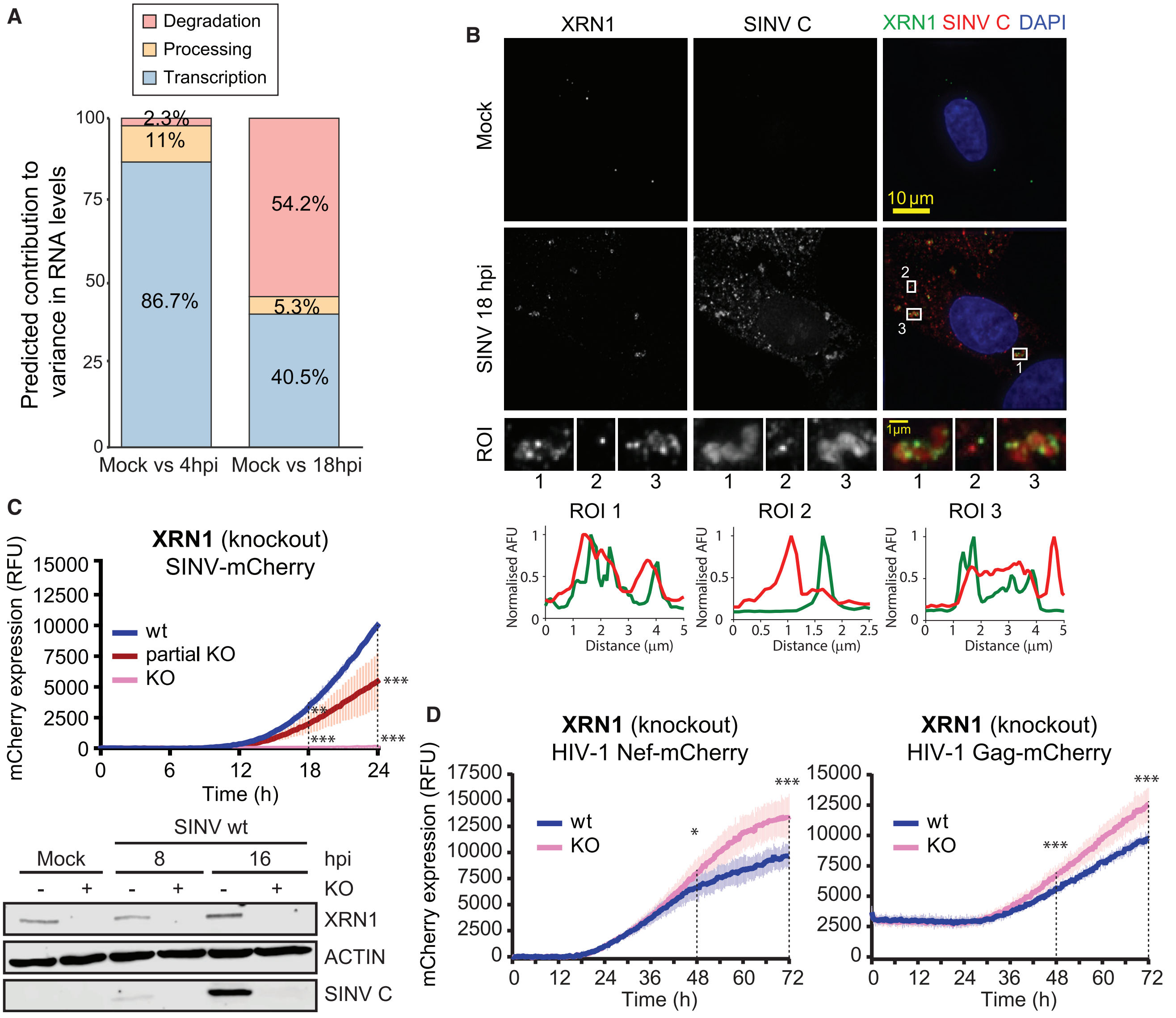

Figure 5. The Exonuclease XRN1 in Cells Infected with SINV

(A) Contribution of transcription, processing, and degradation to the transcriptomic changes induced by SINV. We compared our RNA-seq data to available data estimating these parameters (Mukherjee et al., 2017). ANOVA was used to predict the contribution of each RNA biological process to the variance in RNA levels.

(B) Immunolocalization of XRN1 and SINV C. Green and red fluorescence profiles for regions of interest (ROI) are displayed.

(C) Top: mCherry fluorescence in XRN1 KO and control cells infected with SINV-mCherry measured every 15 min in a plate reader with atmospheric control (5% CO2 and 37°C). RFU, relative fluorescence units. Western blot of XRN1 and SINV C (bottom).

(D) Infection fitness of HIV-$1_{\text{Nef-mCherry}}$ and HIV-$1_{\text{Gag-mCherry}}$ pseudotyped viruses in XRN1 KO cells. mCherry expression was measured as in (C).

mCherry fluorescence is represented as mean ± SD of three independent infections in each of the three biological replicates (n = 9). ***$p < 0.001$; **$p < 0.01$; *$p < 0.05$.

See also Figure S5.

RBPome Responses Are Biologically Important

To determine to a broader extent whether RBP responses are functionally important, we sought to study the impact of altered RBPs on virus infection. The ligase RTCB, together with DDX1, FAM98A, and other RBPs, forms the tRNA ligase complex (TRLC) (Popow et al., 2011). RTCB and DDX1 were stimulated by SINV (Table S1), and these and FAM98A accumulated in the viral factories (Figures 4C and S4B). TRLC mediates the unusual ligation of 3′-phosphate or 2′,3′-cyclic phosphate to a 5′-hydroxyl and these molecule ends are generated by a limited repertoire of cellular endonucleases, which include the endoplasmic reticulum resident protein IRE1α (Popow et al., 2011). SINV has been proposed to cause unfolded protein response (Rathore et al., 2013), which is compatible with the activation of IRE1α and TRLC in infected cells (Jurkin et al., 2014). Notably, inhibition of IRE1α with 4μ8C strongly reduced viral fitness in low,

non-cytotoxic concentrations (Figures 6A and S6A), suggesting that IRE1α and TRLC are positively contributing to SINV infection.

PPIA (also cyclophilin A) has also been classified as an RBP by RIC studies (Hentze et al., 2018). It switches proline conformation-modulating protein activity, which plays a crucial role in hepatitis C virus infection (Rupp and Bartenschlager, 2014). PPIA is also important for the infection of other viruses, such as HIV-1 (Li et al., 2007). PPIA RNA-binding activity is stimulated by SINV infection and is recruited to the viral factories (Figures 2E and S4B). Interestingly, SINV-mCherry infection is delayed by PPIA loss of function (KO and inhibition; Figures 6B, S6A, and S6B). Overexpression had no effect in SINV-mCherry fitness (Figure 6B, bottom).

The heat shock chaperone HSP90AB1 is stimulated by SINV (Table S1). HSP90AB1 has been classified as an RBP by RIC (Hentze et al., 2018), and its RBD has been located in a discrete region at its C-terminal domain (Figure S6C) (Castello et al., 2016). Chaperones from the HSP90 family are important in the remodeling of RNPs and are linked to virus infection (Geller et al., 2012; Iwasaki et al., 2010). Notably, SINV-mCherry infection was significantly delayed in HSP90AB1 KO cells, even though four homologs of this protein exist (Figures 6C and S6B). Moreover, the pro-viral activity of HSP90AB1 was confirmed by treatment with specific inhibitors (Figures 6C and S6A). Again, overexpression had no effect in SINV-mCherry fitness (Figure 6C). The implication of PPIA and HSP90 in the biological cycle of a variety of unrelated viruses highlights these proteins as master regulators of infection (Garcia-Moreno et al., 2018).

PA2G4 RNA-binding activity was also enhanced by SINV (Table S1). It associates with ribosomes (Table S2) (Simsek et al., 2017) and regulates the cap-independent translation of foot-and-mouth disease virus (FMDV) RNA (Monie et al., 2007). Treatment with its specific inhibitor WS6 hampered SINV-mCherry fitness (Figures 6D and S6A), suggesting that this protein promotes SINV infection. Overexpression did not cause any effect, as with previous examples (Figure 6D). The possibility that PA2G4 contributes to the non-canonical, cap-dependent translation of SINV RNAs should be further investigated.

SRPK1 is a kinase that phosphorylates the RS repeats present in SR proteins, which are involved in alternative splicing regulation, RNA export, and stability (Howard and Sanford, 2015). SINV infection stimulates SRPK1 RNA-binding activity (Table S1) and causes its relocation to viral replication factories (Figure 4C). Inhibition of SRPK1 hampers SINV and HIV-1 infection (Fukuhara et al., 2006), and we show here that overexpression of SRPK1 enhances SINV fitness (Figure 6E). This suggests that SRPK1 positively contributes to SINV infection. Future work should determine if SRPK1 kinase activity is involved in infection, and if so, which proteins it phosphorylates.

We tested the effects of overexpression of nine additional stimulated or inhibited RBPs fused to EGFP (Figures S6D and S6E). Phenotypes in viral fitness ranged from nonexistent (ALDOA, XRCC6, RPS10, MOV10, NGDN, and CSTF2) to mild (RPS27, NONO, and DKC1). The lack of phenotypic effects in overexpression experiments does not rule out that the protein actually participates in SINV infection (see above). Nevertheless, RBPs whose overexpression affects infection fitness have potential as regulatory proteins.

The family of tripartite-motif-containing (TRIM) proteins comprises more than 75 members endowed with E3 ubiquitin ligase activity, and few of them have been classified as RBPs by RIC (Hentze et al., 2018). Notably, SINV infection enhanced TRIM25 and TRIM56 interaction with RNA (Table S1), correlating with their redistribution to viral replication factories (Figure 4C). TRIM25 was proposed to interact with DENV RNA (Manokaran et al., 2015); however, this analysis employed native immunoprecipitation (IP) that cannot distinguish between direct and indirect protein-RNA interactions. To test if TRIM25 interacts directly with SINV RNA, we immunoprecipitated under stringent conditions TRIM25-EGFP from SINV-infected cells irradiated with UV light. Co-precipitated RNA was analyzed by RT-PCR using specific primers against SINV RNA. A band with the expected size was detected in TRIM25-EGFP IPs, but not in the negative controls (Figure 7A), confirming that TRIM25 interacts with SINV RNA directly. TRIM25 interaction with RNA enhances its E3 ubiquitin ligase activity (Choudhury et al., 2017). TRIM25-EGFP overexpression inhibited SINV-mCherry infection (Figure 7B), which agrees with its ability to activate the key antiviral factors RIG-I and ZC3HAV1 through ubiquitination (Gack et al., 2007; Li et al., 2017). It is known that TRIM56 binds double-stranded DNA. However, it enhances the antiviral response in cells infected with both DNA and RNA viruses (Seo et al., 2018; Tsuchida et al., 2010). cRIC thus complements these results, revealing that TRIM56 interacts directly with RNA (Table S1). As with TRIM25, overexpression of TRIM56-EGFP reduced SINV fitness (Figure 7B), confirming its capacity to restrict the infection of the RNA virus, SINV.

Importantly, 160 out of the 247 altered RBPs lack previous connections to virus infection (Garcia-Moreno et al., 2018). Hence, our dataset likely contains numerous pro- and antiviral RBPs yet to be uncovered.

GEMIN5 Binds to the 5′ UTR of SINV RNAs and Regulates Viral Protein Expression

GEMIN5 is a member of the survival motor neuron (SMN) complex, which mediates the assembly of the small nuclear RNPs (snRNPs) (Gubitz et al., 2002). It is strongly stimulated by SINV infection and redistributed to the viral factories co-localizing with SINV RNA (Figures 2E, 4C, and S4C). To our surprise, none of the known molecular partners of GEMIN5 (i.e., GEMIN and SMN proteins) were stimulated by SINV (Table S1), implying a GEMIN5-specific response that agrees with the existence of a free pool of GEMIN5 (Battle et al., 2007). In SINV-infected cells, overexpression of GEMIN5-EGFP caused a moderate but significant delay of mCherry production and strongly inhibited capsid synthesis (Figure 7B). These results align well with the described role of GEMIN5 in translational control (Francisco-Velilla et al., 2018; Piñeiro et al., 2015).

Protein-protein interaction analysis of GEMIN5-EGFP revealed that, in our experimental settings, it interacts with the ribosome, especially with the 60S subunit (Figure 7C, pink

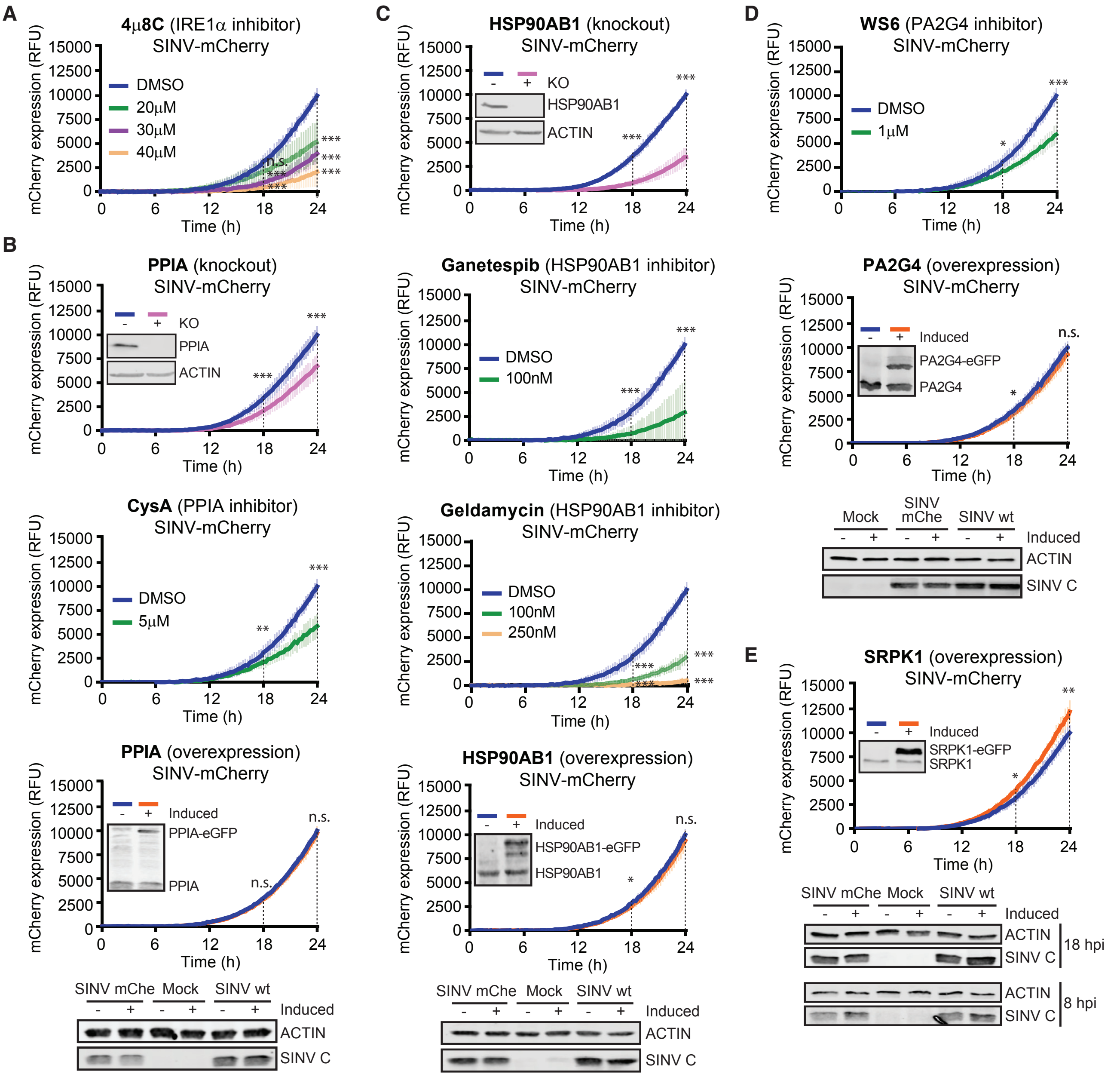

Figure 6. Impact of Stimulated RBPs in SINV Infection

(A) Expression of mCherry in HEK293 cells infected with SINV-mCherry and treated or not with the IRE1α inhibitor 4μ8C. Red fluorescence was measured as in Figure 5C.

(B) As in (A) but with PPIA KO cells (top), the PPIA inhibitor cyclosporine A (CysA) (middle), and cells overexpressing PPIA-EGFP (bottom). KO and overexpression of PPIA and SINV C accumulation (18 hpi) were assessed by western blotting.

(C) mCherry fluorescence in HSP90AB1 KO cells (top), cells treated with ganetespib or geldamycin (middle panels), or cells overexpressing HSP90AB1-EGFP (bottom) and infected with SINV-mCherry. KO and overexpression of HSP90AB1 and SINV C accumulation (18 hpi) were assessed by western blotting.

(D) As in (A) but using the PA2G4 inhibitor WS6 (top) and cells overexpressing PA2G4-eGFP (midde). Right: western blots against SINV C at 18 hpi.

(E) As in (A) but with cells overexpressing SRPK1 (top). Overexpression of SRPK1 was assessed by western blotting. Bottom: western blots of SINV C in these cells at 18 hpi.

mCherry fluorescence is shown as the mean ± SD of three independent infections in each of the three biological replicates (n = 9). ***$p < 0.001$; **$p < 0.01$; *$p < 0.05$. SINV-mChe, SINV-mCherry; n.s., non-significant.

See also Figure S6.

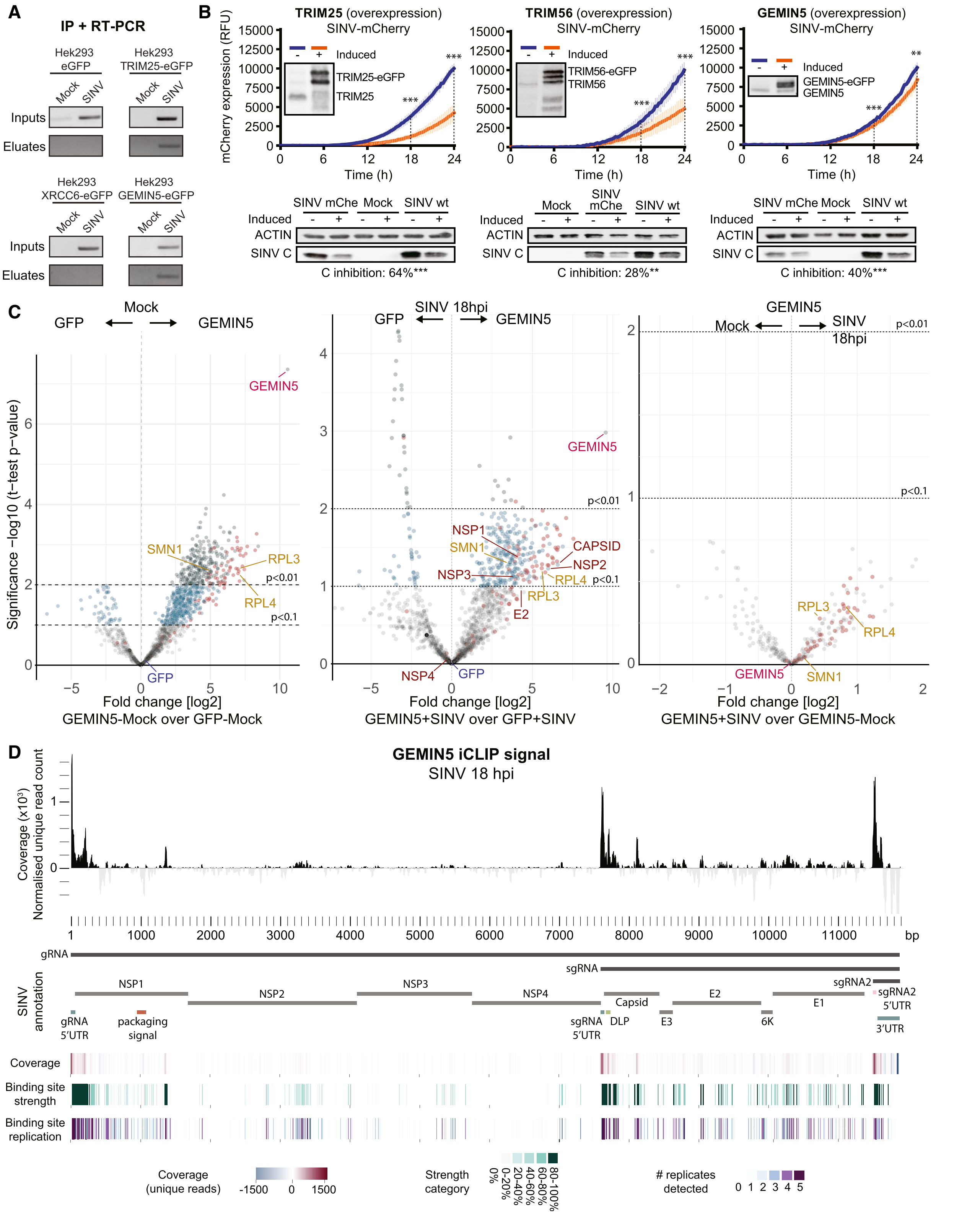

(legend on next page)

dots, left; Figures S7C and S7D; Table S5). This interaction is sustained in SINV-infected cells (Figure 7C, pink dots, middle and right). These results are in agreement with previous studies showing that GEMIN5 impacts protein synthesis at the translation elongation step through its direct interaction with the 60S ribosomal subunit and, in particular, with RPL3 and RPL4, which are also enriched in our IPs (Table S5) (Francisco-Velilla et al., 2016). We noticed that GEMIN5 is by far the most enriched protein in our IPs and that its Intensity Based Absolute Quantification (iBAQ) score is significantly higher than that of EGFP, suggesting that GEMIN5-EGFP interacts with the endogenous GEMIN5, likely forming oligomers, as previously described (Xu et al., 2016). Moreover, our data showed that GEMIN5 interacts with various viral proteins, chiefly with NSP1, NSP2, NSP3 and SINV C (Figure 7C, middle). The implications of these interactions in the modulation of GEMIN5 function deserve future considerations.

GEMIN5 is cleaved by the L protease of FMDV, and resulting C-terminal moiety enhances internal ribosome entry site (IRES)-driven translation (Piñeiro et al., 2013). However, GEMIN5 is not cleaved in SINV-infected cells (Figure 3E), and SINV RNAs lack an IRES and are capped (Carrasco et al., 2018). To test whether GEMIN5 binds SINV RNA, we performed an IP and RT-PCR analysis as outlined above. A PCR product was amplified in GEMIN5-EGFP eluates (Figure 7A), which agrees with the striking co-localization of SINV RNA and GEMIN5 (Figure S4C). To get insights into how GEMIN5 recognizes SINV RNAs, we employed single-nucleotide-resolution crosslinking and immunoprecipitation followed by sequencing (iCLIP) (König et al., 2010). Interestingly, the footprints with highest coverage mapped to the 5′ ends of the gRNA and sgRNA (Figures 7D and S7E–S7G). These reads often presented an additional guanosine at the 5′ end (Figure S7H), likely reflecting binding to the cap structure. These results support previous data showing that GEMIN5 is captured in cap-Sepharose beads (Bradrick and Gromeier, 2009). Additional peaks overlap with the downstream loop (DLP), which is a hairpin structure that stimulates the translation of the sgRNA (Frolov and Schlesinger, 1996). Interaction with the cap, 5′ UTR, and DLP of viral RNAs aligns well with the proposed role as translational regulator and the observed inhibition of capsid expression. Our data support the model in which GEMIN5 recognizes the 5′ end of the gRNA and sgRNA and prevents their translation by interfering with ribosomal function.

Outlook

We show here that SINV infection induces changes in the active RBPome that affects both well-established and unconventional RBPs. Mechanistically, the RBPome rearrangement can be explained by the loss of cellular RNA and the emergence of the highly abundant viral RNA. Supporting this conclusion, we observed that most of the RBPs with enhanced activity accumulate in the viral factories together with the viral RNA. However, this RNA-driven remodeling of the RBPome is not incompatible with complementary ‘‘fine-tuning’’ regulatory mechanisms affecting RBPs on an individual basis. For example, it is known that virus infection triggers signaling pathways involving kinases (Figure 1D), E3 ubiquitin ligases, prolyl *cis/trans* isomerases, and chaperones (Carrasco et al., 2018; Gack et al., 2007; Li et al., 2017). Here, we show that these protein families are represented among the stimulated RBPs, including SRPK1, TRIM25, TRIM56, PPIA, and HSP90AB1. Hence, it is plausible that post-translational control also contributes to RBP regulation in SINV-infected cells. Moreover, interactions with viral proteins can regulate RBP function (Fros et al., 2012). We show that GEMIN5 interacts with several viral proteins, suggesting that this regulatory mechanism may apply to altered RBPs more broadly (Figure 7C).

Importantly, changes in the RBPome are biologically important, as perturbation of the altered RBPs strongly affects SINV infection. Therefore, every protein reported here to respond to SINV infection has potential as anti- or pro-viral factor, highlighting cellular RBPs as promising targets for antiviral therapies.

Some of the outstanding questions derived from this work include whether the distinct composition of ribosomes in infected cells affects their translational properties, why the lack of the exonuclease XRN1 makes the cells refractory to SINV, what triggers the degradation of host RNA, and why the transcripts induced by the antiviral response are resistant to degradation. Moreover, GEMIN5 emerges as a highly responsive RBP that impairs SINV infection. The exact mechanisms underpinning GEMIN5 effects in translation require further investigation.

Figure 7. Effects of RBPs with Antiviral Potential in SINV Infection

(A) UV crosslinking and immunoprecipitation of TRIM25-EGFP, GEMIN5-EGFP, XRCC6-EGFP, or unfused EGFP in cells infected or not with SINV for 18 h. The presence of SINV RNA in eluates and inputs was detected by RT-PCR using specific primers against SINV RNAs.

(B) Relative mCherry fluorescence produced in cells overexpressing TRIM25-EGFP (top left), TRIM56-eGFP (top middle), GEMIN5-eGFP (top right), and infected with SINV-mCherry (measured as in Figure 5C). mCherry expression is represented as the mean ± SD of three independent infections in each of the three biological replicates (n = 9). Overexpression was assessed by western blotting. Bottom: western blots of SINV C at 18 hpi, indicating below the average inhibition of C relative to control cells. ***$p < 0.001$; **$p < 0.01$.

(C) Volcano plots comparing the intensity of proteins in GEMIN5-EGFP versus unfused EGFP IPs in uninfected (left) and infected cells (middle); every dot represents a protein. Dark green dots are proteins enriched with $p < 0.01$, blue dots are those enriched with $p < 0.1$, and gray dots represent nonenriched proteins. Pink dots represent ribosomal proteins. Right: a volcano plot comparing the intensity of proteins in GEMIN5 IPs in infected versus uninfected cells.

(D) iCLIP analysis of GEMIN5-binding sites on SINV RNA. Top: coverage pileup of 5′ first base of unique molecules mapping to the SINV genome, shown as 20-nt sliding mean of five replicates after GFP background subtraction. Each position is given relative to total SINV count (RPM). Middle: key features of SINV annotation. Bottom: the top track shows iCLIP coverage but as a heatmap representation. The middle heatmap shows GEMIN5 binding sites along SINV divided into five groups according to strength of binding. The bottom heatmap shows the number of replicates supporting each binding site when binding sites are called independently for each replicate.

See also Figure S7 and Table S5.

Finally, cRIC has been applied here to cells infected with SINV. However, it can now be extended to other viruses or physiological cues to improve our understanding of RBP regulation and its biological importance.

STAR★METHODS

Detailed methods are provided in the online version of this paper and include the following:

- KEY RESOURCES TABLE
- CONTACT FOR REAGENT AND RESOURCE SHARING
- EXPERIMENTAL MODEL AND SUBJECT DETAILS
  - Cell culture
  - Cell culture in SILAC media
  - Viruses
- METHOD DETAILS
  - RNA interactome capture
  - Conventional protein analyses
  - Reverse-transcription and quantitative PCR
  - Plasmids and recombinant DNA procedures
  - mCherry-based viral fitness assay
  - Drugs and cell viability assay
  - Protein-protein interactions analysis
  - RBP-RNA interaction analysis: CLIP/RT-PCR
  - Analysis of GEMIN5 binding sites by iCLIP
  - Immunofluorescence and RNA FISH assays
  - Determining the percentage of infected cells
  - Mass spectrometry
  - RNA sequencing
- QUANTIFICATION AND STATISTICAL ANALYSES
  - Proteomic quantitative analysis
  - RNA sequencing data analysis
  - Analysis of RNA synthesis, processing, and degradation
  - iCLIP-seq data processing
- DATA AND SOFTWARE AVAILABILITY

SUPPLEMENTAL INFORMATION

Supplemental Information can be found with this article online at https://doi.org/10.1016/j.molcel.2019.01.017.

ACKNOWLEDGMENTS

We dedicate this work to our colleagues and friends Bernd Fischer and Katrin Eichelbaum, who sadly passed away during the development of this work. We thank Matthias W. Hentze, Encarnacion Martinez-Salas, Javier Martinez, Kui Li, Quentin Sattentau, Ilan Davis, Richard Parton, and Jan Rehwinkel for reagents and advice. We thank the Oxford Micron Advanced Bioimaging Unit for support in microscopy experiments. A.C. is funded by MRC Career Development Award MR/L019434/1, MRC grant MR/R021562/1, and John Fell Funds from the University of Oxford. M.G.M. is funded by the European Union's Horizon 2020 research and innovation programme under Marie-Sklodowska-Curie grant agreement 700184. I.D. is funded by Wellcome Trust Investigator Award 209412/Z/17/Z. V.P. is funded by a SciLifeLab Fellowship, the Swedish Research Council (VR 2016-01842), a Wallenberg Academy Fellowship (KAW 2016.0123), and the Ragnar Söderberg Foundation. L.C. is funded by grant DGICYT SAF2015-66170-R (MINECO/FEDER). E.G.A. was awarded with a short-term EMBO fellowship (ASTF 358-2015) and an FPU fellowship FPU15/05709. G.M. was supported by the Wellcome Seed Award in Science (210144/Z/18/Z) and the Wellcome Trust Centre Core Grant (092076). G.H. was a recipient of a Wellcome Trust PhD studentship (105246/Z/14/Z).

AUTHOR CONTRIBUTIONS

Conceptualization, M.N., L.C., B.F., S.M., and A.C.; Methodology, M.G.-M., M.N., S.N., A.I.J., E.G.-A., C.E.L., I.D., B.F., S.M., and A.C.; Investigation, M.G.-M., M.N., S.N., A.I.J., E.G.-A., C.E.L., M.B.-P., V.C., R.A., T.D., S.H., T.J.M.S., B.L., M.A.S., E.P.R., V.P., B.F., S.M., and A.C.; Writing – Original Draft, M.G.-M., M.N., A.I.J., and A.C.; Writing – Editing, M.G.-M., M.N., S.N., A.I.J., C.E.L., S.M., and A.C.; Funding Acquisition, M.G.-M. and A.C.; Resources, G.H., G.M., L.C., E.P.R., V.P., I.D., B.F., S.M., and A.C.; Supervision, M.G.-M., M.N., A.I.J., B.F., S.M., and A.C.

DECLARATION OF INTERESTS

The authors declare no competing interests.

Received: June 18, 2018
Revised: December 18, 2018
Accepted: January 11, 2019
Published: February 21, 2019

REFERENCES

Akusjarvi, G. (2008). Temporal regulation of adenovirus major late alternative RNA splicing. Front. Biosci. *13*, 5006–5015.

Au, H.H., and Jan, E. (2014). Novel viral translation strategies. Wiley Interdiscip. Rev. RNA *5*, 779–801.

Balistreri, G., Horvath, P., Schweingruber, C., Zünd, D., McInerney, G., Merits, A., Mühlemann, O., Azzalin, C., and Helenius, A. (2014). The host nonsense-mediated mRNA decay pathway restricts mammalian RNA virus replication. Cell Host Microbe *16*, 403–411.

Baltz, A.G., Munschauer, M., Schwanhäusser, B., Vasile, A., Murakawa, Y., Schueler, M., Youngs, N., Penfold-Brown, D., Drew, K., Milek, M., et al. (2012). The mRNA-bound proteome and its global occupancy profile on protein-coding transcripts. Mol. Cell *46*, 674–690.

Barbalat, R., Ewald, S.E., Mouchess, M.L., and Barton, G.M. (2011). Nucleic acid recognition by the innate immune system. Annu. Rev. Immunol. *29*, 185–214.

Battle, D.J., Kasim, M., Wang, J., and Dreyfuss, G. (2007). SMN-independent subunits of the SMN complex. Identification of a small nuclear ribonucleoprotein assembly intermediate. J. Biol. Chem. *282*, 27953–27959.

Bradrick, S.S., and Gromeier, M. (2009). Identification of gemin5 as a novel 7-methylguanosine cap-binding protein. PLoS ONE *4*, e7030.

Carlson, M., and Pages, H. (2015). Homology information for Homo sapiens from Inparanoid. R package version 3.1.2 (Bioconductor).

Carrasco, L., Sanz, M.A., and González-Almela, E. (2018). The regulation of translation in alphavirus-infected cells. Viruses *10*, E70.

Castelló, A., Alvarez, E., and Carrasco, L. (2011). The multifaceted poliovirus 2A protease: regulation of gene expression by picornavirus proteases. J. Biomed. Biotechnol. *2011*, 369648.

Castello, A., Fischer, B., Eichelbaum, K., Horos, R., Beckmann, B.M., Strein, C., Davey, N.E., Humphreys, D.T., Preiss, T., Steinmetz, L.M., et al. (2012). Insights into RNA biology from an atlas of mammalian mRNA-binding proteins. Cell *149*, 1393–1406.

Castello, A., Horos, R., Strein, C., Fischer, B., Eichelbaum, K., Steinmetz, L.M., Krijgsveld, J., and Hentze, M.W. (2013). System-wide identification of RNA-binding proteins by interactome capture. Nat. Protoc. *8*, 491–500.

Castello, A., Hentze, M.W., and Preiss, T. (2015). Metabolic enzymes enjoying new partnerships as RNA-binding proteins. Trends Endocrinol. Metab. *26*, 746–757.

Castello, A., Fischer, B., Frese, C.K., Horos, R., Alleaume, A.M., Foehr, S., Curk, T., Krijgsveld, J., and Hentze, M.W. (2016). Comprehensive identification of RNA-binding domains in human cells. Mol. Cell *63*, 696–710.

Chapman, E.G., Costantino, D.A., Rabe, J.L., Moon, S.L., Wilusz, J., Nix, J.C., and Kieft, J.S. (2014). The structural basis of pathogenic subgenomic flavivirus RNA (sfRNA) production. Science *344*, 307–310.

Chen, C.Y., and Shyu, A.B. (2014). Emerging mechanisms of mRNP remodeling regulation. Wiley Interdiscip. Rev. RNA *5*, 713–722.

Choudhury, N.R., Heikel, G., Trubitsyna, M., Kubik, P., Nowak, J.S., Webb, S., Granneman, S., Spanos, C., Rappsilber, J., Castello, A., and Michlewski, G. (2017). RNA-binding activity of TRIM25 is mediated by its PRY/SPRY domain and is required for ubiquitination. BMC Biol. *15*, 105.

Cox, J., and Mann, M. (2008). MaxQuant enables high peptide identification rates, individualized p.p.b.-range mass accuracies and proteome-wide protein quantification. Nat. Biotechnol. *26*, 1367–1372.

Deutsch, E.W., Csordas, A., Sun, Z., Jarnuczak, A., Perez-Riverol, Y., Ternent, T., Campbell, D.S., Bernal-Llinares, M., Okuda, S., Kawano, S., et al. (2017). The ProteomeXchange consortium in 2017: supporting the cultural change in proteomics public data deposition. Nucleic Acids Res. *45* (D1), D1100–D1106.

Dobin, A., Davis, C.A., Schlesinger, F., Drenkow, J., Zaleski, C., Jha, S., Batut, P., Chaisson, M., and Gingeras, T.R. (2013). STAR: ultrafast universal RNA-seq aligner. Bioinformatics *29*, 15–21.

Durinck, S., Spellman, P.T., Birney, E., and Huber, W. (2009). Mapping identifiers for the integration of genomic datasets with the R/Bioconductor package biomaRt. Nat. Protoc. *4*, 1184–1191.

Francisco-Velilla, R., Fernandez-Chamorro, J., Ramajo, J., and Martinez-Salas, E. (2016). The RNA-binding protein Gemin5 binds directly to the ribosome and regulates global translation. Nucleic Acids Res. *44*, 8335–8351.

Francisco-Velilla, R., Fernandez-Chamorro, J., Dotu, I., and Martinez-Salas, E. (2018). The landscape of the non-canonical RNA-binding site of Gemin5 unveils a feedback loop counteracting the negative effect on translation. Nucleic Acids Res. *46*, 7339–7353.

Frolov, I., and Schlesinger, S. (1996). Translation of sindbis virus mRNA: analysis of sequences downstream of the initiating AUG codon that enhance translation. J. Virol. *70*, 1182–1190.

Fros, J.J., Domeradzka, N.E., Baggen, J., Geertsema, C., Flipse, J., Vlak, J.M., and Pijlman, G.P. (2012). Chikungunya virus nsP3 blocks stress granule assembly by recruitment of G3BP into cytoplasmic foci. J. Virol. *86*, 10873–10879.

Fukuhara, T., Hosoya, T., Shimizu, S., Sumi, K., Oshiro, T., Yoshinaka, Y., Suzuki, M., Yamamoto, N., Herzenberg, L.A., Herzenberg, L.A., and Hagiwara, M. (2006). Utilization of host SR protein kinases and RNA-splicing machinery during viral replication. Proc. Natl. Acad. Sci. USA *103*, 11329–11333.

Gack, M.U., Shin, Y.C., Joo, C.H., Urano, T., Liang, C., Sun, L., Takeuchi, O., Akira, S., Chen, Z., Inoue, S., and Jung, J.U. (2007). TRIM25 RING-finger E3 ubiquitin ligase is essential for RIG-I-mediated antiviral activity. Nature *446*, 916–920.

Garcia-Moreno, M., Järvelin, A.I., and Castello, A. (2018). Unconventional RNA-binding proteins step into the virus-host battlefront. Wiley Interdiscip. Rev. RNA *9*, e1498.

Geller, R., Taguwa, S., and Frydman, J. (2012). Broad action of Hsp90 as a host chaperone required for viral replication. Biochim. Biophys. Acta *1823*, 698–706.

Glisovic, T., Bachorik, J.L., Yong, J., and Dreyfuss, G. (2008). RNA-binding proteins and post-transcriptional gene regulation. FEBS Lett. *582*, 1977–1986.

Gorchakov, R., Frolova, E., and Frolov, I. (2005). Inhibition of transcription and translation in sindbis virus-infected cells. J. Virol. *79*, 9397–9409.

Gubitz, A.K., Mourelatos, Z., Abel, L., Rappsilber, J., Mann, M., and Dreyfuss, G. (2002). Gemin5, a novel WD repeat protein component of the SMN complex that binds Sm proteins. J. Biol. Chem. *277*, 5631–5636.

Hentze, M.W., Castello, A., Schwarzl, T., and Preiss, T. (2018). A brave new world of RNA-binding proteins. Nat. Rev. Mol. Cell Biol. *19*, 327–341.

Howard, J.M., and Sanford, J.R. (2015). The RNAissance family: SR proteins as multifaceted regulators of gene expression. Wiley Interdiscip. Rev. RNA *6*, 93–110.

Huppertz, I., Attig, J., D'Ambrogio, A., Easton, L.E., Sibley, C.R., Sugimoto, Y., Tajnik, M., König, J., and Ule, J. (2014). iCLIP: protein-RNA interactions at nucleotide resolution. Methods *65*, 274–287.

Iwasaki, S., Kobayashi, M., Yoda, M., Sakaguchi, Y., Katsuma, S., Suzuki, T., and Tomari, Y. (2010). Hsc70/Hsp90 chaperone machinery mediates ATP-dependent RISC loading of small RNA duplexes. Mol. Cell *39*, 292–299.

Jurkin, J., Henkel, T., Nielsen, A.F., Minnich, M., Popow, J., Kaufmann, T., Heindl, K., Hoffmann, T., Busslinger, M., and Martinez, J. (2014). The mammalian tRNA ligase complex mediates splicing of XBP1 mRNA and controls antibody secretion in plasma cells. EMBO J. *33*, 2922–2936.

Kolde, R. (2015). pheatmap: Pretty Heatmaps R package version 108.

König, J., Zarnack, K., Rot, G., Curk, T., Kayikci, M., Zupan, B., Turner, D.J., Luscombe, N.M., and Ule, J. (2010). iCLIP reveals the function of hnRNP particles in splicing at individual nucleotide resolution. Nat. Struct. Mol. Biol. *17*, 909–915.

LaPointe, A.T., Gebhart, N.N., Meller, M.E., Hardy, R.W., and Sokoloski, K.J. (2018). The identification and characterization of Sindbis virus RNA:host protein interactions. J. Virol. *92*, e02171-17.

Lee, A.S., Kranzusch, P.J., Doudna, J.A., and Cate, J.H. (2016). eIF3d is an mRNA cap-binding protein that is required for specialized translation initiation. Nature *536*, 96–99.

Li, J., Tang, S., Hewlett, I., and Yang, M. (2007). HIV-1 capsid protein and cyclophilin a as new targets for anti-AIDS therapeutic agents. Infect. Disord. Drug Targets *7*, 238–244.

Li, H., Handsaker, B., Wysoker, A., Fennell, T., Ruan, J., Homer, N., Marth, G., Abecasis, G., and Durbin, R.; 1000 Genome Project Data Processing Subgroup (2009). The Sequence Alignment/Map format and SAMtools. Bioinformatics *25*, 2078–2079.

Li, M.M., Lau, Z., Cheung, P., Aguilar, E.G., Schneider, W.M., Bozzacco, L., Molina, H., Buehler, E., Takaoka, A., Rice, C.M., et al. (2017). TRIM25 enhances the antiviral action of zinc-finger antiviral protein (ZAP). PLoS Pathog. *13*, e1006145.

Liao, Y., Smyth, G.K., and Shi, W. (2013). The Subread aligner: fast, accurate and scalable read mapping by seed-and-vote. Nucleic Acids Res. *41*, e108.

Lin, J.Y., Shih, S.R., Pan, M., Li, C., Lue, C.F., Stollar, V., and Li, M.L. (2009). hnRNP A1 interacts with the 5′ untranslated regions of enterovirus 71 and Sindbis virus RNA and is required for viral replication. J. Virol. *83*, 6106–6114.

Lloyd, R.E. (2015). Nuclear proteins hijacked by mammalian cytoplasmic plus strand RNA viruses. Virology *479-480*, 457–474.

Love, M.I., Huber, W., and Anders, S. (2014). Moderated estimation of fold change and dispersion for RNA-seq data with DESeq2. Genome Biol. *15*, 550.

Lunde, B.M., Moore, C., and Varani, G. (2007). RNA-binding proteins: modular design for efficient function. Nat. Rev. Mol. Cell Biol. *8*, 479–490.

Manokaran, G., Finol, E., Wang, C., Gunaratne, J., Bahl, J., Ong, E.Z., Tan, H.C., Sessions, O.M., Ward, A.M., Gubler, D.J., et al. (2015). Dengue subgenomic RNA binds TRIM25 to inhibit interferon expression for epidemiological fitness. Science *350*, 217–221.

Molleston, J.M., and Cherry, S. (2017). Attacked from all sides: RNA decay in antiviral defense. Viruses *9*, 2.

Monie, T.P., Perrin, A.J., Birtley, J.R., Sweeney, T.R., Karakasiliotis, I., Chaudhry, Y., Roberts, L.O., Matthews, S., Goodfellow, I.G., and Curry, S. (2007). Structural insights into the transcriptional and translational roles of Ebp1. EMBO J. *26*, 3936–3944.

Mukherjee, N., Calviello, L., Hirsekorn, A., de Pretis, S., Pelizzola, M., and Ohler, U. (2017). Integrative classification of human coding and noncoding genes through RNA metabolism profiles. Nat. Struct. Mol. Biol. *24*, 86–96.

Müller, B., Daecke, J., Fackler, O.T., Dittmar, M.T., Zentgraf, H., and Kräusslich, H.G. (2004). Construction and characterization of a fluorescently labeled infectious human immunodeficiency virus type 1 derivative. J. Virol. *78*, 10803–10813.

Ni, X., Ru, H., Ma, F., Zhao, L., Shaw, N., Feng, Y., Ding, W., Gong, W., Wang, Q., Ouyang, S., et al. (2016). New insights into the structural basis of DNA recognition by HINa and HINb domains of IFI16. J. Mol. Cell Biol. *8*, 51–61.

Perez-Perri, J.I., Rogell, B., Schwarzl, T., Stein, F., Zhou, Y., Rettel, M., Brosig, A., and Hentze, M.W. (2018). Discovery of RNA-binding proteins and characterization of their dynamic responses by enhanced RNA interactome capture. Nat. Commun. *9*, 4408.

Pfaffl, M.W. (2001). A new mathematical model for relative quantification in real-time RT-PCR. Nucleic Acids Res. *29*, e45.

Pijlman, G.P., Funk, A., Kondratieva, N., Leung, J., Torres, S., van der Aa, L., Liu, W.J., Palmenberg, A.C., Shi, P.Y., Hall, R.A., and Khromykh, A.A. (2008). A highly structured, nuclease-resistant, noncoding RNA produced by flaviviruses is required for pathogenicity. Cell Host Microbe *4*, 579–591.

Piñeiro, D., Fernández, N., Ramajo, J., and Martínez-Salas, E. (2013). Gemin5 promotes IRES interaction and translation control through its C-terminal region. Nucleic Acids Res. *41*, 1017–1028.

Piñeiro, D., Fernandez-Chamorro, J., Francisco-Velilla, R., and Martinez-Salas, E. (2015). Gemin5: a multitasking RNA-binding protein involved in translation control. Biomolecules *5*, 528–544.

Popow, J., Englert, M., Weitzer, S., Schleiffer, A., Mierzwa, B., Mechtler, K., Trowitzsch, S., Will, C.L., Lührmann, R., Söll, D., and Martinez, J. (2011). HSPC117 is the essential subunit of a human tRNA splicing ligase complex. Science *331*, 760–764.

Rathore, A.P., Ng, M.L., and Vasudevan, S.G. (2013). Differential unfolded protein response during Chikungunya and Sindbis virus infection: CHIKV nsP4 suppresses eIF2α phosphorylation. Virol. J. *10*, 36.

Rupp, D., and Bartenschlager, R. (2014). Targets for antiviral therapy of hepatitis C. Semin. Liver Dis. *34*, 9–21.

Sanz, M.A., and Carrasco, L. (2001). Sindbis virus variant with a deletion in the 6K gene shows defects in glycoprotein processing and trafficking: lack of complementation by a wild-type 6K gene in trans. J. Virol. *75*, 7778–7784.

Seo, G.J., Kim, C., Shin, W.J., Sklan, E.H., Eoh, H., and Jung, J.U. (2018). TRIM56-mediated monoubiquitination of cGAS for cytosolic DNA sensing. Nat. Commun. *9*, 613.

Sielaff, M., Kuharev, J., Bohn, T., Hahlbrock, J., Bopp, T., Tenzer, S., and Distler, U. (2017). Evaluation of FASP, SP3, and iST protocols for proteomic sample preparation in the low microgram range. J. Proteome Res. *16*, 4060–4072.

Simsek, D., Tiu, G.C., Flynn, R.A., Byeon, G.W., Leppek, K., Xu, A.F., Chang, H.Y., and Barna, M. (2017). The mammalian ribo-interactome reveals ribosome functional diversity and heterogeneity. Cell *169*, 1051–1065.e1018.

Skabkin, M.A., Skabkina, O.V., Dhote, V., Komar, A.A., Hellen, C.U., and Pestova, T.V. (2010). Activities of Ligatin and MCT-1/DENR in eukaryotic translation initiation and ribosomal recycling. Genes Dev. *24*, 1787–1801.

Smyth, G.K. (2004). Linear models and empirical Bayes methods for assessing differential expression in microarray experiments. Stat. Appl. Genet. Mol. Biol. *3*, Article3.

Sysoev, V.O., Fischer, B., Frese, C.K., Gupta, I., Krijgsveld, J., Hentze, M.W., Castello, A., and Ephrussi, A. (2016). Global changes of the RNA-bound proteome during the maternal-to-zygotic transition in Drosophila. Nat. Commun. *7*, 12128.

Szklarczyk, D., Morris, J.H., Cook, H., Kuhn, M., Wyder, S., Simonovic, M., Santos, A., Doncheva, N.T., Roth, A., Bork, P., et al. (2017). The STRING database in 2017: quality-controlled protein-protein association networks, made broadly accessible. Nucleic Acids Res. *45* (D1), D362–D368.

Thompson, M.R., Sharma, S., Atianand, M., Jensen, S.B., Carpenter, S., Knipe, D.M., Fitzgerald, K.A., and Kurt-Jones, E.A. (2014). Interferon γ-inducible protein (IFI) 16 transcriptionally regulates type i interferons and other interferon-stimulated genes and controls the interferon response to both DNA and RNA viruses. J. Biol. Chem. *289*, 23568–23581.

Tsuchida, T., Zou, J., Saitoh, T., Kumar, H., Abe, T., Matsuura, Y., Kawai, T., and Akira, S. (2010). The ubiquitin ligase TRIM56 regulates innate immune responses to intracellular double-stranded DNA. Immunity *33*, 765–776.

Tyanova, S., Temu, T., Sinitcyn, P., Carlson, A., Hein, M.Y., Geiger, T., Mann, M., and Cox, J. (2016). The Perseus computational platform for comprehensive analysis of (prote)omics data. Nat. Methods *13*, 731–740.

Vladimer, G.I., Górna, M.W., and Superti-Furga, G. (2014). IFITs: emerging roles as key anti-viral proteins. Front. Immunol. *5*, 94.

Wickham, H. (2009). ggplot2: Elegant Graphics for Data Analysis (Springer-Verlag).

Xu, C., Ishikawa, H., Izumikawa, K., Li, L., He, H., Nobe, Y., Yamauchi, Y., Shahjee, H.M., Wu, X.H., Yu, Y.T., et al. (2016). Structural insights into Gemin5-guided selection of pre-snRNAs for snRNP assembly. Genes Dev. *30*, 2376–2390.

Zhang, R., Hryc, C.F., Cong, Y., Liu, X., Jakana, J., Gorchakov, R., Baker, M.L., Weaver, S.C., and Chiu, W. (2011). 4.4 Å cryo-EM structure of an enveloped alphavirus Venezuelan equine encephalitis virus. EMBO J. *30*, 3854–3863.

STAR★METHODS

KEY RESOURCES TABLE

REAGENT or RESOURCE	SOURCE	IDENTIFIER
Antibodies		
anti-SINV C (304 and 306)	Laboratory of L. Carrasco	N/A
β-ACTIN	Sigma	Cat# A1978; RRID: AB_476692
ALDOA	Cusabio	Cat# PA00015A0Rb
DDX1	Bethyl	Cat# A300-521Q; RRID: AB_451046; Cat# A300-520; RRID: AB_451045
DDX50	Cusabio	Cat# PA861080LA01HU
EIF2α	Santa Cruz Biotechnology	Cat# sc-11386; RRID: AB_640075
Phospho-EIF2α (serine 51)	Cell Signaling Technology	Cat# 9721; RRID: AB_330951
EIF3G	Cusabio	Cat# PA03099A0Rb
EIF4G1 Nt - 981	Laboratory of L. Carrasco	N/A
EIF4G1 Ct - 987	Laboratory of L. Carrasco	N/A
ENO1	Cusabio	Cat# PA02395A0Rb
EPRS	Abcam	Cat# ab31531; RRID: AB_880047
GEMIN5	Abcam	Cat# ab201691
GFP	ChromoTek GmbH	Cat# 3h9-100; RRID: AB_10773374
HNRNP A1	Cusabio	Cat# PA010600HA01HU
HNRNP Q/R	Cell Signaling	Cat# 8588; RRID: AB_10897511
HSP90AB1	Cusabio	Cat# PA00109A0Rb
IFIT5	Cusabio	Cat# PA011023LA01HU
IRE1	Abcam	Cat# ab37073; RRID: AB_775780
MOV10	Cusabio	Cat# PA862068LA01HU
NGDN	Cambridge Bioscience	Cat# 16524-1-AP; RRID: AB_2152270
PA2G4	Cusabio	Cat# PA891987LA01HU
PPIA	Cusabio	Cat# PA07814A0Rb
PTBP1	Sigma	Cat# WH0005725M1; RRID: AB_1843067
RTCB	Cusabio	Cat# PA897546LA01HU
RPS10	Cusabio	Cat# PA02565A0Rb
RPS27	Sigma Aldrich	Cat# SAB4300952
SRPK1	Sino Biological Inc	Cat# 12249-MM03
TRIM25	Abcam	Cat# ab167154; RRID: AB_2721902
TRIM56	Abcam	Cat# ab154862
XRCC5	Cusabio	Cat# PA026233LA01HU
XRCC6	Cusabio	Cat# PA01617A0Rb
XRN1	Santa Cruz	Cat# sc-165985; RRID: AB_2304774
Donkey anti-Mouse IgG (H+L) Highly Cross-Adsorbed Secondary Antibody, Alexa Fluor 488	ThermoFisher Scientific	Cat# A-21202; RRID: AB_141607
Donkey anti-Rabbit IgG (H+L) Highly Cross-Adsorbed Secondary Antibody, Alexa Fluor 488	ThermoFisher Scientific	Cat# A-21206; RRID: AB_141708
Donkey anti-Rabbit IgG (H+L) Highly Cross-Adsorbed Secondary Antibody, Alexa Fluor 594	ThermoFisher Scientific	Cat# A-21207; RRID: AB_141637
Bacterial and Virus Strains		
pT7-SVmCherry	This paper	N/A
pT7-SVwt	Laboratory of L. Carrasco (Sanz and Carrasco, 2001)	N/A

(*Continued on next page*)

Continued

REAGENT or RESOURCE	SOURCE	IDENTIFIER
pNL4-3.R-E- Nef-mCherry	This paper	N/A
pNL4-3.R-E- Gag-mCherry	This paper	N/A
Chemicals, Peptides, and Recombinant Proteins		
L-Arginine HCL 13C, 15N	SILANTES GmbH	Cat# 201604102
L-Arginine HCL 13C	SILANTES GmbH	Cat# 201204102
L-Lysine HCL 13C, 15N	SILANTES GmbH	Cat# 211604102
4.4.5.5-D4-L-Lysine	SILANTES GmbH	Cat# 211104113
Cyclosporin A (CAS N° 59865-13-3)	Insight Biotechnology Ltd	Cat# sc-3503
Ganetespib (CAS N° 888216-25-9)	Cambridge Bioscience Ltd	Cat# 19432
Geldanamycin (CAS N° 30562-34-6)	Cambridge Bioscience Ltd	Cat# SM55-2
IRE1 Inhibitor III, 4μ8C (CAS N° 14003-96-4)	Merck Chemicals Ltd	Cat# 412512
WS6 (CAS N° 1421227-53-3)	Cambridge Bioscience Ltd	Cat# 17672
Critical Commercial Assays		
CellTiter 96 AQ_{ueous} One Solution Cell Proliferation Assay (MTS)	Promega	Cat# G3580
Deposited Data		
Proteome Xchange via PRIDE	Deutsch et al., 2017	PXD009789
RNA-seq via GEO		GEO: GSE125182
iCLIP via GEO		GEO: GSE125182
Experimental Models: Cell Lines		
HEK293	ECACC	Cat# 85120602 RRID:CVCL_0045
HeLa Kyoto	ATCC	Cat# CCL-2 RRID:CVCL_1922
Flp-In-T-Rex-293	Thermo Fisher Scientific	Cat#R78007 RRID:CVCL_U427
Flp-In-T-Rex-HeLa	Laboratory of M. Gromeier	N/A
BHK-21	ECACC	Cat# 85011433 RRID:CVCL_1915
Oligonucleotides		
CRISPR guide RNA targeting XRN1: AAUGCGAAACA ACACCUCCGUUUUAGAGCUAUGCUGUUUUG	Sigma-Aldrich Co Ltd	HS0000076809
TRIM25 left sgRNA: CCACGTTGCACAGCACCGTGTTC	This paper	N/A
TRIM25 right sgRNA: CTGCGGTCGCGCCTGGTAGACGG	This paper	N/A
Primers for cloning, see Table S6	This paper	N/A
Primers for RT-PCR, see Table S6	This paper	N/A
Recombinant DNA		
CRISPR/CAS9 plasmid: PX459 HSP90AB1_out_of_frame_67 guide sequence: CTCACACCTTGACTGCCAAG	This paper	N/A
CRISPR/CAS9 plasmid: PX459 PPIA_out_of_frame_57 guide sequence: GCCCGACCTCAAAGGAGACG	This paper	N/A
pOG44	ThermoFisher Scientific	Cat# V600520
pcDNA5/FRT/TO	ThermoFisher Scientific	Cat# V652020
pNL4-3.Luc.R-E-	NIBSC – Centre for AIDS Reagents	Cat# 2128
pNL4-3	NIBSC – Centre for AIDS Reagents	Cat# 2006
pHEF-VSVG	NIH AIDS Reagent Program	Cat# 4693
Software and Algorithms		
REST	Pfaffl, 2001	
STRING	Szklarczyk et al., 2017	https://string-db.org/

(*Continued on next page*)

Continued		
REAGENT or RESOURCE	SOURCE	IDENTIFIER
STAR	Dobin et al., 2013	https://github.com/alexdobin/STAR
Subread FeatureCount	Liao et al., 2013	http://bioinf.wehi.edu.au/subread-package/
SAMtools	Li et al., 2009	http://samtools.sourceforge.net/
RBDmap	Castello et al., 2016	https://www-huber.embl.de/users/befische/RBDmap/
DSseq2	Love et al., 2014	https://bioconductor.org/packages/release/bioc/html/DESeq2.html
Pheatmap	Kolde, 2015	https://cran.r-project.org/web/packages/pheatmap/index.html
iCount		https://github.com/tomazc/iCount
biomaRt	Durinck et al., 2009	https://bioconductor.org/packages/release/bioc/html/biomaRt.html
ggplot2	Wickham, 2009	https://cran.r-project.org/web/packages/ggplot2/index.html
MaxQuant (version 1.5.0.35)	Cox and Mann, 2008	https://www.maxquant.org/
Perseus	Tyanova et al., 2016	http://maxquant.net/perseus/
hom.Hs.inp.db	Carlson and Pages, 2015	http://bioconductor.org/packages/release/data/annotation/html/hom.Hs.inp.db.html
mRNAinteractomeHeLa	Castello et al., 2012	http://www.hentze.embl.de/public/RBDmap/
Semiquantitative test for protein differential analysis	This paper	N/A
limma (for moderated t test)	Smyth, 2004	https://bioconductor.org/packages/release/bioc/html/limma.html
ANOVA		https://www.itl.nist.gov/div898/handbook/eda/section3/eda355.htm

CONTACT FOR REAGENT AND RESOURCE SHARING

Further information and requests for resources and reagents should be directed to and will be fulfilled by the Lead Contact, Alfredo Castello (alfredo.castellopalomares@bioch.ox.ac.uk).

EXPERIMENTAL MODEL AND SUBJECT DETAILS

Cell culture

We used here human embryo kidney 293 cells (HEK293, ECACC #85120602), HeLa (ATCC cat. no. CCL-2) and baby hamster kidney cells (BHK-21, clone 13, ECACC #85011433); HEK293 Flp-In TREx are commercially available (Thermo Fisher Scientific, #R78007), while HeLa Flp-In TREx are a generous gift from Dr. Matthias Gromeier (Duke University Medical Center, Durham, NC, USA). All cells were cultured in DMEM with 10% FBS and 1x penicillin/streptomycin (Sigma Aldrich, #P4458) at 37°C with 5% CO_2. The media of Flp-In TREx (Tet-on) cells was supplemented with 15 μg/ml Blasticidin S and 100 μg/ml Zeocin. To generate RBP-eGFP-expressing cell lines, cells were transfected with pOG44 and the corresponding pcDNA5-FTR-TO plasmid (Table S6) using X-tremeGENE 9 DNA transfection reagent following manufacturer's recommendations (Sigma-Aldrich, #6365787001). For the selection of inducible cell lines, Zeocin was replaced by 150 μg/ml Hygromycin B as indicated in the manufacturer's manual (Thermo Fisher Scientific). Protein induction was achieved by supplementation of the medium with 1 μg/ml doxycycline. To generate KO cells, we transfected HEK293 using TRANSIT-CRISPR (Sigma-Aldrich) with SygRNAs assembled with Cas9 (Sigma-Aldrich, #CAS9PROT-50UG) and tracrRNA (Sigma-Aldrich, #TRACRRNA05N-5NMOL), followed by cell serial dilution and selection of KO cell clones. Alternatively, we generated px459 derived plasmids including sequences targeting the genes of interest (pX459 was a gift from Feng Zhang; Addgene plasmid #62988). These plasmids were transiently transfected into HEK293 cells using X-tremeGENE 9. Cells expressing the construct were selected with 1 μg/ml puromycine for 96 h, followed by cell serial dilution to obtain individual clones. To generate TRIM25 KO cells, HEK293 were transfected with 200 ng GeneArt CRISPR nuclease mRNA (Thermo Fisher Scientific, #A29378) along with 50 ng of two distinct, *in vitro* transcribed sgRNAs targeting sequences in exon 1 of the TRIM25 gene. Single cells were seeded, grown and checked for KO by western blotting.

Cell culture in SILAC media

Cells were grown in SILAC DMEM media (Thermo Scientific, #10107883) containing 10% dialysed FBS (Silantes GmbH, #281000900) and isotopic labeled arginine and lysine (Silantes GmbH amino acids: L-Arginine 13C,15N labeled #201604102; L-Arginine 13C labeled #201204102; L-Lysine 13C,15N labeled #211604102; 4.4.5.5.-D4-L-Lysine #211104113). Prior to experiments, we confirmed by mass spectrometry that the incorporation of isotopic labeled amino acids was superior to 98% using whole cell lysates.

Viruses

We used the SINV clone pT7-SVwt (Sanz and Carrasco, 2001) to generate the SINV suspension. The plasmid pT7-SVmCherry was generated by inserting mCherry after the duplicated subgenomic promoter in pT7-SVwt. To obtain SINV and SINV-mCherry viruses, pT7-SVwt and pT7-SVmCherry plasmids were first linearized with XhoI and used as a template for *in vitro* RNA transcription with HiScribe T7 ARCA mRNA kit (New England Biolabs, #E2065S). Transcribed genomic RNA was transfected into BHK-21 using Lipofectamine 2000 reagent (Invitrogen, #11668027). Viruses were collected from the supernatant 24 h later and cleared by centrifugation at 2000 rpm for 3 min followed by filtration with 0.45μm PVDF syringe filter units (Merck, #SLHV033RS). Cleared supernatants were titrated by plaque assay using BHK-21 cells.

Pseudotyped HIV-$1_{\text{Nef-mCherry}}$ and HIV-$1_{\text{Gag-mCherry}}$ were produced as follows. For HIV-$1_{\text{Nef-mCherry}}$, a sequence encoding the end of *env* followed by a linker, mCherry, T2A self-cleaving peptide and the beginning of Nef protein was synthesized using the GeneArt Gene synthesis service (Thermo Fisher Scientific), and cloned between the BamHI and XhoI restriction sites of pNL4-3.Luc.R-E- plasmid (NIBSC – Centre for AIDS Reagents, #2128), which is defective for Vpr and Env. For HIV-$1_{\text{Gag-mCherry}}$, a PSPXI restriction site flanked by flexible linker was introduced into *gag* of the pNL4-3 plasmid (NIBSC – Centre for AIDS Reagents, #2006) by overlapping PCR (primers in Table S6) as in (Müller et al., 2004). mCherry sequence was amplified by PCR flanked by PspXI restriction sites and cloned into pNL4-3 using the newly generated PspXI site. Finally, the fragment between SpeI and BamHI was replaced by that of pNL4-3.Luc.R-E-. Pseudotyped viral particles were produced by co-transfecting HEK293T cells (kindly provided by Prof. Jan Rehwinkel, University of Oxford, UK) with pNL4-3.R-E-$_{\text{Nef-mCherry}}$ or pNL4-3.R-E-$_{\text{Gag-mCherry}}$ plus pHEF-VSVG (NIH AIDS Reagent Program, #4693), which encodes for the glycoprotein of vesicular stomatitis virus (VSV).

METHOD DETAILS

RNA interactome capture

Comparative RNA interactome capture (cRIC) was performed based on the previously described protocol (Castello et al., 2012; Castello et al., 2013) with the following alterations: HEK293 cells, previously grown in media with isotopic labeled amino acids, were seeded in three sets of 3x15 cm dishes at 80% confluence, each set with a different SILAC label. One set of dishes remained uninfected and two sets were infected with SINV at a multiplicity of infection (MOI) of 10. One of these infected cell sets was incubated for 4 h and the other for 18 h. To correct for isotope-dependent effects, we permutated the SILAC labels between the three conditions in the three biological replicates. After incubation, cells were irradiated with 150 mJ/cm^2 of UV light at 254 nm, and lysed with 3 mL of lysis buffer (20 mM Tris-HCl pH 7.5, 500 mM LiCl, 0.5% LiDS wt/vol, 1 mM EDTA, 0.1% IGEPAL (NP-40) and 5 mM DTT). Lysates were homogenized by passing the lysate at high speed through a 5 mL syringe with a 27G needle, repeating this process until the lysate was fully homogeneous. 400 μl of lysate were taken for total proteome and transcriptome analysis (Figure 3; Tables S3 and S4). Protein content was measured using a kit compatible with ionic detergents (Thermo Fisher, Pierce 660nm Protein Assay Kit #22662 with IDC reagent #22663) and equal amounts of each of the three lysates were mixed. The final volume was adjusted to 9 mL and 1.5 mL of pre-equilibrated oligo(dT)$_{25}$ magnetic beads (New England Biolabs, #S1419S) were added and incubated for 1 h at 4°C with gentle rotation. Beads were collected in the magnet and the lysate was transferred to a new tube and stored at 4°C. Beads were washed once with 10 mL of lysis buffer, incubating for 5 min at 4°C with gentle rotation, followed by two washes with 10 mL of buffer 1 (20 mM Tris-HCl pH 7.5, 500 mM LiCl, 0.1% LiDS wt/vol, 1 mM EDTA, 0.1% IGEPAL and 5 mM DTT) for 5 min at 4°C with gentle rotation and two washes with buffer 2 (20 mM Tris-HCl pH 7.5, 500 mM LiCl, 1 mM EDTA, 0.01% IGEPAL and 5 mM DTT). Beads were then washed twice with 10 mL of buffer 3 (20 mM Tris-HCl pH 7.5, 200 mM LiCl, 1 mM EDTA and 5 mM DTT) at room temperature. Beads were resuspended in 900 μl of elution buffer and incubated for 3 min at 55°C with agitation. Eluates were stored at −80°C and beads were recycled as indicated in the manufacturer's manual, and re-used for two additional capture rounds. For RIC experiments followed by western blot analysis, we used the small scale RIC settings described in (Castello et al., 2013).

Conventional protein analyses

Samples were resolved on SDS-PAGE and analyzed by i) western blotting using specific antibodies, the Li-Cor Odyssey system for visualization and the Image Studio Lite software (Li-Cor) for quantification, ii) Coomassie blue staining with the InstantBlue Protein Stain reagent (Expedeon, #ISB1L) or iii) silver staining using SilverQuest kit (Invitrogen, #LC6070). Data shown in the manuscript are representative gels from at least three independent replicates. Details on antibodies can be found in the key resource table. Radioactive labeling of newly synthesized proteins was performed by replacing the growth media for 1 h with DMEM lacking methionine and cysteine and supplemented with Easytag EXPRESS^{35}S Protein Labeling Mix [^{35}S]Met-Cys (Perkin Elmer, #NEG772002MC). Samples were then analyzed by SDS-polyacrylamide gels (15%) followed by autoradiography.

Reverse-transcription and quantitative PCR

Total RNA was isolated using TRIzol (Invitrogen, #15596026). Reverse transcription was performed using Superscript III reverse transcriptase (Invitrogen, #18080044) with random hexamers priming (Invitrogen, #N8080127), following manufacturer's instructions. RT-qPCR analysis was performed with 2x qPCR SyGreen Mix Lo-ROX (PCRBiosystems, #PB20.11-01) and gene specific primers (Table S6) in a BioRad CFX96 Real-Time system, and analyzed with REST software (Pfaffl, 2001).

Plasmids and recombinant DNA procedures

Plasmids for generation of inducible cell lines were created by conventional cloning methods. Inserts were generally amplified from HEK293 cDNA or template plasmids using specific primers (Table S6). Inserts were cloned into the pcDNA5/FRT/TO with eGFP preceded or followed by a flexible linker encoding for GGSGGSGG (glycine and serine repeats) to facilitate the folding of the RBP of interest independently from the eGFP. For CRISPR/Cas9 expression plasmids, annealed oligos were inserted into the BbsI site of px459.

mCherry-based viral fitness assay

$5x10^4$ cells were seeded on each well of a 96-well microplate with flat µClear bottom (Greiner Bio-One, #655986) in DMEM lacking phenol-red and supplemented with 5% FBS and 1 mM sodium pyruvate. Cells (control, knock-out and Tet-on) were infected with SINV-mCherry at 0.1 MOI in complete DMEM (lacking phenol-red) with 2.5% FBS. Cells were incubated at 37°C and 5% CO_2 in a CLARIOstar fluorescence plate reader (BMG Labtech) for 24 h; eGFP and/or mCherry signal was monitored by measuring fluorescence (eGFP: excitation 470 nm, emission 515 nm; mCherry: excitation 570 nm, emission 620 nm) every 15 min. To monitor the shut off of protein synthesis with this method (Figure S1B), Tet-on HEK293 eGFP-control cells were induced with 1 µg/ml doxycycline for 4 h and then infected as indicated above. In experiments with HIV-1 mCherry replicons, $5x10^4$ cells were seeded on each well of a 96-well plate in clear DMEM supplemented with 2.5% FBS and 1 mM sodium pyruvate, and infected with pseudotyped HIV-$1_{Nef\text{-}mCherry}$ or HIV-$1_{Gag\text{-}mCherry}$. mCherry signal was monitored for 72 h in a fluorescence plate reader as indicated above. In overexpression experiments, Tet-on HEK293 cells expressing RBP-eGFP fusion proteins were either induced with 1 µg/ml doxycycline for 16 h or mock-induced and then infected with SINV-mCherry. In inhibitor assays, HEK293 cells were infected with SINV-mCherry as above and inhibitors or vehicle (DMSO) were added at 1 hpi at the concentrations indicated in the figures. Statistical significance of the difference in mCherry expression at 18 and 24 hpi was determined by t test (n = 9).

Drugs and cell viability assay

The following chemical inhibitors were used in this work: cyclosporin A (Insight Biotechnology Ltd, #sc-3503), Ganetespib (Cambridge Bioscience Ltd, #19432), Geldanamycin (Cambridge Bioscience Ltd, #SM55-2), 4µ8C (Merck Chemicals, #412512) and WS6 (Cambridge Bioscience Ltd, #17672). To test cell viability at the concentrations used, $5x10^4$ HEK293 cells were seeded on each well of a 96-well microplate with flat, transparent bottom and incubated with DMEM (no phenol red) supplemented with 5% FBS and 1 mM sodium pyruvate. 24 h later cells were treated with the compounds and incubated for another 24 h at 37°C and 5% CO_2. Cell viability was estimated by adding CellTiter 96 Aqueous One Solution (Promega, #G3580) and measuring 490 nm absorbance following the manufacturer's recommendations. To evaluate cell viability and proliferation in knockout cells, $2.5x10^4$ cells were seeded per well of a 96-well plate and incubated in DMEM (no phenol red, 5% FBS, 1mM sodium pyruvate) at 37°C and 5% CO_2. Cell viability was measured at the indicated times using CellTiter 96 Aqueous One Solution as described above. In parallel, the number of cells was counted using the Countess II FL Automated Cell Counter (Thermo Fisher Scientific).

Protein-protein interactions analysis

$4.2x10^6$ HEK293 Tet-on cells expressing eGFP or GEMIN5-eGFP proteins were seeded on a 10 cm dish and incubated with DMEM supplemented with 10% FBS and 1 µg/ml doxycycline. After 24 h, cells were infected with 10 MOI of SINV in DMEM lacking FBS and incubated for 1 h, followed by media exchange (DMEM with 1% FBS). Cells were harvested at 18 hpi and lysed in 1 mL of Triton-X-lysis buffer (10 mM Tris HCl pH 7.5, 150 mM NaCl, 1% Triton X-100, 5 mM $MgCl_2$, 5 mM DTT and 0.1 mM AEBSF serine protease inhibitor). For immunoprecipitation (IP), 40 µl GFP-Trap_A beads slurry (ChromoTek GmbH, #gta-20) were equilibrated in Triton-X-lysis buffer and then added to 500 µl of whole-cell lysate. Mixture was diluted with 4.5 mL of Triton-X-lysis buffer, and mixed with gentle rotation for 16 h at 4°C. GFP-Trap beads were washed once with Triton-X-lysis buffer, collecting the beads by gentle centrifugation after each wash (1000 g for 5 min at 4°C). In the second wash, the Triton-X-lysis buffer was supplemented with 1 µl/ml RNase A (Sigma Aldrich, #4642) and beads were incubated for 5 min at 37°C with gentle rotation. Beads were washed three additional times with Triton-X-lysis buffer. Proteins were released from the GFP-Trap beads via pH elution by resuspension in 50 µl 0.2 M glycine pH 2.5 for 30 s followed by collection of the beads through a quick spin. The supernatant was transferred to a new tube and neutralised with 5 µl of 1 M Tris base pH 10.4.

RBP-RNA interaction analysis: CLIP/RT-PCR

$6.5x10^5$ cells were seeded on each well of a 6-well plate and incubated in DMEM without phenol red and supplemented with 5% FBS and 1 µg/ml doxycycline. After 24 h, cells were either mock-infected or infected with SINV at a MOI of 10. At 18 hpi, culture media was removed and cells were irradiated with 150 mJ/cm^2 of UV light at 254 nm. Cells were lysed in 400 µl of lysis buffer (100 mM KCl,

5 mM $MgCl_2$, 10 mM Tris pH 7.5, 1% IGEPAL, 1 mM DTT, 100 U/ml Ribolock RNase inhibitor [ThermoFisher Scientific, #EO0381], 0.1 mM AEBSF, 200 μM ribonucleoside vanydil complex). Lysates were diluted with 5x high-salt buffer (1.25 M NaCl, 100 mM Tris pH 7.5, 0.1% SDS) and H_2O to reach 500 μl of 1x high-salt buffer. Lysates were then cleared by centrifugation (5000 rpm for 3 min at 4°C). Supernatants were transferred to a new tube and snap frozen in dry ice. An aliquot (50 μl) was taken as 'input'. Lysates were pre-cleared with 15 μl of pre-equilibrated control agarose beads (Pierce Control Agarose resin, Thermo Fisher Scientific, #26150) by incubation under gentle rotation for 30 min at 4°C followed by centrifugation at 1000 g for 2 min at 4°C. Supernatants were transferred to a new tube. 15 μl GFP-Trap_A bead slurry were equilibrated with 1x dilution buffer (500 mM NaCl, 1 mM $MgCl_2$, 0.05% SDS, 0.05% IGEPAL, 50 mM Tris pH 7.5, 100 U/ml Ribolock RNase inhibitor, 0.1 mM AEBSF), incubated with 1 mg/ml *E. coli* tRNA for 15 min and, after two washes with dilution buffer, they were added to the lysates. The mixture was incubated for 2 h at 4°C with gentle rotation and beads were recovered by centrifugation at 1000 g for 2 min at 4°C. Beads were washed twice with 100 μl of ice-cold high-salt buffer (500 mM NaCl, 20 mM Tris pH 7.5, 1 mM $MgCl_2$, 0.05% IGEPAL, 0.1% SDS, 100 U/ml Ribolock RNase inhibitor, 0.1 mM AEBSF), three times with 100 μl ice-cold low-salt wash buffer (150 mM NaCl, 20 mM Tris pH 7.5, 1 mM $MgCl_2$, 0.01% IGEPAL, 50 U/ml Ribolock RNase inhibitor) and resuspended in 50 μl of proteinase K buffer (100 mM NaCl, 10 mM Tris pH 7.5, 1 mM EDTA, 0.5% SDS). Protein digestion was carried out by incubation with 200 μg/ml of proteinase K (Invitrogen, #AM2546) for 30 min at 37°C with agitation (1100 rpm) and then raising temperature to 50°C for 1 h. After centrifugation at 1000 g and 4°C for 2 min, the supernatant containing the RNA was transferred to a low binding tube. RNA was then purified using RNeasy mini kit (QIAGEN, #74104) in parallel to the total RNA present in inputs. cDNA library was prepared with Superscript III reverse transcriptase and oligo$(dT)_{20}$ primer (Thermo Fisher Scientific, #18418020) following the manufacturer's recommendations. Finally, the presence of SINV sequences in cDNA libraries was detected by PCR using Phusion polymerase (New England Biolabs, #M0530S) and SINV C specific primers (Table S6).

Analysis of GEMIN5 binding sites by iCLIP

In order to identify GEMIN5 binding sites on SINV RNA at a high resolution, we employed iCLIP-seq (König et al., 2010). $10x10^6$ HEK293 Tet-on GEMIN5-eGFP cells were seeded in 5 sets of 3x15 cm dishes and induced for 24 h with doxycycline. Each cell set was then infected with 10 MOI of SINV. Similar procedure was carried out for 1 set 3x15 cm dishes of control HEK293 Tet-on eGFP cells with 8 h doxycycline induction. At 18 hpi, cells were washed with PBS 1x and UV irradiated with 150 mJ/cm^2 at 254 nm. Cells were then lysed with 1 mL of lysis buffer (NaCl 100 mM, $MgCl_2$ 5 mM, Tris pH 7.5 10 mM, IGEPAL 0.5%, SDS 0.1%, Na deoxycholate 0.5%, DTT 1 mM, 0.1 mM AEBSF) and the three plates of each condition set were pooled (3 mL of final volume). Lysates were then passed through a 27G needle three times and sonicated with three cycles of 10 s, with 15 s pause between pulses, using a Digenonde bioruptor at level M at 4°C. The homogenate was centrifuged 17900 g at 4°C for 10 min, and topped up to 3 mL with lysis buffer. To obtain RNA fragments of suitable length and to degrade DNA, 3 mL (replicates 1-2, control) or 1 mL (replicates 3-5) of thawed lysate was incubated with 20 U RNase I (Life Technologies, #AM2295) and 4 U Turbo DNase (Life Technologies, #AM2238) per ml of lysate for 3 min at 37°C, with 1100 rpm agitation. Subsequently, lysates were placed on ice and supplemented with 440 U RiboLock RNase Inhibitor. 40 μL of control agarose bead slurry per ml of lysate was pre-equilibrated in lysis buffer and resuspended in 50 μl of lysis buffer. Beads were added to the lysate and incubated for 30 min at 4°C with gentle rotation. The supernatants were then collected by centrifugation for 2 min at 4°C and 2500 g, and then incubated with 40 μL of pre-equilibrated GFP_trap_A beads per ml of lysate for 2 h at 4°C with gentle rotation. Next, the beads were collected by centrifugation (2 min, 4°C, 2500 g) and washed twice with 1 mL of high salt buffer (NaCl 500 mM, Tris HCl pH 7.5 20 mM, $MgCl_2$ 1 mM, IGEPAL 0.05%, SDS 0.1%, 0.1 mM AEBSF, 1 mM DTT), twice with 1 mL of medium salt buffer (NaCl 250 mM, Tris HCl pH 7.5 20 mM, $MgCl_2$ 1 mM, IGEPAL 0.05%, 0.1 mM AEBSF, 1 mM DTT), and twice with 1 mL of PNK wash buffer (20 mM Tris-HCl pH 7.4, 10 mM $MgCl_2$, 0.2% Tween-20) (replicates 1-2, GFP control) or low salt buffer (NaCl 150 mM, Tris HCl pH 7.5 20 mM, $MgCl_2$ 1 mM, IGEPAL 0.01%, 0.1 mM AEBSF, 1 mM DTT) (replicates 3-5). Beads were resuspended in 20 μL PNK mix [15 μL H_2O, 4 μL 5x PNK buffer pH6.5 (350 mM Tris-HCl pH 6.5, 50 mM $MgCl_2$, 25 mM DTT), 5 U of PNK enzyme (NEB, #M0201S), 20 U of Ribolock] and incubated for 20 min, at 37°C at 1100 rpm. Beads were then washed once with low salt buffer, once with high salt buffer, and twice with low salt or PNK wash buffer. Beads were then resuspended in 20 μL ligation mix [ligation buffer (50 mM Tris-HCl, 10 mM $MgCl_2$, 10 mM DTT), 10 U of RNA ligase (NEB, M0204S), 20 U of Ribolock, 1.5 μM pre-adenylated linker L3 (TriLink Biotechnologies, # T1-BGV01A), 4 μL PEG400 (Sigma-Aldrich, #202398-250G)] and incubated O/N at 16°C shaking at 1100 rpm. Subsequently, beads were washed with 500 μL of cold low salt or PNK wash buffer and three times with 1 mL of high salt buffer. Beads were transferred to a low binding tube during the third wash. The beads were further washed twice with 1 mL ice-cold low salt or PNK wash buffer and resuspended in 20 μL low salt or PNK wash buffer, 1x NuPAGE loading buffer (Invitrogen, #NP0007) and 100 mM DTT and denatured at 70°C (1200 rpm, 10 min). The supernatant was collected by centrifugation (1 min at 4°C and 2500 g), loaded on a 4%–12% Bis-Tris NuPage gel (Invitrogen, #NP0321) and run 90 min at 150 V in 1x MOPS running buffer (Life Technologies, #NP0001). Protein-RNA complexes were transferred to a membrane of nitrocellulose (30 V for 1 h). Region matching 190-280 kDa was then cut out, transferred to a fresh microfuge tube, topped up with 200 μL of proteinase K mix (80 mM Tris-Cl pH 7.4; 40 mM NaCl; 8 mM EDTA and 800 μg of proteinase K), and incubated for 20 min at 37°C and 1100 rpm. Subsequently, 200 μL of PKurea buffer (100 mM Tris-Cl pH 7.4; 50 mM NaCl; 10 mM EDTA; 7 M urea) was added and the sample then incubated for 20 min at 37°C at 1100 rpm. RNA was then phenol/chloroform extracted as in (Huppertz et al., 2014; König et al., 2010). Pellets were resuspended in 5 μL of nuclease free H_2O and stored at −20°C. Reverse transcription was carried out using Superscript III

(Life Technologies, #18080-044) and unique Rclip primers as in (Huppertz et al., 2014; König et al., 2010). The reaction was then transferred to a low DNA binding tube and precipitated with ethanol as in (König et al., 2010). The pellets were resuspended in 12 μL of 1x TBE-urea loading buffer, heated for 3 min at 80°C and separated on a 6% TBE-urea precast gel (Life Technologies, #EC6865BOX) for 40 min at 180 V. For replicates 1-2 and the control, the region of the gel corresponding to 85-200 nucleotides was cut off the gel and placed in a 0.5 mL microtube pierced with a needle inside a 1.5 mL microtube. Samples were spun at 16000 g for 1 min, and the flow-through topped up with 400 μl of diffusion buffer (0.5 M ammonium acetate, 10 mM magnesium acetate, 1 mM EDTA, 0.1% SDS) and incubated at 50°C for 30 min. For replicates 3-5, two regions of the gel containing cDNA fragments of 120-200 nucleotides and 85-120 nucleotides were cut off from the gel and crushed into small pieces using a pestle in 400 μL TE buffer. The samples were then incubated for 1 h at 37°C and 1100 rpm, placed on dry ice for 2 min, and incubated again for 1 h at 37°C and 1100 rpm. In all cases, the disrupted gel was then filtered by spinning through a Costar SpinX column (Sigma, #CLS8160-96EA) by centrifugation at 16000 g. The cDNA was then extracted using phenol/chloroform as in (König et al., 2010). Pellets were resuspended in 8 μL ligation mix [1x CircLigase Buffer II; 2.5 mM $MnCl_2$; 30 U of CircLigase II (Epicenter, #CL9025K)] and incubated for 1 h at 60°C. We next added 30 μL of oligo annealing mix [25 μL H_2O; 4 μL NEBuffer 4 (NEB, #B7004S); 0.3 μM cut_oligo (Sigma-Aldrich)] and the sample was heated for 1 min at 95°C followed by a temperature decrease of 1°C every 40 s until reaching 25°C. The samples were then digested with 2 μL of BamHI (Thermo Fisher, #FD0054) and incubated for 30 min at 37°C. After incubation at 80°C for 5 min, cDNA was ethanol precipitated (König et al., 2010). Pellets were resuspended in 20 μL H_2O and mixed with 1 μL of 10 μM primer mix P5/P3 Solexa and 20 μL Accuprime Supermix 1 (Life Technologies, #12342-010). The libraries were then amplified for 18 cycles (replicate 1), 23 cycles (replicate 2), 25 cycles (replicates 3-5) or 30 cycles (control GFP) and the products were then analyzed on a 6% TBE precast gel (Life Technologies, #EC6265BOX) in TBE buffer for 60 min at 140 V. The gel was stained with 1x TBE plus 1x SybrGold for 20 min (Life Technologies, #S11494) and bands of appropriate size cut out under blue light trans-illuminator. The gel slices were dissolved with a pestle in 100 μL diffusion buffer (0.5 M ammonium acetate; 10 mM magnesium acetate; 1 mM EDTA pH 8.0; 0.1% SDS), incubated for 30 min at 50°C at 1100 rpm and filtered in a Costar SpinX column as above. The library was purified using QIAquick Gel Extraction Kit (QIAGEN, #28704) and quantified on a Bioanalyser using a DNA high-sensitivity chip. Libraries were pooled for sequencing and processed using single-end sequencing mode with a NextSeq 500/550 High Output v2 kit (75 cycles, Illumina, #FC-404-2005).

Immunofluorescence and RNA FISH assays

High Precision Coverslips (Marienfeld, #0107052) were washed once in 1 M HCl for 30 min on a rocking machine, twice in double distilled water for 10 min and once in ethanol 70% for 10 min. 150,000 cells were seeded on the dried coverslips and incubated in DMEM with 10% FBS. In the case of the Tet-on cells, protein induction was performed with 1 μg/ml doxycycline. 16 h later cells were either mock-infected or infected for 1 h at 37°C with 10 MOI of SINV in DMEM without FBS, followed by the replacement of the medium with DMEM supplemented with 1% FBS. At the corresponding times post-infection, cells were rinsed once in PBS and fixed in 4% methanol-free formaldehyde for 10 min. After three 5 min washes in PBS, cells were permeabilised for 5 min with 1x PBS supplemented with 0.1% Triton X-100 (PBST). Next, cells were rinsed twice in PBST and once in PBST supplemented with 2% BSA, and blocked for 1 h with PBST supplemented with 2% BSA. Cells were later incubated for 1 h with primary antibodies (α-SINV C at 1:200 dilution or α-XRN1 at 1:50 dilution) in PBST + 2% BSA. Cells were subsequently rinsed in PBST + 2% BSA and washed three times with PBST + 2% BSA for 10 min. Cells were then incubated for 1 h in darkness with the secondary antibodies (α-rabbit Alexa488, α-rabbit Alexa594 or/and α-mouse Alexa488; Thermo Fisher Scientific, #A-21206, #A-21207, #A-21202 respectively) and/or GFP-Booster_Atto488 (ChromoTek GmbH, #gba488-100) at 1:500 dilution in PBST supplemented with 2% BSA. Cells were washed once with PBST supplemented with 2% BSA and three additional times with PBST supplemented with 2% BSA for 10 min. Cells were incubated with 2 μg/ml of DAPI in PBS for 5 min. Finally, cells were washed twice in PBST, once in PBS for 5 min, once in milliQ H_2O and mounted on glass slides using Vectashield Antifade mounting medium (Vector Laboratories, #H-1000).

For combined immunofluorescence and RNA FISH, cells were seeded in coverslips and fixed and permeabilised as described above. Then, cells were rinsed three times in PBST and incubated for 1 h with primary antibody (α-SINV C at 1:200 dilution) in PBST + 0.5 U/μl RiboLock RNase inhibitor. Next, cells were washed once in PBST and three additional times with PBST for 10 min. Cells were then incubated with secondary antibody (α-rabbit Alexa488 at 1:500 dilution) in PBST supplemented with 0.5 U/μl RiboLock RNase inhibitor for 1 h in darkness. Cells were washed once with PBST, and two additional times with PBST for 10 min, once in PBS for 10 min and fixed again in 4% methanol-free formaldehyde for 10 min. Cells were washed twice in PBS for 5 min, once in 1x PBS / 1x SSC for 5 min, once with 2x SSC for 5 min and twice with pre-hybridization buffer (2x SSC and 10% deionized formamide in DEPC water) at 37°C for 10 min. Next, cells were incubated with RNA probes [2 pmol/μl oligo$(dT)_{25}$ or oligo$(dA)_{25}$ coupled to Alexa 594 (Life technologies Ltd), or 125 nM SINV RNAs-specific Stellaris probes (LGC Biosearch Technologies)] in hybridization buffer (2x SSC, 10% deionized formamide and 10% dextran sulfate in DEPC water) for 16 h at 37°C in a wet chamber. In the case of Tet-on cells expressing GEMIN5-eGFP or MOV10-YFP proteins, GFP-Booster_Atto488 (1:500 dilution) was included at this step. Cells were subsequently washed twice with pre-hybridization buffer for 10 min at 37°C and incubated for 5 min at 37°C with 2 μg/ml DAPI in pre-hybridization buffer. Finally, cells were washed twice with 2x SSC for 5 min, twice with 1x PBS, once for 5 min with 1x PBS and once in milliQ H_2O. The coverslip was mounted immediately after on glass slides using Vectashield.

In both cases, images were acquired on an API DeltaVision Elite widefield fluorescence microscope using a 100X oil UPlanSApo objective (1.4 NA) and deconvolved with SoftWoRx v6.5.2 (GE Healthcare). Fluorescence intensity profiles were obtained using the

script "Multichannel Plot Profile" in the BAR collection for ImageJ (https://imagej.net/BAR). In Figures 4 and S4, RBPs were classified as 'enriched' when accumulating in viral factories co-localizing with SINV C; 'absent' when undetectable in viral factories; and 'diffused' RBPs when distributed across the cytoplasm and thus present but not enriched in viral factories.

Determining the percentage of infected cells

9x10^5 HEK293 cells were seeded on washed coverslips and incubated in DMEM minus phenol red + 5% FBS + 1 mM sodium pyruvate for 24 h. Cells were infected with different MOI of SINV-mCherry in complete DMEM (lacking phenol-red) with 2.5% FBS. At 18 hpi, cells were fixed and processed for immunofluorescence as indicated above using α-SINV C antibody and DAPI. Images were acquired on an API DeltaVision Elite widefield fluorescence microscope using a 60X oil PlanApo objective (1.42 NA). The percentage of infected cells was calculated by counting C-expressing cells and the total number of DAPI-stained cells using the "Cell Counter" plugin in ImageJ. To define the MOI of SINV used in cRIC experiments and fitness assays, different concentration of viruses were tested. We selected 10 MOI for cRIC experiments because it is the minimal dose promoting high percentage of infected cells in a reproducible manner. We selected 0.1 MOI for fitness experiments as it allows optimal measurement of the mCherry fluorescence in the CLARIOstar plate reader.

Mass spectrometry

cRIC inputs (whole cell lysates) and eluates were processed following the filter aided sample preparation (FASP) as in (Castello et al., 2013). GEMIN5-eGFP and eGFP IPs were processed with a single-pot solid-phase-enhanced sample preparation (SP3) protocol using 70% acetonitrile for protein binding (Sielaff et al., 2017). All samples were acidified with 5% formic acid prior to mass spectrometric analysis.

Peptides from the cRIC inputs, and GEMIN5-eGFP and eGFP IPs were analyzed on an Ultimate 3000 ultra-HPLC system (Thermo Fisher Scientific) and electrosprayed directly into a QExactive mass spectrometer (Thermo Fisher Scientific). They were initially trapped on a C18 PepMap100 pre-column (300 μm inner diameter x 5 mm, 100Å, Thermo Fisher Scientific) in solvent A (0.1% [vol/vol] formic acid in water). The peptides were then separated on an in-house packed analytical column (75 μm inner diameter x 50cm packed with ReproSil-Pur 120 C18-AQ, 1.9 μm, 120 Å, Dr. Maisch GmbH) using a linear 15%–35% [vol/vol] acetonitrile gradient (2 h for whole cell lysates and 1 h for protein-protein interaction samples) and a flow rate of 200 nl/min. Full-scan mass spectra were acquired in the Orbitrap (scan range 350-1500 m/z, resolution 70000, AGC target 3×10^6, maximum injection time 50 ms) in a data-dependent mode. After the mass spectrum scans, the 20 (for whole cell lysates) or 10 (GEMIN5 IPs) most intense peaks were selected for higher-energy collisional dissociation fragmentation at 30% of normalized collision energy. Higher-energy collisional dissociation fragmentation spectra were also acquired in the Orbitrap (resolution 17500, AGC target 5×10^4, maximum injection time 120 ms) with first fixed mass at 180 m/z.

For cRIC eluates, liquid chromatography (LC) was performed using an EASY-nano-LC 1000 system (Thermo Fisher Scientific) in which peptides were initially trapped on a 75 μm internal diameter guard column packed with Reprosil-Gold 120 C18, 3 μm, 120 Å pores (Dr. Maisch GmbH, #r13.9g) in solvent A using a constant pressure of 500 bar. Peptides were then separated on a 45°C heated EASY-Spray column (50 cm x 75 μm ID, PepMap RSLC C18, 2 μm, Thermo Fisher Scientific #164540) using a 3 h linear 8%–30% [vol/vol] acetonitrile gradient and constant 200 nl/min flow rate. Peptides were introduced via an EASY-Spray nano-electrospray ion source into an Orbitrap Elite mass spectrometer (Thermo Fisher Scientific). Spectra were acquired with resolution 30000, m/z range 350-1500, AGC target 1x10^6, maximum injection time 250 ms. The 20 most abundant peaks were fragmented using CID (AGC target 5x10^3, maximum injection time 100 ms, normalized collision energy 35%) in a data dependent decision tree method.

Peptide identification and quantitation of all proteomics experiments was then performed using MaxQuant (v1.5.0.35) (Cox and Mann, 2008). Data were searched against the Human Uniprot database (version, January 2016) alongside a custom database including all the known SINV polypeptides and a list of common contaminants provided by the software. eGFP protein sequence was included in the analysis of GEMIN5-eGFP and eGFP IPs (Uniprot ID C5MKY7). The search parameters for the Andromeda search engine were: full tryptic specificity, allowing two missed cleavage sites, fixed modification was set to carbamidomethyl (C) and the variable modification to acetylation (protein N terminus), oxidation (M). Match between runs was applied. All other settings were set to default, leading to a 1% FDR for protein identification. Raw and processed proteomic data have been deposited to the ProteomeXchange Consortium (Deutsch et al., 2017) via the PRIDE partner repository with the dataset identifier PXD009789.

RNA sequencing

RNA from the 'inputs' (whole cell lysate) of cRIC experiments was extracted using TRIzol. Strand-specific RNA-seq was performed with 100 ng of total RNA. Libraries were prepared using NEBNext Ultra Directional RNA library Prep Kit for Illumina (New England Biolabs, #E7420S) according to manufacturer instructions. In brief, RNA was fragmented for 15 min at 94°C and then reverse transcribed. cDNA and double-stranded cDNA was purified with AMPure XP beads (Beckman Coulter, #A63881). After end repair, NEBNext Adaptors for Illumina (New England Biolabs, #E7335S) were ligated onto the cDNA according to the kit manual. Libraries were amplified by 15 cycles of PCR. We used the following combination of barcodes for sample multiplexing: S1_Mock ATCACG, S1_SV4h CGATGT, S1_SV18h TTAGGC, S2_Mock ACAGTG, S2_SV4h CAGATC, S2_SV18h ACTTGA, S3_Mock GATCAG, S3_SV4h TAGCTT and S3_SV18h GGCTAC. Libraries with an average length of 320 nt were pooled and sequenced with an Illumina

NextSeq instrument, using 78 nt paired-end sequencing mode with a NextSeq 500/550 High Output v2 kit (150 cycles, Illumina #FC-404-2002). Raw and processed RNA-seq are available at GEO: GSE125182.

QUANTIFICATION AND STATISTICAL ANALYSES

Proteomic quantitative analysis

To compare the cRIC inputs and eluates under different conditions, peptide intensity ratios between two samples were computed and summarized. The log2-intensity ratio of each protein was tested to be different from zero in the three biological replicates using moderated t test, which is implemented in the R/Bioconductor package limma (Smyth, 2004). p values were corrected for multiple testing by controlling the false discovery rate with the method of Benjamini-Hochberg. For proteins for which the protein intensity was ‘zero’ in one of the two conditions, we applied a semiquantitative approach that assumes that proteins without quantitative information are below the detection limit (Sysoev et al., 2016). The approach compiles the number of replicates in each condition in which a given protein has an intensity value. When comparing 2 conditions and three biological replicates, this leads to a matrix with 16 different groups (detected 0, 1, 2 or 3 times in condition 1 versus detected 0, 1, 2 or 3 times in condition 2). A protein is classified as ‘altered RBP’ by the semiquantitative method if an intensity value is assigned to it in 3 or 2 of the replicates in one of the two conditions, while only 1 or 0 intensity values are detected in the other condition.

The fraction of RNA-bound RBPs was determined by computing the ratio between the protein intensity of each individual RBP in the cRIC eluates and that in the whole cell lysate (Figure S3D). Hence, this calculation reflects amount of protein crosslinked to RNA (cRIC eluates) divided by the total amount of protein (cRIC inputs).

Results were visualized using the R package ggplot2 (Wickham, 2009). To assess the scope of previously known RBPs within the RBPome of uninfected and SINV-infected HEK293 cells, proteins identified by cRIC here were compared to those compressing the superset of human RBPs reported in (Hentze et al., 2018). GO annotations were obtained from the R package mRNAinteractomeHeLa (http://www.hentze.embl.de/public/RBDmap/) (Castello et al., 2012) (Key Resources Table), and gene set enrichment analysis was performed by applying Fisher’s exact test to categories of GO annotations with at least three annotated proteins.

We compared the repertoire of RBPs with differential RNA-binding activity at 18 hpi (Table S1) with the mouse ribo-interactome (Table S2) (Simsek et al., 2017). Specifically, we considered proteins in the Table S3 of (Simsek et al., 2017) with negative predictive values (NPV) $\geq$ 0.99 in puromycin and RNase samples as ‘ribosome-associated proteins’, as described in that study. To find mouse orthologs for RBPs responding to SINV infection, we used the R package biomaRt to identify ENSEMBL peptide IDs for our RBPome dataset and hom.Hs.inp.db (Carlson and Pages, 2015) to provide mapping between human and mouse proteins using these IDs (Key Resources Table). If a mouse ortholog of an altered RBP identified at 18 hpi was found in the ‘ribo-interactome’ (Simsek et al., 2017) or if the gene symbols between human and mouse matched directly, the human RBP was considered as ‘ribosome-associated’. Results of this analysis are provided in Table S2.

For GEMIN5 protein-protein interaction analysis, protein quantification was performed by label free quantification using MaxQuant. Ratios were compiled and normalized to eGFP protein intensity in each sample, which is expected to be the same across samples. Significance of the fold changes was estimated by t test using the software Perseus (Tyanova et al., 2016). We performed three main comparisons with the data from the IPs: i) GEMIN5-eGFP versus eGFP both in uninfected cells; ii) GEMIN5-eGFP versus eGFP both in SINV-infected cells; and iii) GEMIN5-eGFP in uninfected cells versus GEMIN5-eGFP in SINV-infected cells (Figure 7C, left, middle and right, respectively). Resulting data are summarized in Table S5. Raw and processed proteomic data from GEMIN5-eGFP IPs have been deposited to the ProteomeXchange Consortium via the PRIDE partner repository with the dataset identifier PXD009789.

The R package ggplot2 was utilized to visualize GEMIN5-eGFP proteomics data in volcano plots (Figures 7C). Only proteins that were identified as high-confidence interactors of GEMIN5-eGFP (i.e., p value < 0.01 and positive log2 fold change) in the left panel of Figure 7C were displayed in the comparison between infected and uninfected cells in the right panel. Proteins with names starting with ‘RPS’ or ‘RPL’, were classified as ‘ribosomal’ and displayed in the volcano plots as pink dots.

STRING (Szklarczyk et al., 2017) was used to display the connectivity between altered RBPs in SINV-infected cells (Figures S2C and S2D) and between the proteins comprising the GEMIN5 interactome (Figure S7D). Protein networks were generated using the following parameters: display – confidence; Interaction sources – experiments and databases; interaction score – high-confidence (0.700). Disconnected nodes were hidden from display and nodes colored based on functional enrichment within the network as determined by STRING. GEMIN5 protein interactome (Figure S7D) was defined as proteins enriched in GEMIN5-eGFP IPs over eGFP IPs with p value < 0.01. STRING-based GO enrichment for GEMIN5 protein interactome is provided in Table S5.

RNA sequencing data analysis

We combined the human genome (version hg38) with SINV sequence as our reference genome. RNA-seq reads were then mapped to this reference genome using STAR (Dobin et al., 2013). Reads mapping to each transcript were counted with *featureCounts* in Subread software package (Liao et al., 2013). Only uniquely mapped reads are considered for counting. SINV infection is known to shut off transcription globally (Gorchakov et al., 2005), which may bias (underestimate) differential expression results if normalization is carried out assuming that overall RNA abundance remains unchanged. Therefore, we decided to normalize reach counts in each condition to the corresponding rRNA expression by dividing a factor proportional to the total rRNA read counts in 3 conditions

(0.899, 1 and 0.473 for Mock, 4 hpi and 18 hpi respectively). We confirmed by RT-qPCR that rRNA does not change in abundance in course of infection. The R package DESeq2 (Love et al., 2014) was used for differential gene expression analysis based on rRNA normalized read counts. As DESeq2 requires the reads counts to be un-normalized and in the form of integer values, rRNA normalized read counts were rounded to the closest integer to make the "DESeqDataSet" to start the differential analysis. We estimated the size factor of each sample separately in DESeq2, instead of pooling all the samples prior to estimating this parameter.

Differential RNA expression between infected (4 and 18 hpi) and uninfected cells was visualized in MA plots (Figures 3F and 3G) using DESeq2. To visualize the overall effect of experimental covariates and potential batch effects, a principal component plot of the samples was generated using the plotPCA function in DESeq2, based on the principal component analysis (PCA) of the variance stabilized expression of the top 500 genes with the highest expression variance among samples. As shown in Figure S3F, the variance explained by the first and second PC (on X and Y-axes) combined accounts for a high percentage (96%) of the total variance, and samples within the same condition clustered better between them than with the other two conditions. It is interesting to note that the first PC along accounts for 94% of the total variance, and it distinctly separates 18 hpi to the other samples (i.e., uninfected and 4 hpi), indicating that the cellular transcriptome is dramatically altered at 18 hpi.

Genes related to GO terms 'Response to virus' (GO:0009615) and 'Defense response to virus' (GO:0051607) were extracted from "hsapiens_gene_ensembl" dataset (GRCh37) from Bioconductor package biomaRt (Durinck et al., 2009) and plotted as a heatmap using the R package pheatmap (Kolde, 2015) (Figure S1E). This package was also used to make a heatmap for differentially expressed cellular RNAs, including those transcripts passing the following thresholds: i) log2 fold change > 3 or < -3 and ii) adjusted p value < 0.01 (Figure S3E).

Reads mapping to positive and negative strands of viral RNAs were separated using SAMtools *view* utility (Li et al., 2009). In Illumina reverse paired end sequencing, paired reads came from opposite strands. Therefore, reads with the second pair mapping to the positive strand, or with the first pair mapping to the negative strand, were both counted as mapping to the positive strand and vice versa. The total read counts mapping to each strand were compiled and counted using SAMtools *merge* and SAMtools *depth*, respectively.

Analysis of RNA synthesis, processing, and degradation

We used analysis of variance (ANOVA) to evaluate in what extent the changes in transcript levels are explained by the rate of RNA synthesis, processing and degradation. The measurement of the rate of these RNA processes for each individual RNA were obtained from (Mukherjee et al., 2017). We built a multiple linear regression using the rate of the above-mentioned RNA processes as 'predictors' or 'factors', and the transcriptome changes in SINV infected cells as the 'response variable'.

$$Ti = A0i + A1iDi + A2iPi + A3iSi + Ei$$

i indicates all the individual RNA molecules; Ti is the expression change for the molecule between the two conditions compared; A0i is the regression intercept; Di, Pi and Si are the rate of degradation, processing and synthesis, respectively; Ei is the 'error term' in the multiple linear regression.

After fitting the model, the total variance explained, or R-squared, is defined as the sum of squares (SS) contributed to the total SS by different factors, i.e., the three predictor variables and the error term, as indicated in the equation below:

$$SStotal = SSDegration + SSProcessing + SSSynthesis + SSerror$$

Therefore, the contribution of the three predictors to the alterations in the transcriptome can be measured by their proportion of SS. The partial SS for each predictor is obtained using the "sequential sum of squares" method implemented in ANOVA function in R (Key Resources Table). These data (mock compare to 4 hpi and mock compare to 18 hpi) are shown in Figure 5A. A more detailed description of ANOVA can be found in NIST/SEMATECH e-Handbook of Statistical Methods (https://www.itl.nist.gov/div898/handbook/eda/section3/eda355.htm).

iCLIP-seq data processing

To identify GEMIN5 binding sites on SINV RNAs, reads in the fastq files from sequencing were demultiplexed to separate the samples according to the sample barcodes. Molecular and sample barcodes as well as trailing adaptor sequences were trimmed off. Molecular barcode information was stored in the read name. Reads were then mapped to a combined human (GRCh38) and SINV genome (pT7-SVwt) sequence using STAR. Uniquely aligned reads were then extracted using SAMtools. Binding sites were determined as the 5′-most base of each uniquely mapped read. PCR duplicates were identified as reads with the same mapping position and molecular barcode and each unique fragment counted just once. The 5′-most base in sequenced reads corresponds to the base directly 3′ of the crosslinked base. The number of unique fragment counts per position gives a measure of GEMIN5 interaction strength with that position along the RNA.

Due to the sheer abundance of SINV RNA at 18 hpi, some background signal could be observed in GFP control. To account for this background, GFP signal was subtracted from GEMIN5 signal after correction to total SINV reads. Signal along SINV was then visualized individually per replicate (Figure S7) and as an average of all five replicates (Figure 7) as a coverage track and heatmap.

Because the binding sites are narrow (sharp) and hence difficult to see when plotting the full SINV region, the plot shows an average over a sliding window of 20 nt. Note that the negative signal from y axis (higher signal in GFP) is cut off to better highlight GEMIN5 enriched regions.

Significantly crosslinked sites were determined using iCount peaks (Key Resources Table). iCount peaks was run to generate a background distribution by randomly distributing the crosslinked sites a hundred times along the SINV genome and compare the actual observed distribution to this background to generate a false discovery rate. Since regions corresponding to genomic, subgenomic and 3′ end region have different overall abundance, they were indicated as individual gene segments in the calculation to account for potentially higher background. Sites meeting FDR cutoff of 0.01 within 5 nt of each other were then merged using iCount clusters to form binding sites. Binding sites were then given a 'strength score' calculated as counts within the binding site divided by its width, and visualized in a heatmap in five bins to differently highlight the strengths of binding at different sites (Figures 7 and S7). This process was done for the GEMIN5 replicates separately as well as for the library size normalized average of the five replicates. Figure 7D additionally shows a heatmap that indicates how many replicates support a genomic position as binding site when determined individually per replicate. ggplot2 was used to facilitate plotting the heatmaps.

To look at base composition around the start of the SINV sgRNA, the 5′-most base of unique fragments was extracted from aligned reads taking softclipping into account. Count per base relative to total count in the sgRNA region is show in Figure S7H to indicate relative binding site frequency and whether the sequenced base matches the genome.

Raw and processed iCLIP-seq data are available at GEO: GSE125182.

DATA AND SOFTWARE AVAILABILITY

The accession number for the mass spectrometry data reported in this paper is ProteomeXchange: PXD009789. The accession number for the RNA-seq and iCLIP data reported in this paper is GEO: GSE125182.

Molecular Cell, Volume *74*

Supplemental Information

System-wide Profiling of RNA-Binding Proteins Uncovers Key Regulators of Virus Infection

Manuel Garcia-Moreno, Marko Noerenberg, Shuai Ni, Aino I. Järvelin, Esther González-Almela, Caroline E. Lenz, Marcel Bach-Pages, Victoria Cox, Rosario Avolio, Thomas Davis, Svenja Hester, Thibault J.M. Sohier, Bingnan Li, Gregory Heikel, Gracjan Michlewski, Miguel A. Sanz, Luis Carrasco, Emiliano P. Ricci, Vicent Pelechano, Ilan Davis, Bernd Fischer, Shabaz Mohammed, and Alfredo Castello

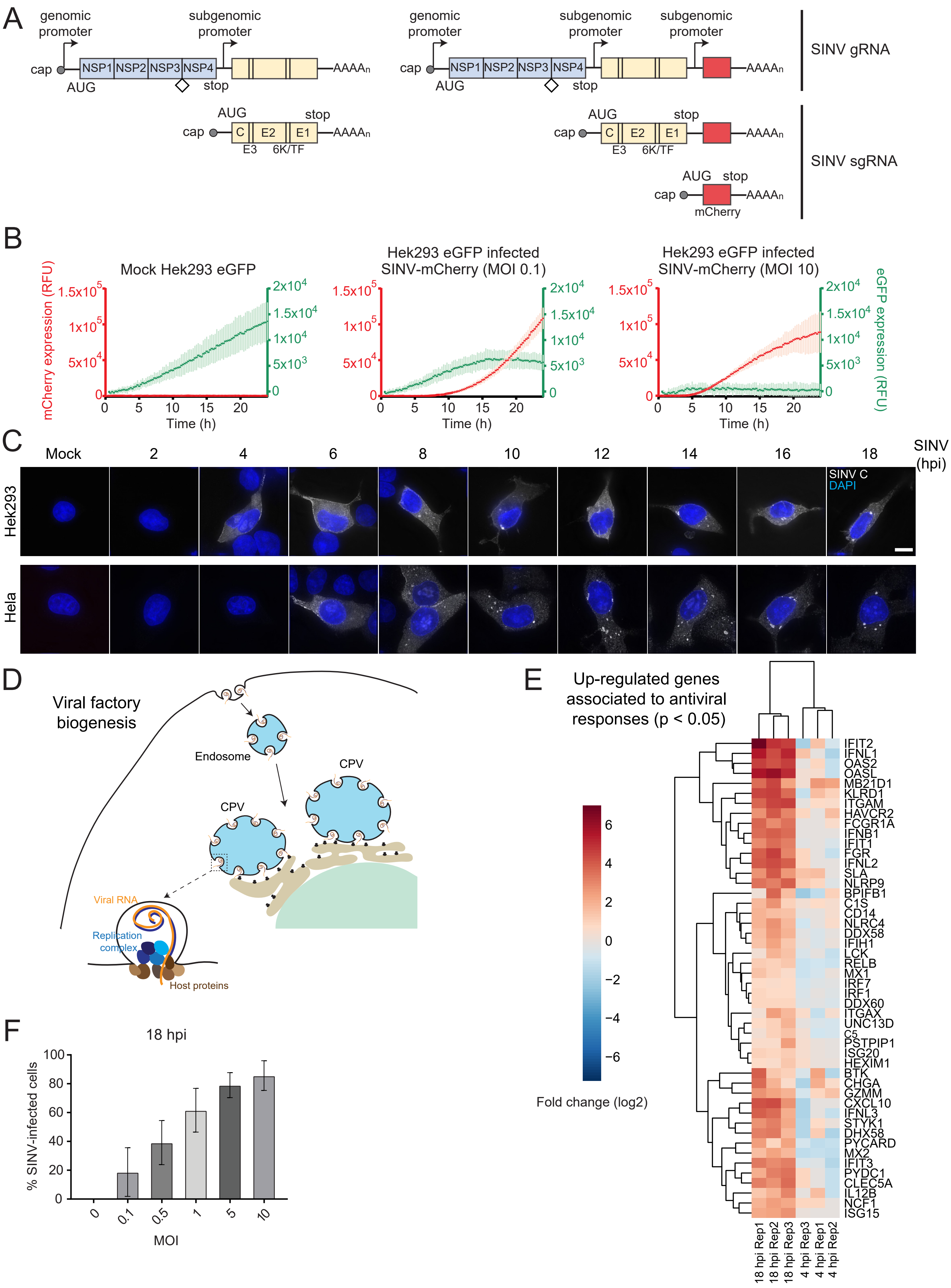

Figure S1 (Garcia-Moreno et al)

Figure S1. Analysis of SINV-induced molecular signatures in different cellular models; related to Figure 1. A) Schematic representation of SINV genomic (g) and subgenomic (sg) RNAs. The diamond represents the opal termination codon between NSP3 and NSP4. B) Production of eGFP and mCherry in stable Tet-on HEK293-eGFP cells infected with SINV-mCherry at 0.1 or 10 multiplicity of infection (MOI). Green and red fluorescence were measured every 15 min in a plate reader with atmospheric control (5% CO_2 and 37°C). eGFP synthesis serves as a proxy of host cell gene expression. Virus-induced shut off is reflected by the blockage of eGFP accumulation, which occurs at 5 hpi with 10 MOI (in agreement with the [^{35}S]-Met/cys labelling analysis shown in Figure 1C) and at 15 hpi with 0.1 MOI. RFU, relative fluorescence units. C) Localisation by immunofluorescence of SINV C in SINV-infected HEK293 and Hela cells. SINV C accumulates in cytoplasmic foci known as the viral replication factories, which are detected in both HEK293 and HeLa at similar times post infection. D) Schematic representation of viral factory biogenesis. CPV, cytopathic vacuoles containing spherules where viral RNA is replicated. E) Heatmap representing the log2 fold expression change determined by RNAseq analysis of RNAs enriched with $p<0.05$ in SINV-infected (4 or 18 hpi) versus uninfected HEK293 cells and annotated by 'antiviral response' and 'innate immunity' gene ontology (GO) terms. These GO terms were statistically enriched in infected over uninfected cells. Note the presence of interferons (IFNs), interferon stimulated genes (ISG), interferon induced proteins (IFI), and interferon regulatory factors (IRF). F) Percentage of HEK293 cells expressing SINV C after infection with SINV-mCherry at different MOI. Cells were processed for immunofluorescence staining with α-SINV C antibody and DAPI. 10 fields were counted for each independent experiment (n=3). Bars represent the mean and error bars represent the standard deviation (SD).

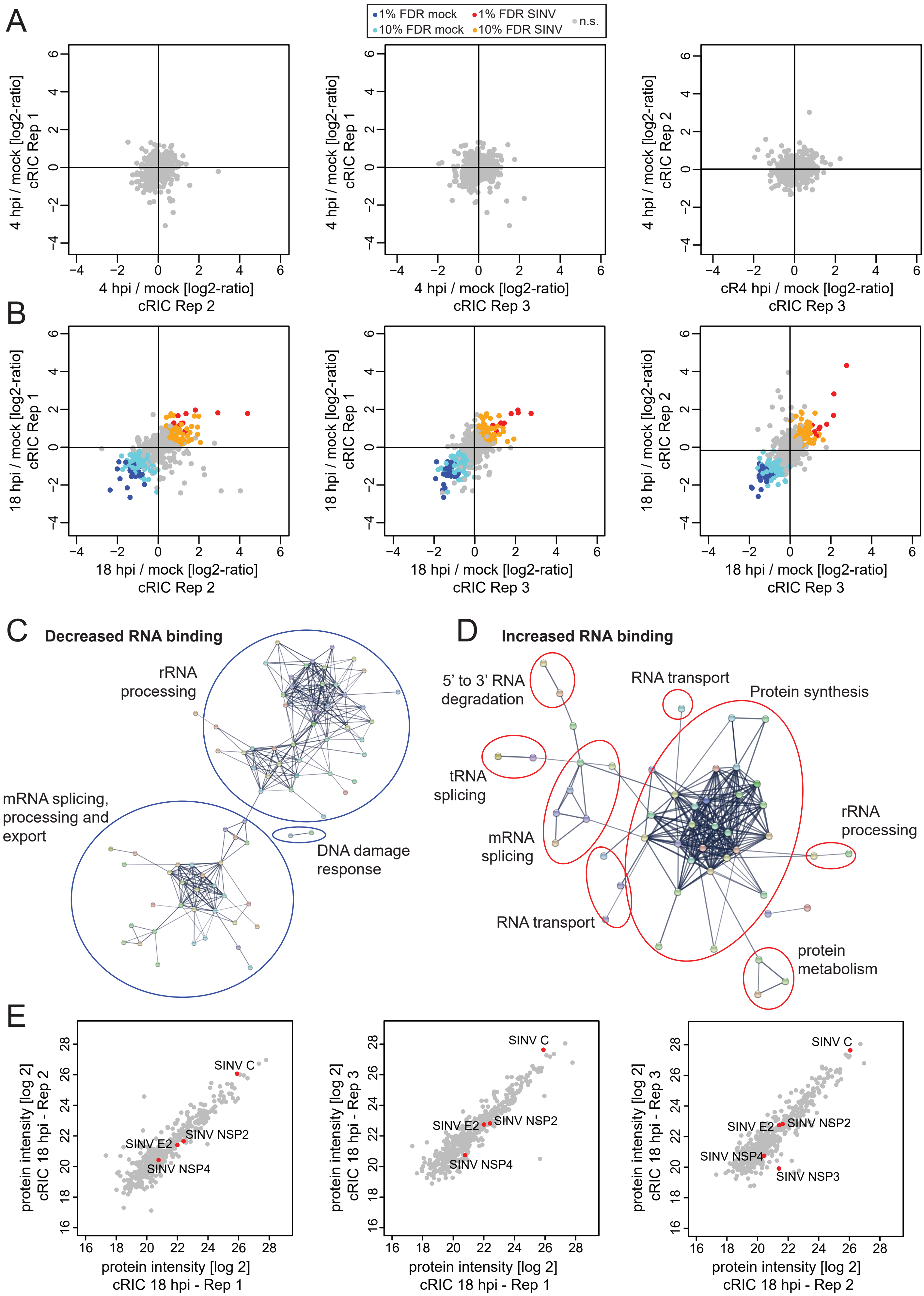

Figure S2 (Garcia-Moreno et al)

Figure S2. Analysis of the RNA-bound proteome in SINV-infected HEK293 cells by cRIC; related to Figure 2 and Table S1. A) Scatter plots displaying the intensity ratio for each protein (dots) in the cRIC eluates from cells infected with SINV for 4 h over uninfected cells, analysed by quantitative proteomics. Plots show the experimental reproducibility as each axis indicate the ratios obtained in one biological replicate. The three scatter plots represent the pair-wise comparison of the three replicates. B) As in (A) but comparing 18 hpi versus uninfected cells. Proteins enriched in infected cells with 1% or 10% FDR are shown in red and orange, respectively. Proteins enriched in uninfected cells with 1% or 10% FDR are shown in blue or cyan, respectively. Grey dots represent non-enriched proteins. FDR, false discovery rate; n.s. non-significant. C) STRING network showing the protein-protein interaction map between SINV-inhibited RBPs (both 1% and 10% FDR). D) As in (C) but with SINV-stimulated RBPs. E) Scatter plots comparing the raw intensity of each protein in the eluates of cRIC from cells infected with SINV for 18 h. Viral proteins are highlighted in red. The three scatter plots display the pair-wise comparison between all three replicates.

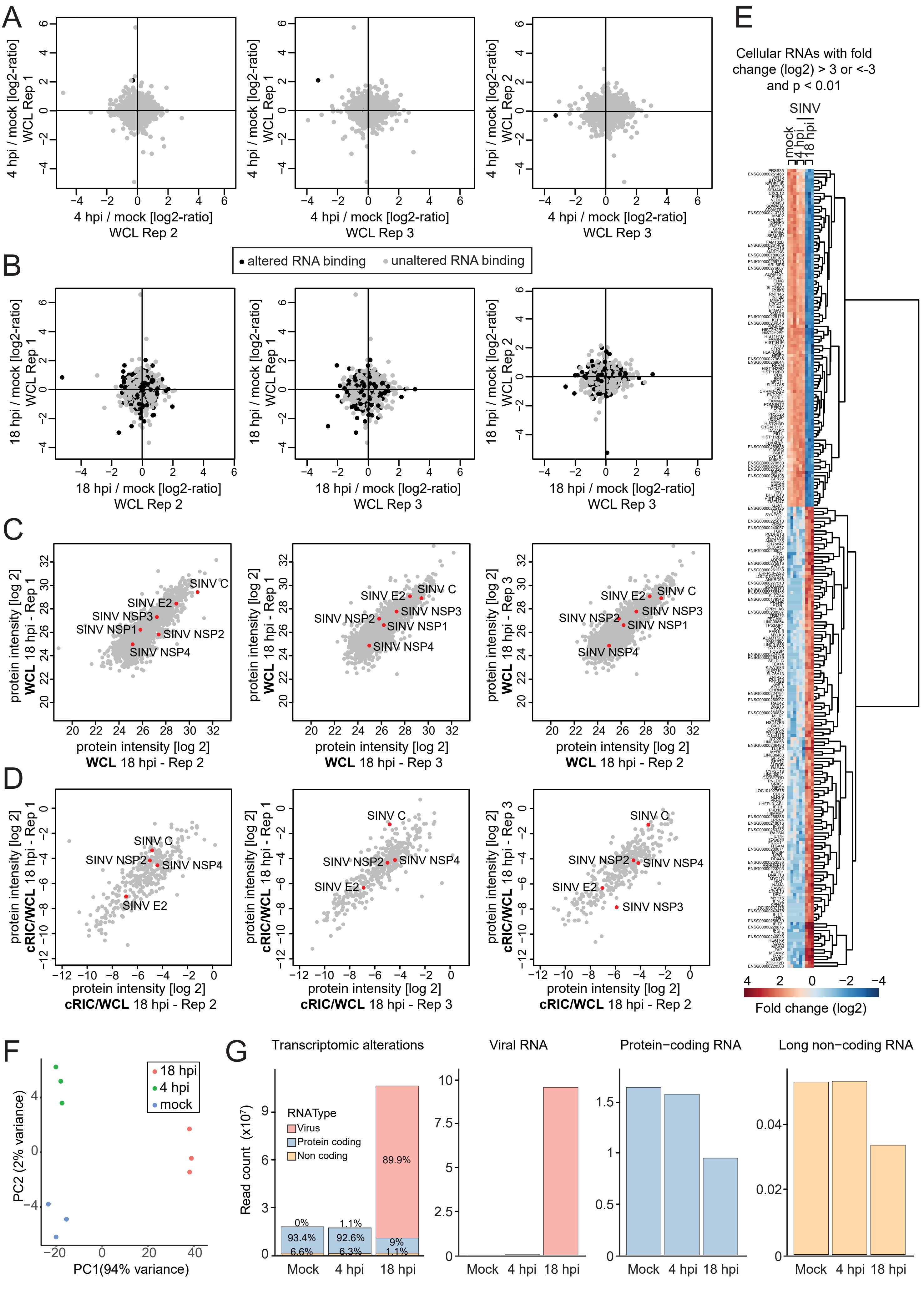

Figure S3 (Garcia-Moreno et al)

Figure S3. Proteomic and transcriptomic analyses of whole SINV-infected cell lysates; related to Figure 3 and Tables S3-4. A) Scatter plots displaying the intensity ratio for each protein (dots) in the cRIC inputs from cells infected with SINV for 4 h over uninfected cells, analysed by quantitative proteomics. Plots show the experimental reproducibility as each axis indicate the ratios obtained in one biological replicate. The three scatter plots represent the pair-wise comparison of the three replicates. Black dots represent proteins significantly enriched in the eluates of cRIC experiments either at 4 hpi or in uninfected conditions (Figure 2A and S2A). WCL, whole cell lysate. B) As in (A) but between 18 hpi and uninfected conditions. Black dots represent proteins significantly enriched in the eluates of cRIC experiments either at 18 hpi or in uninfected conditions (Figure 2C and S2B). C) Scatter plots comparing the raw intensity of each protein in the inputs of two cRIC replicates from cells infected with SINV for 18 h. The three scatter plots display the pair-wise comparison between all three replicates. Viral proteins are highlighted in red. D) Scatter plots comparing the intensity of each protein in the cRIC eluates normalised to the intensity in the inputs of two biological replicates from cells infected with SINV for 18 h. The three scatter plots display the pair-wise comparison between all three replicates. E) Heatmap representing the differential expression of cellular RNAs detected by RNAseq in SINV-infected (4 or 18 hpi) and uninfected HEK293 cells. Only RNAs with log2 fold change >3 or <-3 and $p<0.01$ in mock to infected comparisons are displayed. F) Principal component analysis of gene expression profiled by RNAseq in uninfected and SINV-infected cells at 4 or 18 hpi. The three replicates of each condition are considered separately. Data shows that, first, replicates from the same condition cluster together (i.e. they are more similar to each other than to other conditions) and, second, that the transcriptome at 18 hpi strongly differs from the uninfected control and 4 hpi. G) Read counts for viral RNA, cellular protein-coding RNA and cellular non-coding RNA quantified by RNAseq in mock and SINV-infected cells. These plots show the changes in the RNA composition of the cells as infection progresses.

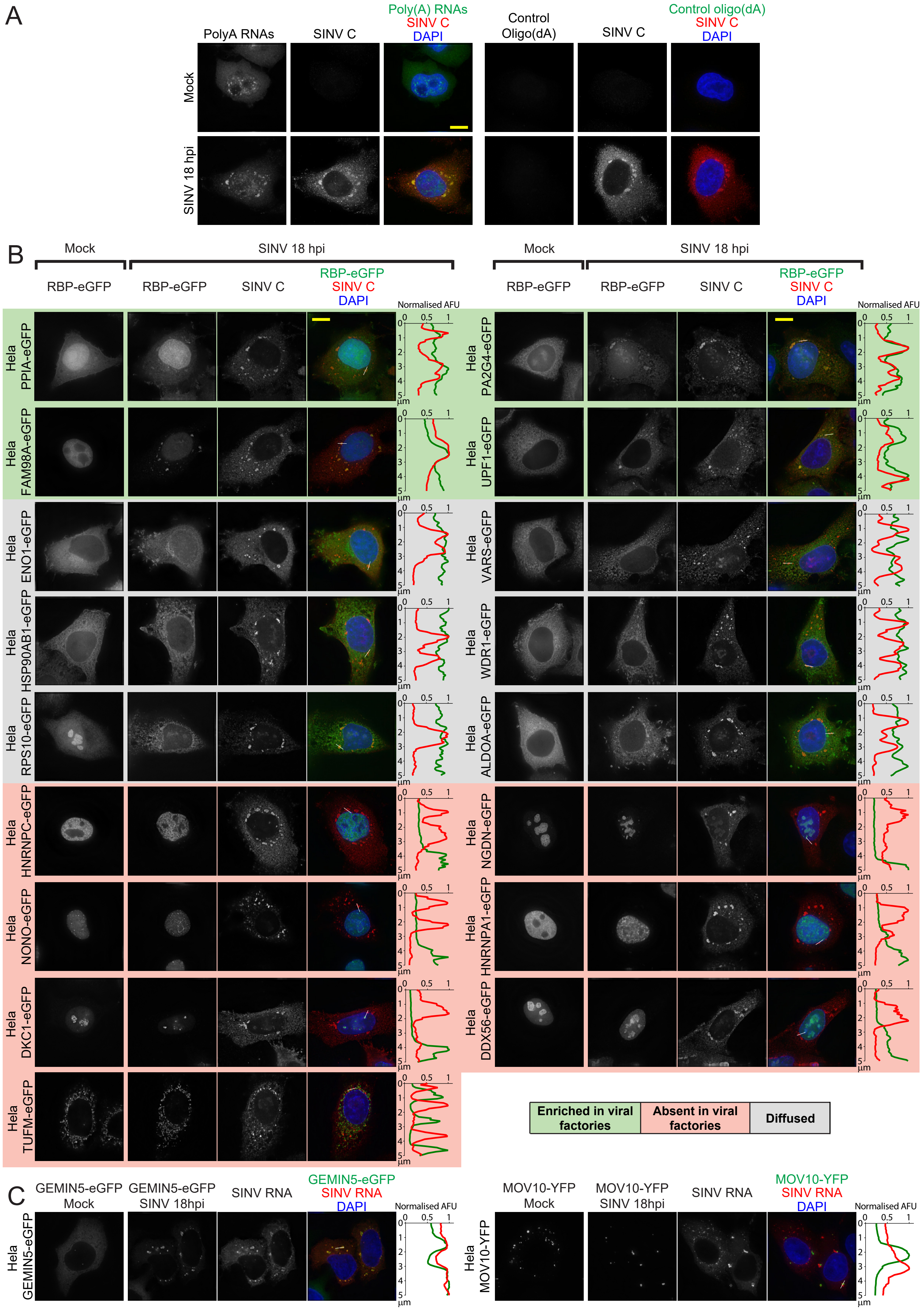

Figure S4 (Garcia-Moreno et al)

Figure S4. Localisation of host RBPs in SINV-infected HeLa cells; related to Figure 4. A) Localisation analysis of poly(A) RNAs and SINV C protein in uninfected and infected cells (18 hpi) by combined fluorescence *in situ* hybridisation with an oligo(dT) probe coupled to Alexa594 and immunofluorescence with an antibody against SINV C. DAPI was used to indicate the position of the nuclei; oligo(dA) probe coupled to Alexa 594 was used as a negative control. At 18 hpi, cytoplasmic poly(A) RNA concentrates in cytoplasmic foci that co-localise with SINV C. This poly(A) RNA is thus likely to be viral. B) Localisation by immunofluorescence of the eGFP-fused RBPs in uninfected or SINV-infected cells (18 hpi). Immunofluorescence with an antibody against SINV C and DAPI staining were used to localise the viral replication factories and the nuclei, respectively. At least 10 fields from at least two biological replicates were inspected for each protein to determine whether the proteins accumulate in the viral factories, are diffused in the cytoplasm or are excluded from the viral factories (indicated with a green, grey or red box, respectively). Plots of green and red fluorescence intensity profiles in a representative 5 µm section (white line) are shown for each protein. AFU, arbitrary fluorescence units. C) Localisation analysis of SINV RNA and GEMIN5-GFP (left panel) or MOV10-YFP (right panel) in uninfected or SINV-infected cells (18 hpi) by combined *in situ* hybridisation and immunofluorescence. Yellow scale-bar represents 10 µm.

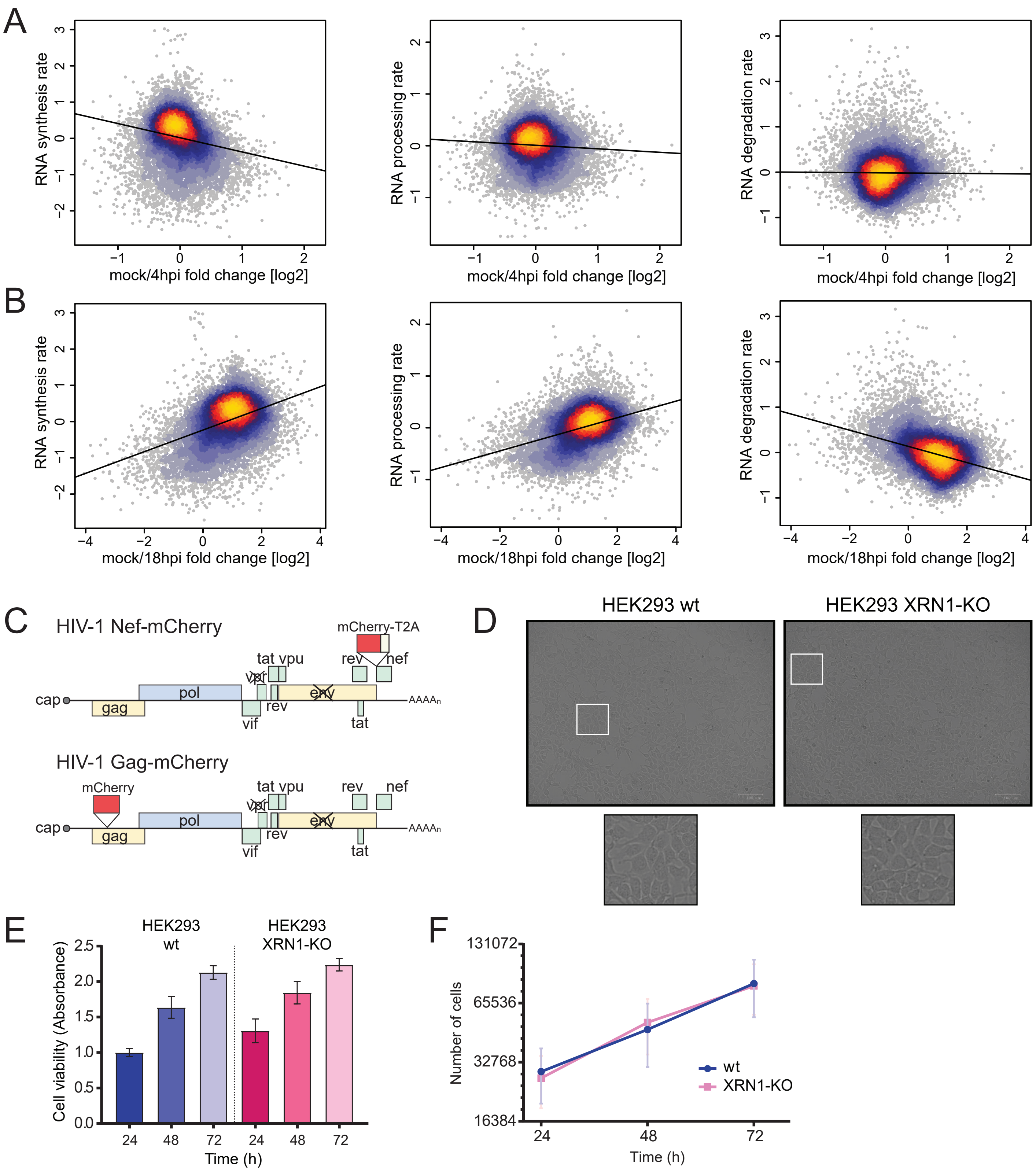

Figure S5 (Garcia-Moreno et al)

Figure S5. Analysis of the contribution of transcription, processing and degradation to the transcriptome of SINV-infected cells; related to Figure 5. A) Plots representing the log2 fold change of cellular RNAs detected by RNAseq between uninfected and SINV-infected (4 hpi) cells, compared to rates of RNA synthesis (left), processing (middle) and degradation (right) determined in (Mukherjee et al., 2017). B) As in (A) but comparing uninfected cells and 18 hpi. C) Schematic representation of HIV-1 single-round replication virus tagged with mCherry in Nef or Gag. D) Bright-field microscopy images of HEK293 wt and XRN1 knock out cells. E-F) 25,000 HEK293 wt or XRN1 knock out cells were seeded per well of a 96-well plate. Cell viability was estimated 24, 48 and 72 h later by adding CellTiter 96 Aqueous One Solution reagent and measuring absorbance at 490 nm on a plate reader (panel E). The number of cells was counted 24, 48 and 72 h after seeding using an automated cell counter (panel F). Values are represented as the mean ± standard deviation of three independent experiments.

A

Target	Compound	Concentration	% viability	SD	p-value
	DMSO	0.1%	100.00	±4.46	
IRE1α	4μ8C	20 μM	78.69	±11.72	0.00056
		30 μM	83.72	±9.11	0.00062
		40 μM	79.29	±9.25	0.00012
HSP90AB1	Ganetespib	100 nM	98.53	±3.10	0.24259
HSP90AB1	Geldanamycin	100 nM	81.49	±8.50	0.00014
		250 nM	81.43	±10.51	0.00068
PPIA	CysA	5 μM	92.87	±6.07	0.00205
PA2G4	WS6	1 μM	100.01	±7.91	0.99804

B

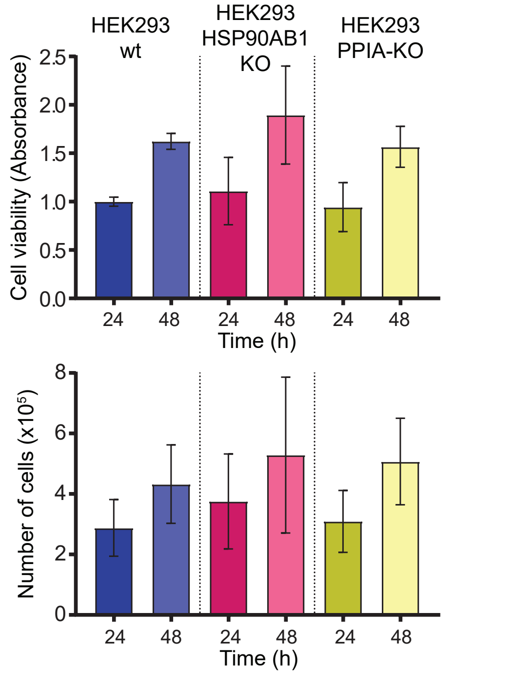

C

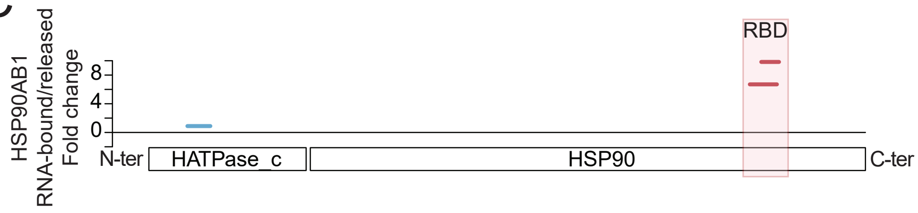

D

E

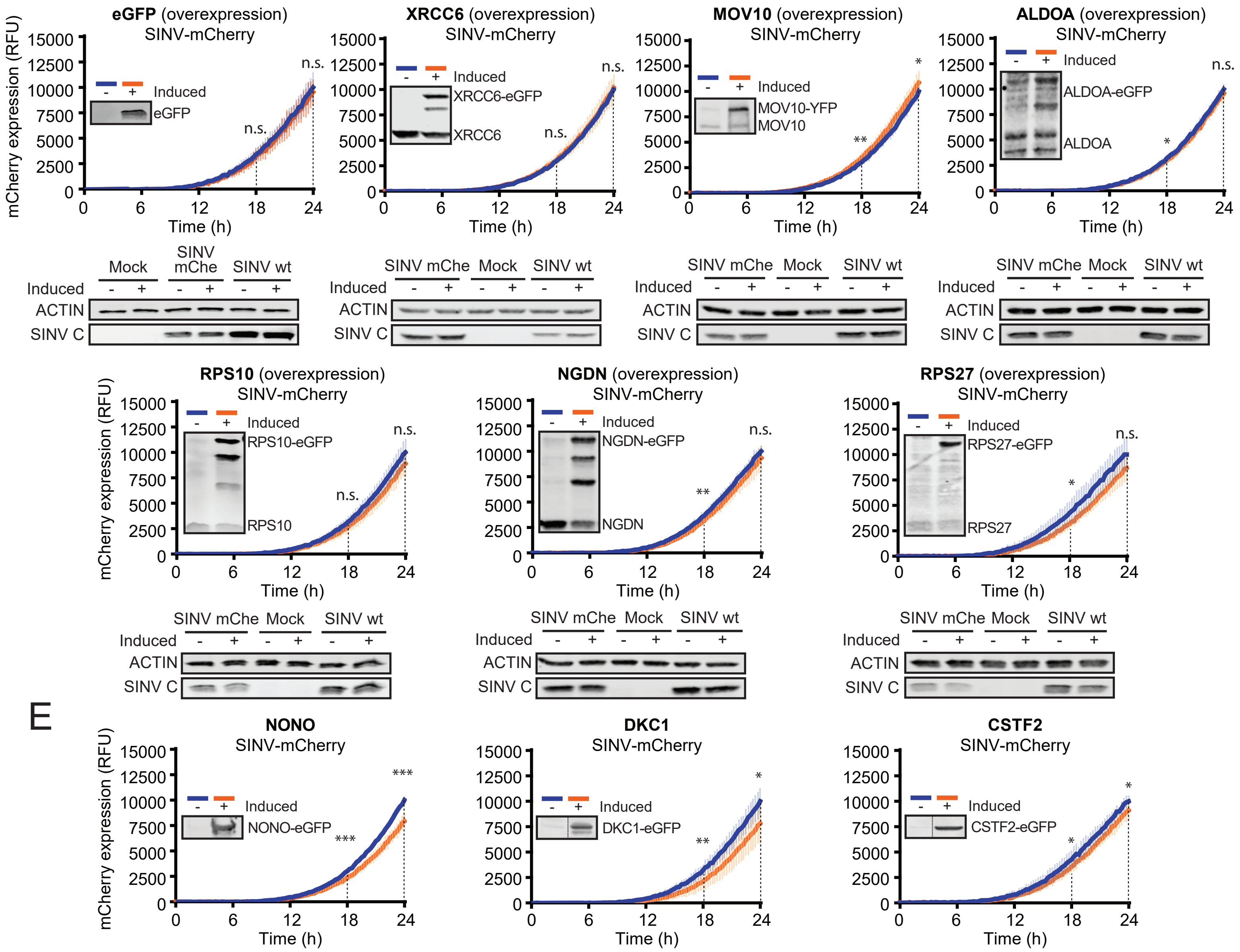

Figure S6 (Garcia-Moreno et al)

Figure S6. Effects of host RBP perturbation on SINV infection; related to Figure 6. A) HEK293 cells were treated with different inhibitors targeting host RBPs and viability was monitored using CellTiter 96 Aqueous One Solution kit. Absorbance at 490 nm was measured on a plate reader. Data is represented as the mean ± standard deviation (SD) of at least three independent experiments performed in triplicate. T test was employed to determine the significance of the changes in viability of cells treated with the inhibitors or dimethyl sulfoxide (DMSO) as a control. Any dose that caused a reduction in cell viability greater that 20% was considered 'toxic'. B) Cell viability (upper panel) and proliferation (lower panel) was analysed as in Figure S5E and S5F, respectively, in HEK293 HSP90AB1 or PPIA knock out cells. C) RNA-binding sites of HSP90AB1 identified by RBDmap (Castello et al., 2016). RBDmap employs UV crosslinking, denaturing lysis, oligo(dT) capture and partial proteolysis to determine in a system-wide manner the protein regions engaged in RNA binding. Y-axis indicates the enrichment of each identified peptide in the RNA-bound fraction and X-axis represents the protein from N- to C-terminus. Boxes below the X-axis indicate the position of annotated protein domains (Pfam). Red lines represent peptides engaged in RNA binding identified with 1% FDR (RBDpeps), while cyan lines indicate peptides that do not bind to RNA. D) Upper panels show the mCherry expression in HEK293 cells overexpressing the candidate RBPs fused to eGFP and infected with SINV-mCherry. Red fluorescence was measured as in Figure S1B. Overexpression was confirmed by western blot analyses with specific antibodies. RFU, relative fluorescence units. Bottom panels show Western blotting analysis of SINV C accumulation in cells overexpressing the candidate RBP-eGFP and infected with SINV or SINV-mCherry for 18 h. E) As in (D) but using eGFP antibody to detect the expression of RBP-eGFP fusion proteins. ***$p<0.001$; **$p<0.01$; *$p<0.05$; n.s. non-significant.

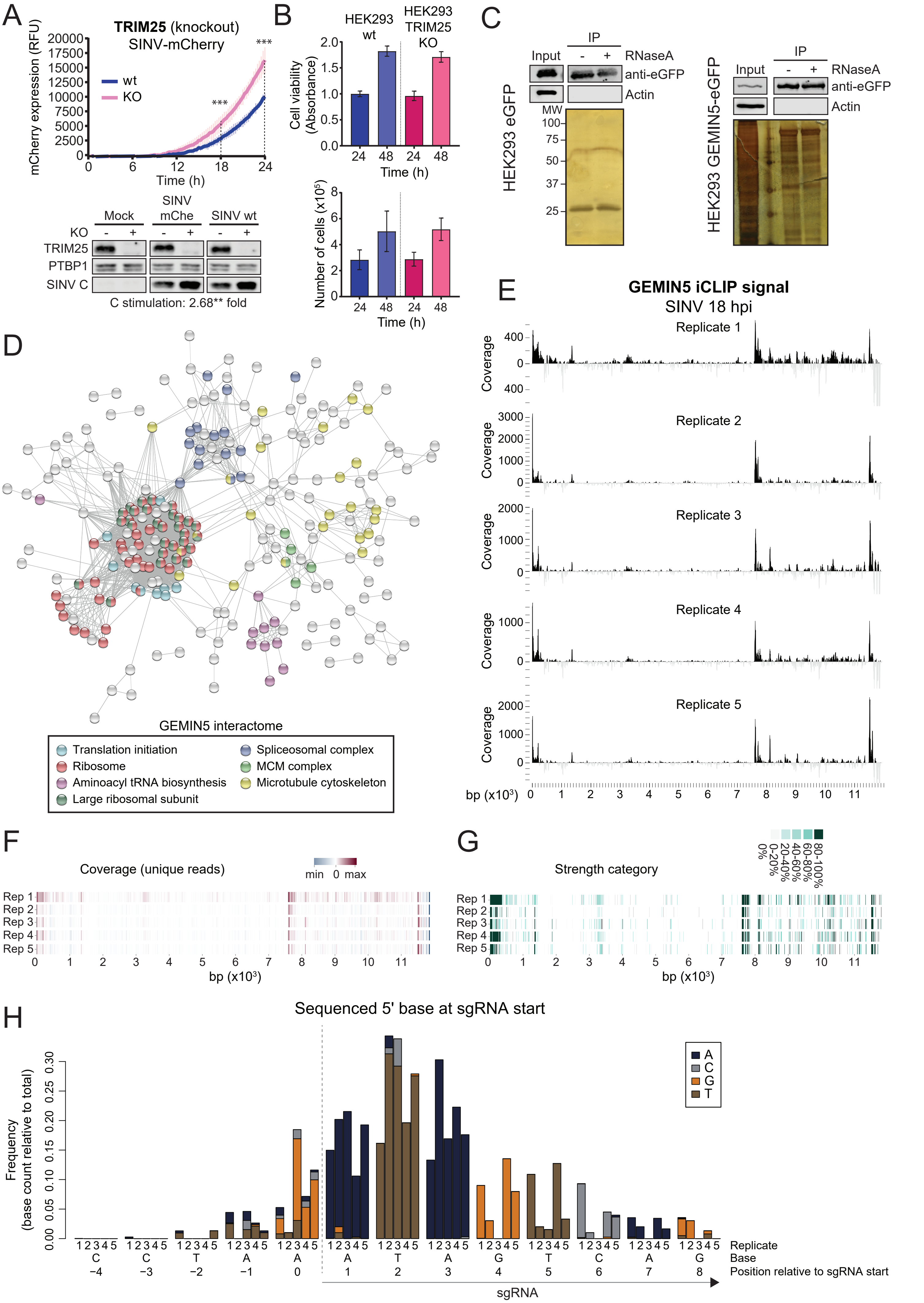

Figure S7 (Garcia-Moreno et al)

Figure S7. Analysis of GEMIN5 molecular partners in SINV-infected cells; related to Figure 7 and Table S5. A) mCherry expression in HEK293 wt and TRIM25 knock out cells infected with SINV-mCherry (upper panel). Red fluorescence was measured as in Figure S1B. RFU, relative fluorescence units. Bottom panels show Western blotting analysis of TRIM25 knock out cells infected with SINV-mCherry or SINV wt for 18 h. Fold change of SINV C (TRIM25 KO vs wt cells) is shown below. ***$p<0.001$; **$p<0.01$. B) TRIM25 knock out cells viability (upper panel) and proliferation (lower panel) was analysed as in Figure S5E and S5F, respectively. C) eGFP (left panels) and GEMIN5-eGFP (right panels) expressed in Tet-on HEK293 cells were immunoprecipitated with GFP-Trap_A. Eluates were analysed by Western blotting with an antibody against eGFP and silver staining. Immunoprecipitation was performed in the presence or absence of RNase A. D) STRING protein-protein interaction map of the proteins enriched in GEMIN5 IPs with $p<0.01$. E) Coverage of 5'-first base of unique sequenced fragments from GEMIN5 iCLIP shown along SINV RNA genome. The data is shown as 20 nt sliding mean of library-size normalised reads (RPM) after subtraction of background observed in GFP control. The five biological replicates are shown individually. F) As in E but in a heatmap representation. G) Binding sites predicted individually for the five replicates, grouped into five categories based on strength of binding (coverage normalised to peak width). H) Count of sequenced 5' base of unique molecules mapping to the region around SINV sgRNA start site relative to total count in the region, shown for the five replicates. Position indicated as ‘1’ is the first base of the SINV sgRNA. The number of counts (binding events) is highest at the region corresponding to the beginning of the sgRNA. Base ‘0’ just before the start of the sgRNA is often sequenced as ‘G’ rather than the genomic ‘A’. These observations suggest that GEMIN5 binding this region is likely to interact with the sgRNA, exclusively or in addition to the overlapping gRNA, and that that additional ‘G’ correspond to the cap structure.
